# A comparative evaluation of data-merging and meta-analysis methods for reconstructing gene-gene interactions

**DOI:** 10.1186/s12859-016-1038-1

**Published:** 2016-06-06

**Authors:** Vincenzo Lagani, Argyro D. Karozou, David Gomez-Cabrero, Gilad Silberberg, Ioannis Tsamardinos

**Affiliations:** Institute of Computer Science, Foundation for Research and Technology – Hellas, Heraklion, Greece; Unit of Computational Medicine, Department of Medicine, Karolinska Institutet, 171 77 Stockholm, Sweden; Center for Molecular Medicine, Karolinska Institutet, 171 77 Stockholm, Sweden; Computer Science Department, University of Crete, Heraklion, Sweden; Unit of Clinical Epidemiology, Department of Medicine, Karolinska University Hospital, L8, 17176 Heraklion, Sweden; Science for Life Laboratory, 17121 Solna, Sweden

**Keywords:** Gene-network Reconstruction, Meta-Analysis, Batch-effect Removal, Surrogate Variable Analysis, Integrative Analysis, Escherichia coli, Yeast, Peripheral Blood Mononuclear Cells, Ikaros Transcription Factor

## Abstract

**Background:**

We address the problem of integratively analyzing multiple gene expression, microarray datasets in order to reconstruct gene-gene interaction networks. Integrating multiple datasets is generally believed to provide increased statistical power and to lead to a better characterization of the system under study. However, the presence of systematic variation across different studies makes network reverse-engineering tasks particularly challenging. We contrast two approaches that have been frequently used in the literature for addressing systematic biases: *meta-analysis methods*, which first calculate opportune statistics on single datasets and successively summarize them, and *data-merging methods*, which directly analyze the pooled data after removing eventual biases. This comparative evaluation is performed on both synthetic and real data, the latter consisting of two manually curated microarray compendia comprising several *E. coli* and Yeast studies, respectively. Furthermore, the reconstruction of the regulatory network of the transcription factor Ikaros in human Peripheral Blood Mononuclear Cells (PBMCs) is presented as a case-study.

**Results:**

The meta-analysis and data-merging methods included in our experimentations provided comparable performances on both synthetic and real data. Furthermore, both approaches outperformed (a) the naïve solution of merging data together ignoring possible biases, and (b) the results that are expected when only one dataset out of the available ones is analyzed in isolation. Using correlation statistics proved to be more effective than using *p*-values for correctly ranking candidate interactions. The results from the PBMC case-study indicate that the findings of the present study generalize to different types of network reconstruction algorithms.

**Conclusions:**

Ignoring the systematic variations that differentiate heterogeneous studies can produce results that are statistically indistinguishable from random guessing. Meta-analysis and data merging methods have proved equally effective in addressing this issue, and thus researchers may safely select the approach that best suit their specific application.

**Electronic supplementary material:**

The online version of this article (doi:10.1186/s12859-016-1038-1) contains supplementary material, which is available to authorized users.

## Background

Reverse engineering of gene regulatory network is a vibrant research area [1], whose scope is reconstructing the biological mechanisms underlying gene activity. Several types of statistical models and algorithms have been proposed for deriving and representing gene interaction networks [2]. Relevance networks [3] are one of the most basic models, where gene pairs showing highly significant correlation in their expression values are assumed to be functionally associated.

Unfortunately, this assumption is not valid when data from different studies are integratively analyzed. Systematic biases across studies can originate spurious correlations that do not actually reflect any interactions among genes. On the other side, they can hide associations that are actually present among the measured quantities [4]. These systematic variations are usually known as “batch-effects”, and they can arise even when all studies share the same experimental design and measure the same quantities. The name originates from systematic biases that are present across “sample batches” within single studies, due to small differences in the processing of each batch [5].

*Meta-Analysis* (MA, [6]) and *Data-Merging* (DM [7]) are two approaches widely employed in the literature for addressing systematic variations in studies that share the same experimental design. In MA statistical methods are separately applied on each dataset for obtaining statistics of interest, e.g., differential expression p-values. The results from each study are then combined for creating summary statistics. The latter approach merges samples from different studies in a unique dataset, on which subsequent analyses are performed. While MA methods implicitly take in account batch-effects, DM require suitable *Batch-Effect Removal* (BER) algorithms [8].

In this work we compare meta-analysis and data-merging methods in the context of retrieving gene-gene interactions in compendia of microarray studies. To this scope we compiled two different collections of microarray experiments, containing 11 and 7 studies on *Escherichia coli* and *Yeast*, respectively. For each collection we identified candidate interactions for multiple transcription factors by combining relevance networks with meta-analysis and data-merging methods, in turn. The candidate interactions are then compared against lists of known, experimentally verified interactions, in order to contrast the effectiveness of MA and DM methods in retrieving actual relationships.

The comparison between the two approaches is furthermore deepened on synthetic data, where a large variety of scenarios is simulated across different networks, levels of systematic bias, number of considered studies and number of samples in each study. All experimentations underlined that batch-effects are detrimental for the analyses, and that MA and DM prove similarly effective in addressing issues arising from systematic variations.

Finally, we present an application on human Peripheral Blood Mononuclear Cells (PBMCs), for the reconstruction of the Ikaros transcription factor regulatory network. For this specific application we used a Bayesian-Network, constraint-based learning approach in place of relevance networks, providing evidences that the results of this study transfer on more complex network-learning approaches.

### Related work

To the best of our knowledge, there is no other study that systematically contrasts MA and DM methods in the context of retrieving gene-gene interactions. Several studies exist that evaluate the relative performances of MA methods for gene network reconstruction [9–14]. In short, it is not possible to rigorously come to a unique conclusion regarding the best meta-analysis algorithm for network reconstruction. The observed discrepancy among these studies is a result of numerous factors, including data complexity and heterogeneity, difficulties in determining a golden truth, and the inclusion of a limited number of meta-analysis approaches in the experimentations.

The most common MA techniques applied in the spectrum of gene network reconstruction are based on Fisher’s method [15, 16], vote counting approaches [17–19], fixed and random effect sizes [20]. Segal et al. [21] was the first one that marched towards unlocking hidden biological knowledge by using meta-analysis for network reconstruction. Numerous approaches then followed, as described in [22]. In all cases, meta-analysis approaches seemed to perform better than individual reverse-engineering methods.

Similarly, the applicability of data-merging methods in the context of network reverse engineering has been investigated in several works [5, 23–27]. In earlier studies, the vast majority merely used normalization methods to merge the compendium of expression data [23, 28]. Robust Multi-Array Average method (RMA) [27–29] seemed to outperform other normalization methods such as linear scaling procedures based on the median signal intensity [30], quantile normalization through MAS algorithm [31], GCRMA [32], Dchip PM [33]. However, RMA normalization proved to be ineffective in removing batch effects which affect particular genes and may affect different genes in different ways [5].

Recent approaches have been developed for identifying and removing batch effects [8, 24, 34] but have not been widely used. Such approaches include ComBat [35], Surrogate variable analysis (SVA) [36], Distance-weighted discrimination (DWD) [37], Mean-centering (PAMR) [38], and Geometric ratio-based method [39]. In relevant studies, ComBat seems to outperform these methods as it robustly manages high dimensional data with small sample sizes. A previous attempt to evaluate the effectiveness of batch adjustment methods was made by the MAQC-II project [40]. It is necessary to bear in mind that even the most effective batch effect removal method cannot sufficiently reduce the batch effects in cases of poor experimental design [41].

The literature regarding MA and DM application in the context of differential expression is particularly rich [6, 42–48], and a complete review is out of the scope of the present work. We point out that we found only a single study [49] that directly compares the performances of the two approaches on finding differentially expressed genes. Interestingly, this study concludes that both approaches achieve comparable results.

## Methods

### Experimentation protocol

**Fig. 1:**
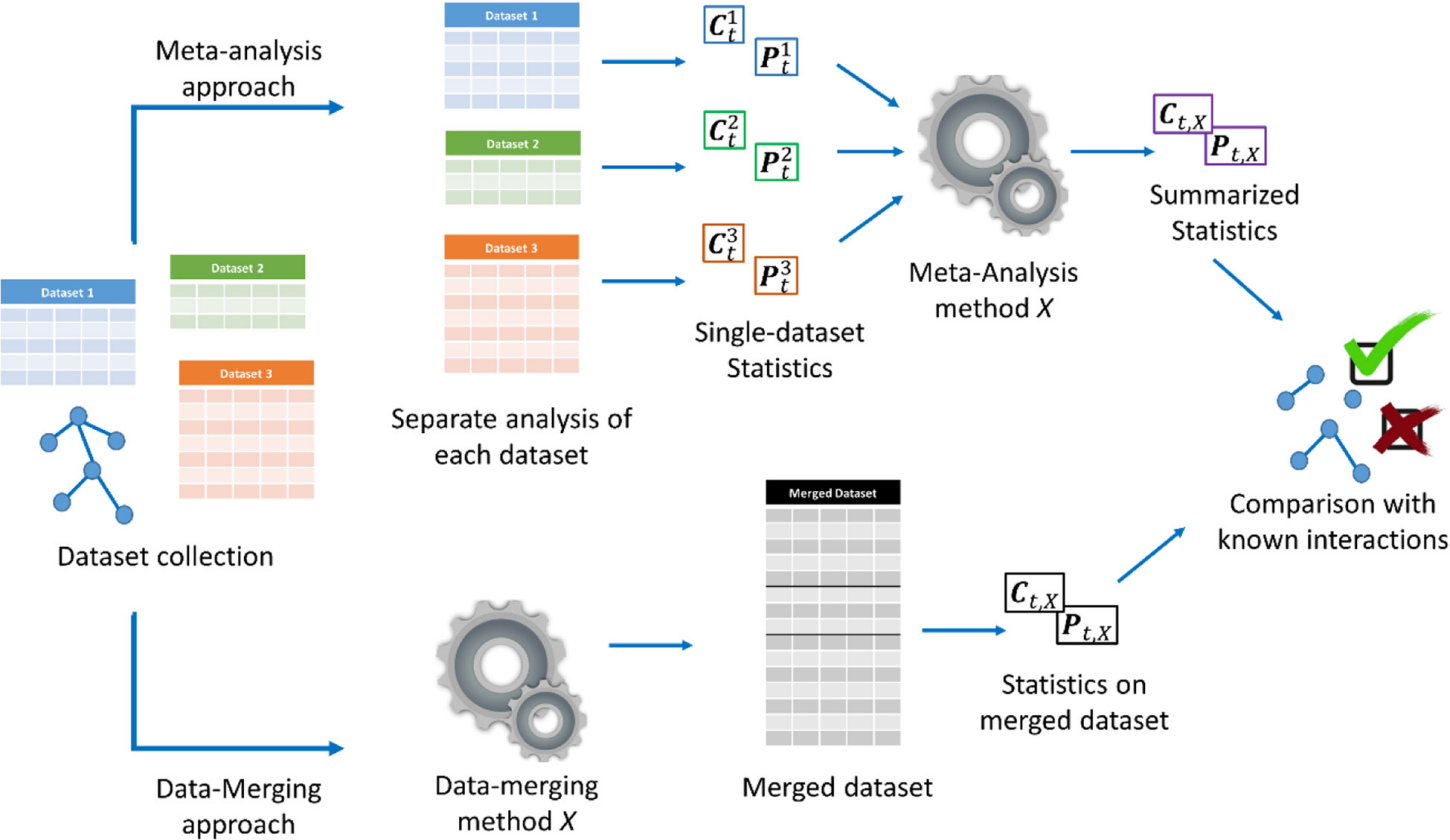
Experimentation protocol schematic representation. A collection of microarray dataset is assumed to be generated from multiple, independent studies all following the same experimental protocol and measuring the same quantities. The studies investigate the same biological system regulated by an unknown gene interaction network. The data collection is analyzed with two different approaches, namely Meta-Analysis and Data-Merging. In the first approach correlations among transcription factors and genes are first calculated on each dataset and then summarized, while in the latter the data are merge together, corrected for eventual batch-effects and the correlations are estimated on the pooled data. The correlations retrieved by the two approaches are then compared with a set of known interactions that partly and possibly noisy reconstruct the original gene interaction network. Meta-Analysis and Data-Merging approaches are then evaluated on the basis of their ability of assign highly-significant correlations to known interactions

We devised a large experimentation in order to compare MA and DM methods in several scenarios, meaning over different biological systems, levels of systematic bias, number and composition of available studies. For each scenario we followed the same experimentation protocol, detailed below and presented in Fig. 1 as well.

Let ***M*** be a collection (or compendium) of *m* microarray datasets. All studies in ***M*** follow the same experimental protocol, analyze the same type of biological specimens, and measure the same *n* expression values (*probesets*). However each dataset *D*_*j*_ includes a separate set of *s*_*j*_ samples. This means that each study in ***M*** investigates the same gene-regulatory network, and that the data of all studies have been generated according to this network. Thus, any systematic bias across datasets should be due to (unknown) technical differences occurred during the measurement process or to the presence of confounding factors.

For each collection ***M*** there is a set ***T*** = {*TF*_1_, *TF*_2_, …, *TF*_*t*_, …, *TF*_|***T***|_} of |***T***| transcription factors of interest. We assume to know the list ***I***_*t*_ of genes that interact with each transcription factor *TF*_*t*_, i.e., ***I***_*t*_ contains all genes that are targets of *TF*_*t*_ along with the genes that regulate *TF*_*t*_.

We apply a relevance network approach for retrieving these known interactions. In detail, for each collection ***M*** and each transcription factor *TF*_*t*_ the correlations among the expression values of *TF*_*t*_ and the remaining *n* − 1 probesets are calculated over all datasets in ***M***, using in turn an MA or DM approach. MA algorithms separately compute the correlations on each dataset and then summarize them, while DM methods pool together the data from all datasets and directly compute the final correlation values.

Let *C*_*t*,*i*,*X*_ be the correlation between transcription factor *t* and probeset *i* produced with the MA or DM method *X*, and *P*_*t*,*i*,*X*_ the p-value assessing the null hypothesis *H*_0_ : *C*_*t*,*i*,*X*_ = 0. The set of *n* − 1 correlations (*p*-values) for transcription factor *t* is indicated as ***C***_*t*,*X*_(***P***_*t*,*X*_). Both ***C***_*t*,*X*_ and ***P***_*t*,*X*_ are sorted according to the absolute values of the correlations, so that the most relevant associations appear at the top of both vectors.

Relevance networks postulate that genes included in ***I***_*t*_ should be strongly correlated with TF_t_, therefore *MA and DM methods are evaluated with respect to their ability of assigning highly significant correlations to known interactions.* Different metrics are used to compare each ***C***_*t*,*X*_ against its corresponding **I**_t_, and DM / MA approaches are ranked according to their respective performances.

The following sections describe in detail the experimental and synthetic data collections used in the experimentations, along with the algorithms, correlation measures and performance metrics included in the analysis.

All simulations and analyses were performed in the *R* software [50].

### Data

#### Escherichia coli data compendium

The regulatory network of the Escherichia coli (E. coli) K-12 bacterium has been extensively studied [51], and consequently it is an ideal test bed for our experimentation. Studies in the GEO repository on E. coli comprising more than twenty expression profiles and using the Affymetrix E. coli Antisense Genome Array were taken in consideration for inclusion in the analysis. Imposing a single microarray platform ensures that all datasets measure the same probesets. Studies applying experimental interventions known to artificially disrupt gene-gene interactions, as for example gene knock-out, were excluded from the compendium. Eleven studies were included in the collection, whose characteristics are reported in the (Additional file 1: Table S1), for a total of six-hundred eighteen samples measured under a variety of conditions. Probesets without annotations were excluded from the analysis, leaving a total of 4088 probesets, each corresponding to a specific gene (no gene was measured by multiple probesets).

The RegulonDB database was used in order to retrieve known TF-gene interactions in the E. coli regulation program [52]. This database publicly and freely provides more than 4131 transcriptional regulatory interactions, manually retrieved and curated from the literature. Interestingly, each interaction is assigned to an evidence class, ranging within the levels ‘weak’, ‘strong’ and ‘confirmed’. The level of evidence is determined by the experimental method used in the original study reporting the interaction. Experimental procedures where false positives are prevalent, like computational predictions or gene expression analysis, are catalogued as ‘weak’. Other procedures providing evidence of physical interaction or anyhow excluding explanations alternative to a gene-gene interaction (e.g., site mutation [53]) are considered ‘strong’. When a regulatory relationship is supported by multiple, independent strong evidences, then it is classified as ‘confirmed’.

Preliminary experimentation including all RegulonDB regulatory relationships led to poor results, close to random guessing (results not shown). We hypothesized that large number of false positives in the weak interactions could negatively affect the results, thus we decided to exclude them from the analysis, leaving a total of 2475 strong and confirmed regulatory relationships.

Finally, we decided to consider only transcription factors having at least three known interactions, for a total of 124 genes included in ***T***_*EColi*_.

#### Yeast data compendium

The same criteria used for compiling the E. coli compendium were used for building a collection of seven Yeast datasets, all measured with the Affymetrix Yeast Genome S98 Array platform and containing a total of four-hundred twenty seven (427) samples (Additional file 1: Table S2). A total of 4218 probesets were not associated with a given gene name, and 149 genes were associated to more than one probeset. We removed non-annotated genes and we randomly selected a single probeset for genes with multiple measurements, leaving a total of 4944 probesets.

Known interactions were retrieved from Yeastract [54], which is the largest database of this type for the yeast organism to date, with more than 200,000 reported gene-gene interactions. Similarly to RegulonDB, Yeastract lists manually curated regulatory relationships retrieved from the literature, and it also provides information about the experimental procedure used for assessing each reported interactions. We again required ‘strong’ evidence, leaving 257 gene-gene known and reliable interactions in the analysis. Also for this compendium genes with at least three known interactions were included in ***T***_*Yeast*_, for a total of 44 transcription factors.

#### Synthetic data

Several collections of synthetic datasets were produced for better characterizing MA and DM performances under different scenarios.

Data were sampled from artificial networks specifically devised in order to resemble real-life gene regulatory programs, following the scale-free theory introduced by Barabási [55]. According to this theory, biological networks are not randomly organized, and the number of connections incident to a node is regulated by the power law *P*(*k*) ~ *k*^− *γ*^, where *k* is the number of interactions, *γ* is a parameter whose value depends by the specific domain, and *P*(*k*) is the fraction of genes having *k* connections. In other words, real-world gene regulatory programs have few transcription factors (hubs) that regulate large numbers of genes, while the remaining nodes have relatively few connections. Each synthetic network is represented by a Direct Acyclic Graph (DAG) composed by a set of nodes (genes) *V* = {1, …, *n*} and a set of directed edges *E* = {(*i*, *j*)}. If the edge (*i*, *j*) is present in the network, then gene *i* is a parent of (regulates) gene *j*. The set of parents of node *j* is indicated as *IN*(*j*).

These artificial networks were equipped with a parameterization suitable for the simulation of gene expression data and batch effects. Each gene *i* was associated with a baseline expression value *α*_*i*_ uniformly sampled in the interval [0, 1], while each edge (*i*, *j*) is equipped with a randomly generated coefficient *β*_*ij*_ ∈ [−1, − 0.5] ∪ [0.5, 1] representing the strength of the interactions between *i* and *j*.

Batch-effects across studies are assumed to be composed of an additive and a multiplicative component, following an approach already used in [24]. The first component shifts the gene average value, while the multiplicative error intensifies the sample-specific variance.

The expression value *y*_*sjk*_ for sample *s*, gene *j* and study *k* is generated as follows:

$$ {y}_{sjk}={\alpha}_j+{\displaystyle \sum_{i\in IN(j)}}{\beta}_{ij}{y}_{sik}+{\epsilon}_{sj}+{\gamma}_{jk}+{\delta}_{jk}{\epsilon}_{sj}^{\hbox{'}} $$

According to this formula, each expression value *y*_*sjk*_ is a linear combination of its baseline value *α*_*j*_ and the expression values of its regulating genes *y*_*sik*_, *i* ∈ *IN*(*j*). The quantity ϵ_*j*_ is random noise distributed as *N*(0, 1) (normal distribution with zero mean and unitary standard deviation) that represents unmodeled regulatory mechanisms that concur in determining the expression of the gene. The two factors *γ*_*jk*_ and *δ*_*jk*_ϵ_*sj*_^'^ respectively represent the addictive and multiplicative component of the systematic bias in study *k*, and are both randomly sampled from the distribution *N*(*τ*, *τ*). The random variable ϵ_*j*_^'^ is again distributed as *N*(0, 1).

During our experimentations, five independent synthetic networks with four-thousand nodes each were created using the *barabasi.game* function from the R package igraph [56]. For each network we simulated different compendia by varying the number of studies in [5, 10, 50], the number of samples for each study in [5, 20, 50], and the hyper-parameter *τ* controlling the level of systematic bias in [0.1, 0.5, 1], thus obtaining 27 different scenarios for each network and 135 in total.

Finally, for each network the list ***T*** of transcription factors includes all genes directly connected to at least twenty other genes. This leads to an average of 18 transcription factors for each network, each one connected on average with 40 genes. We consider only direct interactions in order to ensure that the corresponding associations are strong enough to be effectively retrieved from the data.

### Relevance networks reconstruction

When a single dataset is available, the relevance network for the transcription factor *TF*_*t*_ can be easily reconstructed by computing the vector ***C***_*t*_ containing *n* − 1 associations between *TF*_*t*_ and all other probesets *i*. These association measures are eventually coupled with measures of statistical significance ***P***_*t*_, and the genes ***Q***_*t*_ belonging to the reconstructed network can be selected by imposing an appropriate decision threshold *θ* to either the association or the significance values. When multiple datasets are available, the same procedure can be followed with the vectors ***C***_*t*,*X*_ and ***P***_*t*,*X*_ computed through the meta-analysis or data-merging method *X* (see Fig. 1).

In our experimentations we use in turn the Pearson and Spearman correlation measures [57, 58] for estimating the association values *C*_*t*,*i*_. Pearson correlation quantifies the association between two random variables *x* and *y* as

$$ {\rho}_{x,y} = \frac{{\displaystyle \sum}\left({x}_i-\overline{x}\right)\ \left({y}_i-\overline{y}\right)}{s_x{s}_y} $$

where $\overline{x},\overline{y}$ are sample means and *s*_*x*_, *s*_*y*_ sample standard deviations. The Spearman correlation uses the same formula on *x* and *y* rankings. The null hypothesis *H*_0_ : *ρ*_*x*,*y*_ = 0 can be properly assessed for both correlation measures [59, 60].

### Performances metrics

The correlation values ***C***_*t*,*X*_ and corresponding p-values ***P***_*t*,*X*_ are compared with the list of known interactions ***I***_*t*_ in order to assess *X* effectiveness in correctly retrieving gene regulatory relationships. In the ideal case high correlations would be assigned exclusively to actual interactions, while any other gene-pair would be reported as weakly associated. However, in real cases ***I***_*t*_ is probably incomplete and noisy, undermining a fair evaluation. Moreover, only a handful of regulatory relationships are usually known for each gene, while the number of possible gene-pairs is two or three order of magnitudes larger, dramatically increasing the possibility of retrieving false positives due to mere multiple-testing issues.

In order to better characterize the performances of each method, we adopted several metrics commonly used in the machine-learning area of Information Retrieval, a field whose operational settings strictly resemble the one depicted above [61].

The Receiver Operator Characteristic (ROC) Area Under the Curve (**AUC**, [62]) is a metric that integrate sensitivity and specificity information for all possible values of the decision threshold *θ*. The AUC ranges in the interval [0, 1], where one corresponds to perfect rank (i.e., all true interactions are at the top of ***C***_*t*,*X*_), 0.5 corresponds to random ordering and zero to perfectly inverted predictions. Interestingly, AUC values can be interpreted as the probability of correctly ranking two randomly selected interactions according to their status (true/false interaction).

The Area Under the Precision Recall Curve (**AUPRC,** [63]) is similar to the AUC and summarizes precision and recall information for varying *θ*. With respect to AUC, AUPRC has demonstrated to have higher discriminative power when very few positive cases (true interactions) are available [64].

Both AUC and AUPRC evaluate the whole list of correlation values, providing a measure of *global* performance. However, researchers using network reconstruction algorithms often restrict their attention to a few predicted gene-gene interactions, the ones deemed more reliable. These interactions are ideal candidate for subsequent in vitro or in vivo experimental validation, which are usually too expensive or demanding to be performed on all predictions.

Thus, we are interested in evaluating the *partial* performances of the methods on the interactions corresponding to the highest correlations in ***C***_*t*,*X*_. To this end we use a version of AUC known as partial AUC (**pAUC**, [65]), which considers a restricted region of the whole sensitivity / specificity curve (specificity in [0, 0.2] for our experimentations). The McClish formula [65] standardizes pAUC values in [0, 1], allowing the pAUC to have the same probabilistic interpretation of the AUC.

We also devised a new metric that is specific for assessing partial performances, namely the Area Under the False Discovery Rate (**AUFDR**). Let ***Q***_*t*,*X*,*R*_ be the list of *R* interactions with highest correlation according to ***C***_*t*,*X*_. The AUFDR integrates the proportion of correctly predicted interactions in the range [1, *R*], i.e., $AUFDR={\displaystyle \sum_{i=1:R}}\frac{{\boldsymbol{Q}}_{t,X,i}\cap {\boldsymbol{I}}_t}{i}$, and it is subsequently normalized in order to assume values in [0, 1], with one indicating that all top *R* predictions are known interactions.

In all our analysis we use in turn vectors ***C***_*t*,*X*_ and ***P***_*t*,*X*_ for evaluating methods’ performances. Highly significant associations often corresponds to p-values that are indistinguishable from zero at machine precision, leading to ties in ***P***_*t*,*X*_ that severely affect performance computations. In contrast, the vector ***C***_*t*,*X*_ does not suffer from this drawback, varying in ranges that seldom include particularly low values. The impact of these issues on performance assessment is discussed in detail in the Result section.

### Integrative approaches

The meta-analysis, data-merging and baseline approaches included in the experimentation are now explained in detail. Table 1 provides a summary of the methods.

**Table 1:** MA, DM and baseline methods included in the experimentations. For each method a synthetic description is provided describing its main characteristics

Approach	Method	Description
Meta-Analysis	Fisher	Combines *p*-values in a statistic that follows a *χ* ^2^ distribution.
	Stouffer	Transforms p-values in Z-scores and merges them with a weighted average
	Fixed-Effects	Assumes all studies measure the same effect and combines estimates with a weighted average
	Random-Effects	Combines estimated effects by assuming that each study measures a biased version of the true effect
	FR-Effects	Estimates whether Fixed or Random-Effects assumptions hold and use one of the two methods accordingly
	Rank-Product	Combines statistics’ ranks by multiplication.
Data-Merging	SVA	Provides surrogate variables that approximate the effect of confounding factors and batch-effects present in the data
	Combat	Assumes additive and multiplicative batch-effects and estimates them by pooling information across genes
	RMA	Normalizes data across expression profiles using Quantile Normalization
	RMA-Combat	Applies RMA and Combat one after the other
	Scaling	Scales the value of each gene in each study to have zero mean and unitary standard deviation
	No-Correction	Merges samples from all studies in a single dataset without any correction
Baseline	Single-Datasets	Computes the performance that is expected by analyzing a single, randomly chosen dataset
	Random-Guessing	Produces randomly sampled correlation values

#### Meta-analysis

Meta-analysis has been described as “the process of synthesizing data from a series of separate studies” [66]. A typical MA application investigates a set of statistics (e.g., p-values) derived in different studies and produces a summary statistic, for example a weighted average (see Fig. 1). Other, sophisticated MA approaches exist for more complex applications, for example meta-regression [67], where differences in the design of the studies or the sampling strategy are treated with a regression approach.

The MA methods used in this study can be thought as a function accepting correlations *C*_*t*,*i*_^1^, …, *C*_*t*,*i*_^*m*^ between gene *i* and transcription factor *t* computed over studies 1 … *m*, as well as their corresponding *p*-values *P*_*t*,*i*_^1^, …, *P*_*t*,*i*_^*m*^, and producing a single statistic and *p*-value:

$$ \left[{C}_{t,iX},{P}_{t,i,X}\right] = f\left({C}_{t,i}^1, \dots,\ {C}_{t,i}^m,\ {P}_{t,i}^1, \dots,\ {P}_{t,i}^m\right) $$

We selected from the literature five MA methods whose operation matches the above definition and that are based on different assumptions and theoretical backgrounds.

- **Fisher** method [68] is one of the first known MA approaches. Under the assumption that all *P*_*t*,*i*,*X*_^1^, …, *P*_*t*,*i*,*X*_^*m*^ assess the same null-hypothesis in multiple, independent studies following an identical design, then the quantity ${\chi}_{t,i}^2=-2\cdotp {\displaystyle \sum_j} \log \left({P}_{t,i}^j\right)$ follows a *χ*^2^ distribution with 2 · *m* degrees of freedom, and can be used for calculating the summarized *p*-value *P*_*t*,*i*,*Fisher*_. We set *C*_*t*,*i*,*Fisher*_ = *χ*_*t*,*i*_^2^.
- **Stouffer** method [69] is conceptually similar to Fisher’s, although it combines Z-scores defined as *Z*_*t*,*i*_^*j*^ = Φ^− 1^(*P*_*t*,*i*_^*j*^) instead of *p*-values. Φ^− 1^ is the inverse of the standard normal cumulative distribution function, and the statistic ${Z}_{t,i}=\frac{{\displaystyle {\sum}_j}{Z}_{t,i}^j}{\sqrt{m}}$ follows a standard normal distribution that can be used for deriving *P*_*t*,*i*,*Stouffer*_. Also in this case *C*_*t*,*i*,*Stouffer*_ = *Z*_*t*,*i*_
- **Fixed-Effects** approach [70] assumes that all studies investigate the same correlation *Ĉ*_*t*,*i*_, whose estimation is biased by a study-specific error factor, i.e., *C*_*t*,*i*_^*j*^ = *Ĉ*_*t*,*i*_ + *ϵ*_*j*_, *j* = 1 … *m*. On the basis of these assumptions, *C*_*t*,*i*,*Fixed*_ can be computed through a weighted mean

$$ {C}_{t,i, Fixed}=\frac{{\displaystyle {\sum}_j}{w}_j\cdotp {C}_{t,i}^j}{{\displaystyle {\sum}_j}{w}_j} $$

where the weights *w*_*j*_ are inversely proportional to the correlations variances. *P*_*t*,*i*,*Fixed*_ is computed by comparing *C*_*t*,*i*,*Fixed*_ Fisher z-transformation against its theoretical normal distribution [71].

- **Random-Effects** models do not assume that each study estimates the same correlation *Ĉ*_*t*,*i*_; the datasets are assumed to be enough ‘similar’ to be jointly analyzed, but at the same time the ground truth correlation ${\widehat{C}}^j{}_{t,i}$ may differ across studies. Particularly, ${\widehat{C}}^j{}_{t,i},\ j=1\dots m$ are assumed to be sampled from a distribution with mean *Ĉ*_*t*,*i*_ and unknown variance $\widehat{\tau}$, while in turn each *C*_*t*,*i*_^*j*^ is an estimation of its corresponding ${\widehat{C}}^j{}_{t,i}$ subject to a study-specific error *ϵ*_*j*_, i.e., ${C}_{t,i,X}^j={\widehat{C}}^j{{}_{t,i}}_{,X}+{\epsilon}_j$.
  The summary correlation *C*_*t*,*i*,*Random*_ is estimated with the Fixed-Effects weighted average, with the weights *w*_*j*_ computed as inversely proportional to the sum of the study-specific and between-study variance, i.e., ${w}_j=\frac{1}{v_j+\widehat{\tau}}$. Interestingly, if all studies share the same ground truth effect (i.e., $\widehat{\tau}=0$), then the Random-Effects model reduces to the Fixed-Effects one.
- **The Rank-Product** method differs from the previous approaches since it combines *correlation ranks* instead of correlations or *p*-values [72]. The vector ***C***_*t*_^*j*^ containing the correlations between the transcription factor *t* and all other probesets in study *j* can be easily converted in a vector of ranks ***R***_*t*_^*j*^, where higher correlations rank first. The Rank-Product method combines ranks *R*_*t*,*i*_^1^, …, *R*_*t*,*i*_^*m*^ from different studies by multiplying them: ${R}_{t,i, Rank-\mathit{\mathsf{Product}}}={\displaystyle {\prod}_j{R}_{t,i}^j}$. True gene-gene interactions are then expected to be placed on the top of the vector ***R***_*t*_ of combined ranks.
  The Rank-Product is actually a special case of a larger family of rank-based methods [73], differing among each other mainly for the formula used for combining the single ranks (e.g., summation, average, product). Some authors have reported that rank-based methods can provide more reliable results than classical MA methods when heterogeneous datasets are analyzed together [74].
  A common drawback of these methods is that statistical significance must be assessed through permutation-based procedures, which usually are quite computationally demanding. However, in this study we adopt a recently introduced formula [75] for computing approximate, yet accurate *p*-values for the Rank-Product results.

These five approaches were implemented in *R* and included in the analyses. Moreover, we included one further method, namely the **FR-Effects** model, based on a combination of Fixed and Random-Effects models. In short, the FR-Effect model first estimates $\widehat{\tau}$, and if the between-study variance is significantly different from zero (Cochran’s Q test [76] *p*-value < 0.1) the Random-Effects model is used, otherwise the Fixed-Effects is used.

#### Data-merging

In contrast with meta-analysis, the data-merging approach pools all data together and then estimates statistics on the resulting dataset. Expression profiles measured in different studies, or even in the same study but in different batches, present systematic variations in their distribution [8], and these variations are detrimental for the analysis. Batch-effect removal methods attempt to alleviate this problem, by identifying and removing systematic biases. We selected five different DM approaches, among the ones most often used on microarray data:

- **Combat** is a method specifically devised for removing batch effects in gene-expression data [35]. This method assumes the batches to be known, and that systematic variations follow an additive-multiplicative model

$$ {y}_{sjk}={\alpha}_j+\mathbf{X}{\boldsymbol{\beta}}_j+{\gamma}_{jk}+{\delta}_{jk}{\epsilon}_{sjk}^{\hbox{'}} $$

where *y*_*sjk*_ is the expression of gene *j* in sample *s* in batch *k*, *a*_*j*_ is the overall gene expression of *j*, ***X*** and ***β***_j_ are respectively the design matrix and the gene-specific coefficients vector, while the remaining terms are the additive and multiplicative batch effects, respectively. These effects are estimated through an approach that uses hyper-priors and pool information across all available probesets. We used the Combat implementation of the R package *sva* in all analyses.

- **RMA** (Robust Multi-array Average, [23]) is an algorithm for background correcting, normalizing and summarizing microarray data. The normalization phase is carried out with the Quantile Normalization method, that substitutes the expression value of each probe *t* with the average expression calculated over all probes that rank equally across all available profiles. In our experimentation we used the RMA function of the R package *affy*.
- **RMA-Combat.** We also include the hybrid solution RMA-Combat, consisting in a pipeline that first applies the RMA method and then Combat.
- **Surrogate Variable Analysis (SVA).** The SVA approach introduced by Leek and Storey [36] attempts to identify and remove all confounding factors negatively affecting the analysis, including eventual batch-effects. Similarly to Combat, this method explicitly takes in account the study design. In the common case–control scenario, the SVA model is the following: ${y}_{sj}={\alpha}_j+{\upbeta}_{\mathrm{j}}{\mathrm{x}}_{\mathrm{s}}+{\displaystyle {\sum}_{\mathrm{k}}}{\gamma}_{jk}{g}_{ks}+{\epsilon}_{sj}$, where *y*_*sj*_ is the expression of gene *j* in sample *s*, *a*_*j*_ is the overall gene expression of *j*, x_s_ is a binary variable indicating whether sample *s* is a case or a control, *β*_*j*_ represents the average difference in expression between the two conditions in gene *j*, and ϵ_*sj*_ is a random error. The term ${\displaystyle {\sum}_{\mathrm{k}}}{\gamma}_{jk}{g}_{ks}$ represents the cumulative effect on *y*_*sj*_ of *K* unknown confounding factors *g*_*ks*_, multiplied by their gene-specific coefficients *γ*_*jk*_. SVA attempts to estimate confounding factors’ global effect by deriving a set of surrogate variables ***h***_1_, ***h***_2_, …, ***h***_*K*_ whose span covers the same linear space spanned by the vectors ***g***_*k*_. These surrogate variables can then be used as covariates in all subsequent analysis in order to rule out the effect of the unknown confounding factors.
  To the best of our knowledge, no previous study applied SVA on gene-network reconstruction, and a detailed discussion about how to adapt SVA for this task is reported in the Additional file 2. Briefly, assuming that each TF_*i*_ has a significant effect only on a restricted subset of genes, all major systematic variations involving a large portion of transcripts should be due to experimental factors, batch-effects or confounding factors. Given this assumption, for the data collections used in this study the SVA model becomes: ${y}_{sj}={\alpha}_j+{\displaystyle {\sum}_{\mathrm{k}}}{\gamma}_{jk}{g}_{ks}+{\epsilon}_{sj}$. From a computational perspective this formulation implies that the surrogate variables are estimated by applying a Singular Value Decomposition to the expression matrix, after having centered each gene on its mean. The estimated surrogate variables are then used for computing the vectors ***C***_*t*,*SVA*_ and ***P***_*t*,*SVA*_. This means that *C*_*t*,*i*,*SVA*_ is a partial correlations [77], quantifying the linear association between the transcription factor *TF*_*t*_ and gene *i given* the information embedded within the surrogate variables.
- **Scaling** the expression values of each dataset so that all genes have the same mean and standard deviation is a further suitable approach. In particular, we scale the expression of each probeset in each dataset to zero mean and unitary standard deviation.
- **No-correction.** The naïve solution of pooling all data together without removing systematic variations is included in the analysis as well, in order to contrast the effectiveness of the other methods.

#### Baseline approaches

A relevant question is whether employing complicate statistical techniques in order to co-analyze several datasets actually provides any advantage with respect to analyze a single dataset in isolation. DM and MA methods heavily process the data, following assumptions that are not always satisfied. Consequently, these methods may induce biases rather than remove batch-effects. To answer this question we adopted a **Single-Dataset** approach, consisting in separately analyzing each dataset and then averaging the performance within each data collection. More in detail, let π_Π_^1^ … π_Π_^*m*^ be the performances obtained on datasets *D*_1_, …, *D*_*m*_ in collection ***M*** by using the metric Π. The Single-Dataset approach calculates a weighted performance ${\pi}_{\Pi}=\frac{{\displaystyle \sum }{s}_j\cdotp {\uppi}_{\Pi}^j}{{\displaystyle \sum }{s}_j}$, that can be interpreted as the result to be expected if a single dataset randomly chosen from the collection is analyzed.

Finally, we also include a **Random-Guessing** approach consisting in randomly sampling *C*_*t*,*i*_ from a uniform distribution. Theoretically, we expect this method to achieve the lowest performances among all other algorithms.

### Reconstruction of the Ikaros interaction network on PBMC data

Generalizing the results of this work to any network learning algorithm is out of the scope of this paper. However, we perform a proof-of-concept application in order to provide initial evidence that the results obtained in the context of relevance networks, arguably the simplest type of reverse engineering networks, are also valid when more complicated algorithms are used.

To this purpose, we analyze a set of Peripheral Blood Mononuclear Cells (PBMC) gene expression datasets extracted from GEO. We attempt to reconstruct the regulatory network of the Ikaros transcription factor by applying the SES (Statistically Equivalent Signatures) algorithm [78]. The predictions were validated against a list of experimentally determined Ikaros targets as retrieved from the literature [79, 80]. The IKZF1 gene encodes the transcription factor that belongs to the family of zinc-finger DNA proteins [81]. Ikaros displays crucial functions in the fetal and adult hemo-lymphopoietic system. It functions as a regulator of lymphocyte differentiation and its loss has been connected with the development of lymphoid leukemia.

The following sections describe in detail the used data and the analysis pipeline.

#### PBMC Compendium and Ikaros known regulatory relationships

We assembled a compendium of seven public microarray gene expression datasets of human PBMC. PBMC are the populations of blood cells having a round nucleus that constitute a pivotal part of the peripheral immune system. These include lymphocytes (T cells, B cells and NK cells), monocytes, macrophages, dendritic cells. Their abundance and the simplicity of their extraction (an intravenous injection is sufficient for collecting a sample) render them interesting candidate for scientific studies. Note that the selection of human microarray datasets serves for further testing the validity of our results in the spectrum of human subject studies.

For assembling this compendium, only studies comprising randomly-selected healthy-control subjects were taken in consideration. In particular, for each study only the control group was retained for our analysis. The idea is that control groups formed by randomly chosen healthy individuals can be considered as independent sampling from the same population, and are thus suitable for being analyzed through MA and DM methods. In total, the collection counts 181 expression profiles all measured with the Affymetrix Human Genome U133 Plus 2.0 Array (41245 probesets). The expression of Ikaros is measured by nine of these probesets. We used in turn each of these probesets and we merged together their respective networks.

Finally, a list ***I***_*Ikaros*_ of Ikaros regulatory relationships was built from literature information and computational analyses. Particularly, we built a list ***I***_*Ikaros*_ containing 2658 unique interactions by merging together 2497 Ikaros targets identified through Chip-seq and microarray analysis [79] along with 137, 115, 133 and 154 Ikaros-gene interactions found in CD43- (young mature B-cells) CD19+ (mature B-cells), T-naïve and T-reg cells, respectively. These latter lists were derived from the analysis of DNAse-seq data from the ENCODE project [80], following the approach presented in [82]. Briefly, DNase hyper-sensitive regions (DHS) were identified using Hotspot v4 [83], and DHS peaks were subsequently scanned for footprints of DNA-binding proteins by the Wellington algorithm using pyDNase [84]. Transcription start sites (TSS) were obtained from the University of California, Santa Cruz (UCSC) Genes Track, and the region flanking 5Kb upstream to 5Kb downstream of the TSS was defined as the promoter region. The footprints within the promoters were subsequently scanned for identifying binding motifs specific for 483 transcription factors, using the TRANSFAC database [85] and the Match algorithm [86]. Genes whose promoter contained a motif instance were considered as potential regulatory targets. This allowed identifying (a) candidate regulators and (b) candidate targets for each TF, including Ikaros.

#### Deconvolution of PBMC and outlier identification

The presence of different cell-types in the PBMC samples implies that expression values are averaged over a mixture of different distributions. Subjects included in each study may have significantly different cell proportions, and this in turn may generate correlations among probesets that do not reflect any underlying gene-gene interaction [4]. In order to avoid this scenario, we estimate the cell-proportions for each sample through a deconvolution approach and then we eliminate subjects that appear to be outliers and that may prejudice the analysis. We use the deconvolution method introduced by Abbas and co-authors [87] and implemented in the CellMix R package [88]. This approach uses a fixed set of expression signatures characterizing the expression profiles of seventeen different cell types in order to estimate the proportion of these cell types in the PBMC data. The multivariate outlier detection was conducted by using the PCout [89] algorithm from the “mvoutlier” R package [90]. This algorithm utilizes simple properties of principal components and is particularly effective in high-dimensional data.

#### SES algorithm

The SES algorithm [78] as implemented in the ‘MXM’ R package was used in order to reconstruct Ikaros regulatory network. The SES algorithm attempts to identify highly predictive signatures for a given target. In this context, a gene expression signature consists of the minimal set of gene expression measurements that is necessary in order to predict the value of Ikaros. As demonstrated in [91], the signature of a target corresponds, under broadly accepted assumptions, to the variables that are adjacent to the target in the Bayesian Network representing the data distribution at hand. Consequently, these gene expression signatures also correspond to the set of potential regulators/targets of Ikaros in the context of the available measurements. Lack of statistical power may make two or more signatures statistically indistinguishable. The SES algorithm is specifically devised in order to cope with this problem and to attempt to retrieve statistically equivalent signatures.

SES belongs to the class of constraint-based, Bayesian Network reconstruction algorithms [92]. While relevance networks assess the presence of gene-gene interactions through simple pairwise correlations, constraint-based algorithms use tests of conditional independence in order to find variables that are associated to the target given any subset of other measurements. This implies that SES should return only genes whose association with Ikaros is not mediated by any other measured gene. In contrast, relevance network cannot distinguish among direct and indirect associations.

SES requires the user to set a priori two hyper-parameters, a threshold for assessing p-values significance and the size of the maximum conditioning set. In our analyses these hyper-parameters were set to 0.01 and 5, respectively. The signatures found on single probesets were merged together, as well as the results retrieved on the nine different probesets measuring Ikaros.

#### Network reconstruction and validation

Based on our previous findings, we picked the Combat and Fixed-Effects methods as representatives for the DM and MA approaches, respectively. We also used the No-Correction and Single-Dataset approaches in order to characterize the scenarios where batch-effects are ignored or a randomly chosen dataset of the PBMC collection is analyzed in isolation. For the Combat and No-Correction approaches the deconvolution and outlier deletion steps were performed on their respective merged datasets, while for the Fixed-Effects and Single-Datasets methods the two pre-processing steps were performed independently for each study of the PBMC collection.

Network reconstruction performances were measured in terms of precision, recall and odds ratio. Let ***Q***_*Ikaros*,*X*_ be the list of Ikaros interactions retrieved using SES couple with the MA or DM method *X*, and ¬ ***I***_*Ikaros*_ the list of genes that are not part of Ikaros regulatory network (|***I***_*Ikaros*_ ∪ ¬ ***I***_*Ikaros*_| = *n*). Precision is defined as $PRE{C}_X=\frac{\left|{\boldsymbol{Q}}_{Ikaros}\cap {\boldsymbol{I}}_{ikaros}\right|}{{\boldsymbol{Q}}_{Ikaros}}$, and indicates the proportion of actual interactions that are present in the retrieved signature. Recall (or sensitivity) is computed as $RECAL{L}_X=\frac{\left|{\boldsymbol{Q}}_{Ikaros}\cap {\boldsymbol{I}}_{ikaros}\right|}{{\boldsymbol{I}}_{Ikaros}}$, that is the proportion of genes that are in the Ikaros regulatory program and are classified as such.

The odds ratio quantifies the likelihood that a given proportion of regulatory relationships is retrieved by chance, and is computed as $OddsRati{o}_X=\frac{PRE{C}_X}{PRE{C}_{Trivial}}$, where $PRE{C}_{Trivial}=\frac{{\boldsymbol{I}}_{Ikaros}}{n}$ represents the sensitivity achievable by classifying all *n* genes as belonging to the Ikaros regulatory program. An odds ratio of one indicates performances that are indistinguishable from random guessing, and we used a hypergeometric test [93] in order to assess the null hypothesis *H*_0_ : *OddsRatio*_*X*_ = 1.

## Results

### E. coli and Yeast compendia

**Fig. 2:**
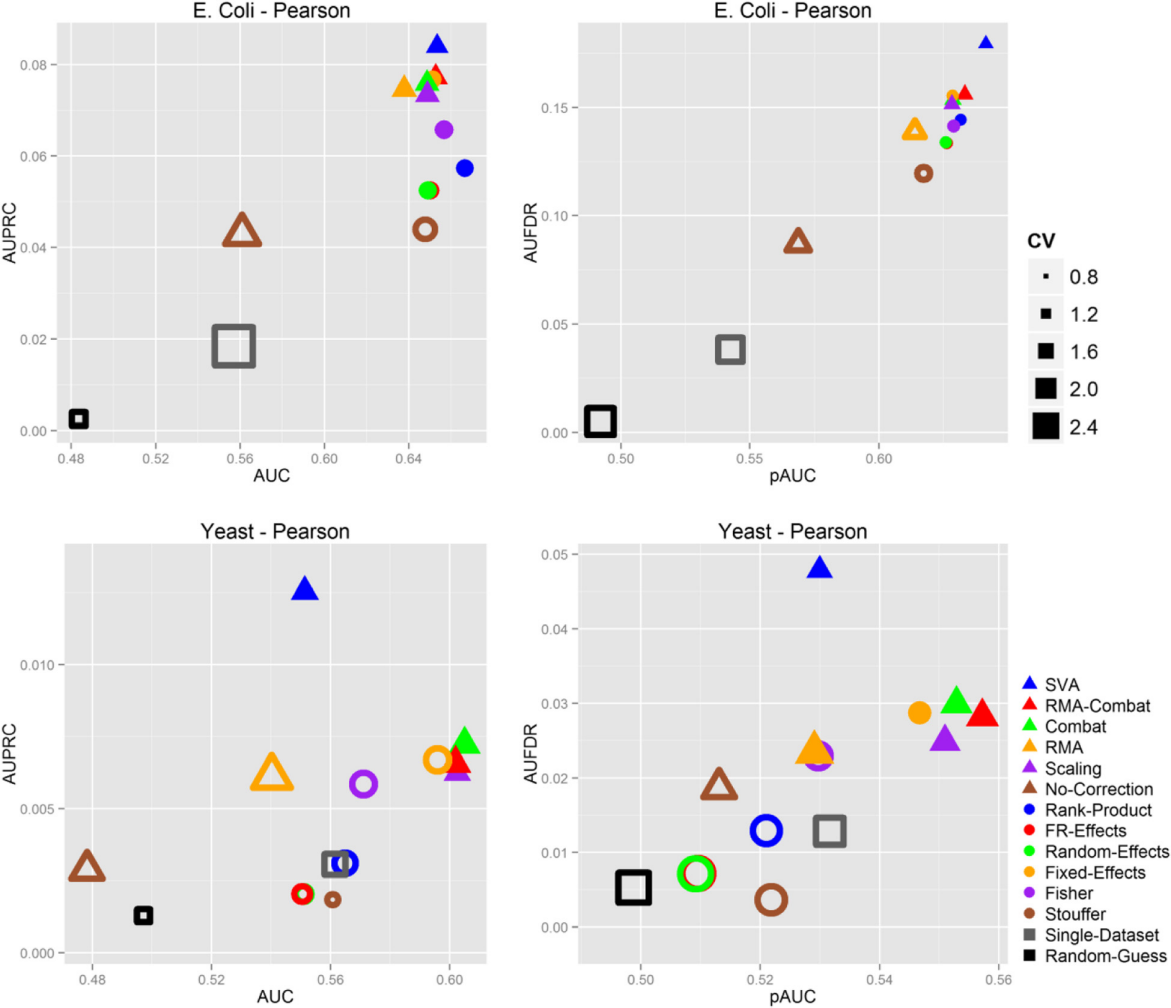
Results of the experimentations on E. coli and Yeast compendia using the Spearman correlation. Panels on the left side report global performance metrics (x-axis: AUC, y-axis: AUPRC), while panels on the right report partial performance information (x-axis: pAUC, y-axis: AUFDR). Results in the top row are computed on the E. coli dataset compendium, while results on the Yeast dataset collection are reported in the other two panels. MA, DM and baseline methods are indicated with circular, triangular and square markers, respectively. Non-filled markers indicate methods that are statistically significantly different with respect to the best performing ones in both metrics (*p*-value < 0.05, one-tailed paired *t*-test). The size of each marker is directly proportional to the Coefficient of Variation (CV) between its respective metrics

Figure 2 and Additional file 1: Tables S4 – S5 report the results on the E. coli and Yeast compendia computed using the Pearson correlation. Results based on Spearman correlation follow similar patterns and are reported in the (Additional file 1: Figure S1). Panels in the top row present the results obtained on the E. coli compendium, while findings on the Yeast collection are summarized in the other two subplots. Each panel reports two different performance metrics. The panels on the left side summarize global performance metrics, having the AUC on the x-axis and the AUPRC on the y-axis. Subplots on the right side report partial performances, with the pAUC on the x-axis and the AUFDR on the y-axis. MA, DM and baseline methods correspond to circular, triangular and square markers, respectively. In each panel, the size of each marker is directly proportional to average between the Coefficients of Variation (CV) computed on the x and y-axis metric. The CV is a convenient way for representing variability with respect to the order of magnitude of the measurements, and is computed as the ratio between standard deviation and average value. Non-filled markers indicate methods that are statistically significantly different from both methods that perform best in the two metrics (*p*-value < 0.05, one-tailed paired *t*-test).

All four panels present a similar picture, with several DM and MA methods clustering together and achieving comparable performances, while the Random-Guess, Single-Dataset and No-Correction approaches usually providing significantly worst results. Best performing methods usually present a variability that is smaller than the one of the outperformed methods.

*All in all, the results show that systematic biases across studies must be taken into account for retrieving gene-gene interactions, and that both MA and DM approaches are effective in dealing with such systematic variations.*

Retrieving gene-gene interactions in the Yeast dataset collection have proven to be harder than in E. coli. Performances were generally poorer, with AUC and pAUC values up to 5 point inferior than the corresponding performances in the E. coli compendium, and both AUPRC and AUFDR ranging far below 0.05.

**Fig. 3:**
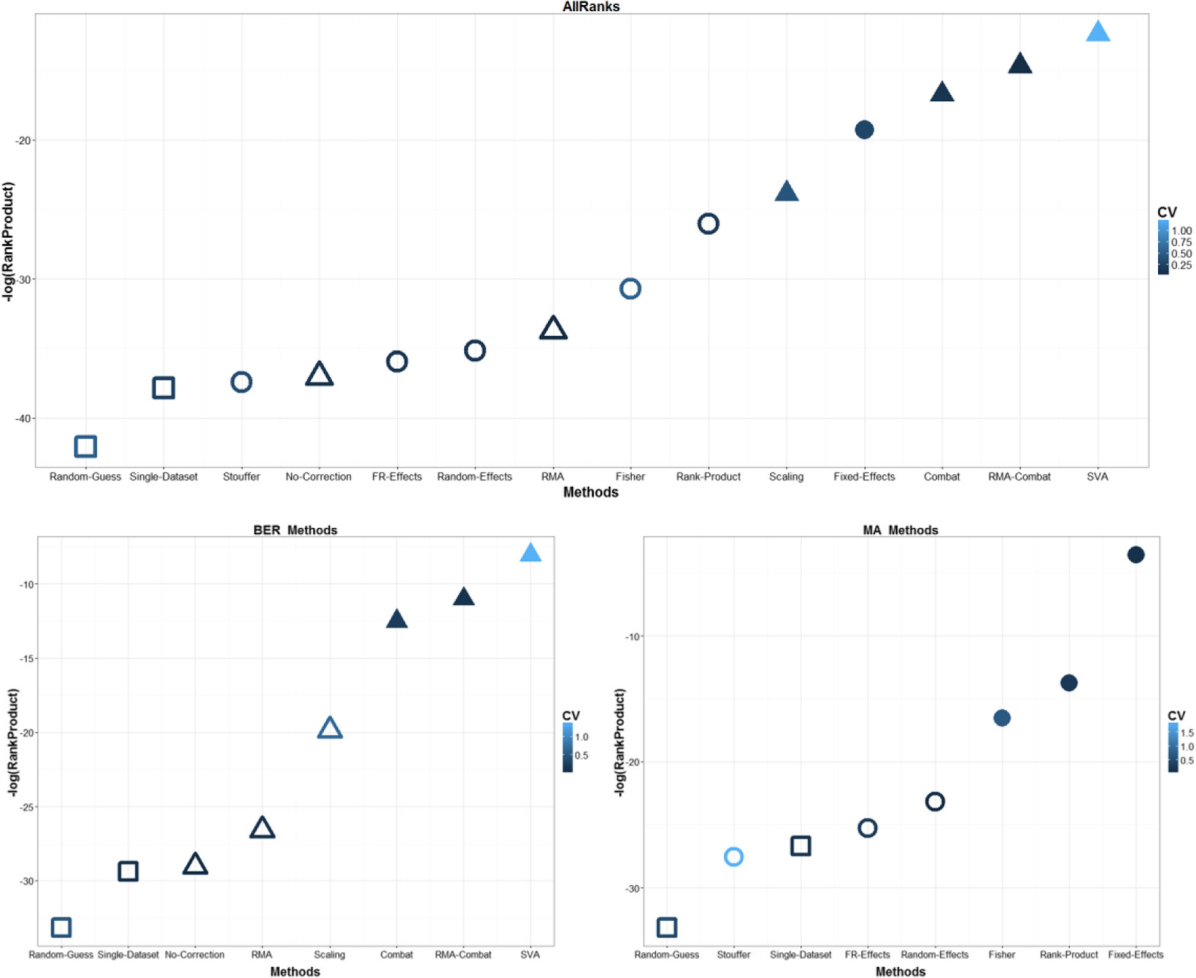
Rank-product analysis of MA and BER methods. Methods are ranked according to their performances, separately for each combination of data compendium (E. coli and Yeast), correlation measure (Pearson and Spearman) and performance metric (AUC, pAUC, AUPRC, AUFDR), for a total of 16 different ranks. These ranks are then combined using the Rank-Product method, and the statistical significance of the ranks are evaluated with the method reported in [75]. The negative logarithm of the Rank-Product score is reported on the y-axis, while methods are listed on the x-axis. Triangular markers indicate BER methods, round markers MA methods, square markers baseline approaches. The color of each marker is directly proportional to the Coefficient of Variation (CV) of the respective log-transformed rank-product score (lighter color corresponds to higher variability). Methods that tend to be consistently ranked in the top positions are placed on the top-right of the plots, while poorly performing methods remain the in the bottom-left corner. The plot on the top report the global, final rank of both MA and BER methods, while the two plots on the bottom focus on BER and MA methods, respectively

Results are further summarized in Fig. 3 through a Rank-Product analysis. The combination of both E. coli and Yeast compendia with the two correlation measures and the four different metrics provides a total of 16 different ways to rank MA and DM methods according to their performances. These sixteen ranks are synthesized with the Rank-Product method and the final results are reported in the top panel of Fig. 3. All methods are listed on the x-axis, ordered from left to right according to log-transformed Rank-Product score (reported on the y-axis). Higher scores characterize methods that consistently achieve the top positions across all ranks. Rank statistical significance is assessed with the methods reported in [75], and *p*-values < 0.05 are indicated with filled markers. The coefficient of variability for each method determines the color of the corresponding marker, with lighter color corresponding to higher CV.

The SVA, Combat, RMA-Combat, Fixed-Effect and Scaling methods are confirmed as the best performing methods, occupying the first position in the Rank-Product analysis. SVA shows a relatively high variance, indicating that sometimes it fails in reaching the top positions in terms of performances. The Random-Guess approach is stable in last position, followed by the Single-Dataset, Stouffer and No-Correction methods. The two bottom panels in Fig. 3 restrict the Rank-Product analysis to the DM and MA methods, respectively. The SVA, RMA-Combat and Combat method should be the methods of choice within the DM approaches, while Fixed-Effects, Rank-Product and Fisher excel among the MA methods.

Similar figures restricting the Rank-Product analysis to Global and Local performances only, as well as Pearson and Spearman correlations and E. coli versus Yeast are available in the Additional file 1. The conclusions that can be drawn from these figures are in close agreement to the ones discussed until now.

**Fig. 4:**
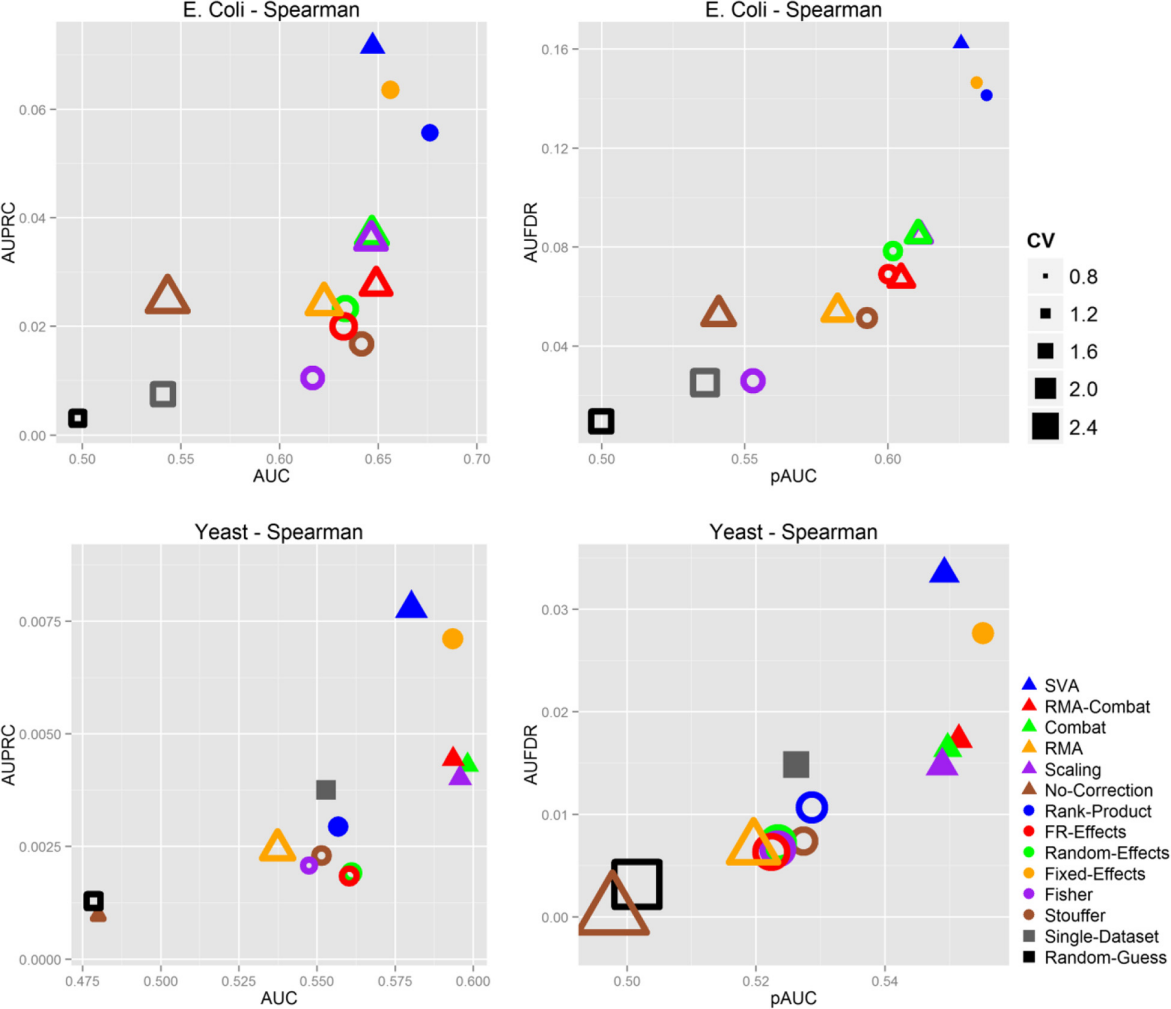
Results of the experimentations on E. coli and Yeast compendia using the Spearman correlation p-values. Details as in Fig. 2. Methods generally achieve lower performances when p-values are used instead of correlations for ranking candidate gene-gene interactions. This is mainly due to the prevalence of close-to-zero p-values that create ties negatively affecting the performance metrics

Figure 4 reports the performances computed using the vector of *p*-values ***P***_*t*,*X*_ instead of the correlation values ***C***_*t*,*X*_. In E. coli there is a dramatic worsening in performances for most of the methods. A decrease in performances can also be observed for the Yeast compendium, although to a lesser extent. A possible explanation for these patterns is the presence of several high-significant correlations, whose corresponding *p*-values are exactly zero or too low to be distinguished at machine precision. These zero *p*-values create ties that severely affect the ranking of the candidate interactions and consequently the evaluation of the performances.

A close inspection of the results seems to confirm this hypothesis. Table 2 reveals that methods showing a large performance decrease in E. coli between the ***C***_*t*,*X*_ and ***P***_*t*,*X*_ -based results have a large percentage of *p*-values that are exactly zero. SVA, Rank-Product and Fixed-Effects methods do not produce zero p-values, and they do not suffer any performance loss. However, Random-Effects and FR-Effects do not produce zero p-values as well, and they still achieve worse performances when ***P***_*t*,*X*_ is used instead of ***C***_*t*,*X*_. The answer to this issue lays in the fact that there is not a bijective correspondence between ***C***_*t*,*X*_ and ***P***_*t*,*X*_ for the Random-Effects methods, and consequently neither for the FR-Effects one. In other words, if *C*_*t*,*i*_ > *C*_*t*,*j*_ holds, then *P*_*t*,*i*_ < *P*_*t*,*j*_ holds as well if the correlations are computed with the Fixed-Effects model, but not if they are computed with the Random-Effects method. The statistical significance of correlation in the Random-Effects approach depends on the estimation of the between-study variance $\widehat{\tau}$, and this variance is separately estimated for each correlation. Consequently, candidate interactions are ranked differently by the Random-Effect model depending whether correlations or p-values are used, and the results seem to indicate that the ranking provided by the correlation values better reflects the actual underlying gene-gene interactions.

**Table 2:** Proportion of p-values being exactly zero for E. coli and Yeast, Pearson correlation results

	E. coli	Yeast
Rank-Product	0 %	0 %
FR-Effects	0 %	0 %
Random-Effects	0 %	0 %
Fixed-Effects	0 %	0 %
Fisher	32.5 %	19.1 %
Stouffer	9.5 %	11.7 %
SVA	0 %	0 %
RMA-Combat	9.0 %	2.5 %
Combat	9.4 %	2.8 %
Scaling	9.1 %	2.7 %
RMA	15.6 %	13.4 %
No-Correction	85.6 %	98.7 %
Random-Guess	0 %	0 %

The majority of DM methods assigns a zero *p*-value to some percentage of the predictions, while only the Fisher and Stouffer MA methods do so. These percentages are higher in E. coli than in Yeast, suggesting that in the first compendium the statistical associations are stronger or more detectable due to higher statistical power

### Synthetic data

**Fig. 5:**
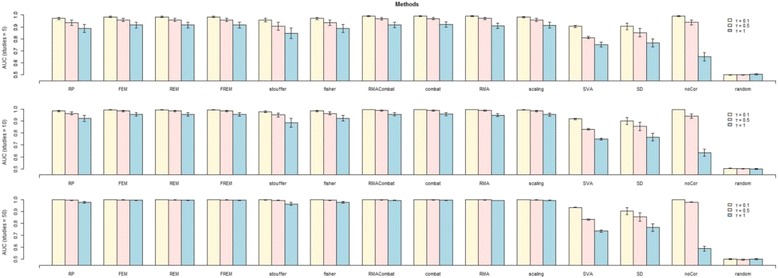
AUC results on simulated data for different number of studies using Pearson Correlations. Each row reports the results obtained on the data collections including 5, 10 and 50 studies, respectively. For each row the performances of each method are reported for level of systematic bias *τ* equal to 0.1, 0.5 and 1. All results are averaged over five different synthetic networks and different sample sizes (5, 20 and 50 samples). Standard deviations are indicated by the whiskers at the top of each plot. SD stands for Single-Dataset, while FEM, REM and FREM stand for Fixed, Random and FR-Effects method, respectively

**Fig. 6:**
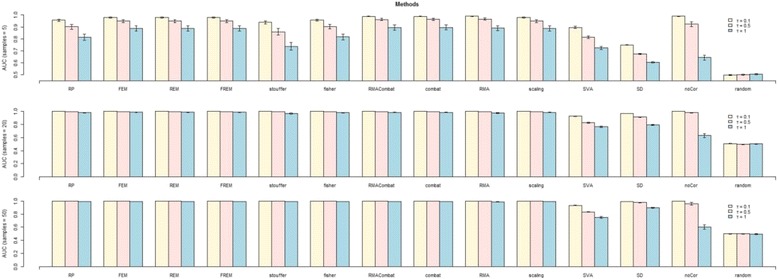
AUC results on simulated data across different sample sizes using Pearson Correlations. Each row reports the results obtained for a given sample size (5, 20 and 50 samples in each study, respectively). For each row the performances of each method are reported for level of systematic bias *τ* equal to 0.1, 0.5 and 1. All results are averaged over five different synthetic networks and different numbers of studies included in each collection (5, 10 and 50 studies). Standard deviations are indicated by the whiskers at the top of each plot. SD stands for Single-Dataset, while FEM, REM and FREM stand for Fixed, Random and FR-Effects method, respectively

The results on simulated data for the AUC metric are reported in Figs. 5 and 6. Results on other metrics follow similar patterns, and the respective Figures are reported in the Additional file 1. The numerical results for all simulated scenarios are in Additional files 3 and 4. As expected, results improve for increasing number of studies or samples, while larger level of systematic bias corresponds to worse performances. The Single-Dataset approach is systematically outperformed by MA or DM methods in all scenarios. The No-Correction approach also achieves poor performances for high level of batch-effects, even though it is quite competitive for mild systematic biases. *AUC* ≈ 0.5 for the Random-Guess approach in all cases. The remaining MA and DM methods achieve comparable performances, both in terms of average performance and respective variance. SVA seems to be an exception, thought, achieving quite lower performances. Quite surprisingly, SVA performances drop significantly with the maximum total sample size, i.e., when 50 studies with 50 samples each are analyzed (2500 total sample size). Concomitantly, the number of surrogate variables estimated in these setting is ~60, versus ~5-10 when the total sample size is lower. We argue that such an elevated number of surrogate variables negatively affects the computation of conditional correlations, leading to a worsening in performances.

Also for the synthetic data results computed using the p-value vectors ***P***_*t*,*X*_ show a decrease in performance (Additional files 3 and 4). Particularly, across all simulation scenarios, correlation functions and performance metrics results based on correlations outperform the corresponding results based on p-values 52 % of the times. The average difference in performance varies depending on the metric: 0.001 for AUC, 0.12 for AUPRC, 0.01 for pAUC and 0.1 for AUFDR. Interestingly, this effect becomes more marked with increasing sample size and decreasing systematic bias (Additional file 1: Figures S10 – S41), confirming that the performance loss is due to an excess of statistical power that generate zero or close to zero *p*-values.

### Reconstruction of the Ikaros interaction network on PBMC data

Table 3 summarizes the results of the reconstruction of the Ikaros regulatory program on PBMC data. Combat achieved the best performances, followed by the Fixed-Effect method, the Single-Dataset approach, and No-Correction. All methods achieved odds ratio statistically significantly different from one at the 0.05 level. For the Single-Dataset approach, the results actually varied depending on the specific study, ranging from highly significant (*p*-value <0.0001) to random guessing (*p*-value: 0.66). We correlated the odd ratios and *p*-values achieved on each dataset with the sample size, and interestingly no association was detected (correlation *p*-value > 0.25).

**Table 3:** Reconstruction of Ikaros regulatory program in PBMC data collection. For each method the number of predicted and correctly retrieved interactions is reported, along with the odds ratio, precision and recall performances (see text for further details on these metrics)

Method	# Predicted interactions	# Retrieved interactions	Odds ratio	Odds ratio significance	Precision	Recall
Combat	82	21	2.2726	0.00022	0.2561	0.0093
Fixed-Effect	102	21	1.827	0.00440	0.2059	0.0093
Single-Dataset	387	70	1.6513	[<0.0001 - 0.65914]	0.1861	0.0310
No-Correction	113	21	1.6491	0.01435	0.185841	0.0092

Odds ratio statistical significance is assessed through the hypergeometric test. For the Single-Dataset approach distinct performances and significance *p*-values were computed for each dataset, summarized here as a weighted average of the performance and with the interval spanned by the *p*-values, respectively

**Fig. 7:**
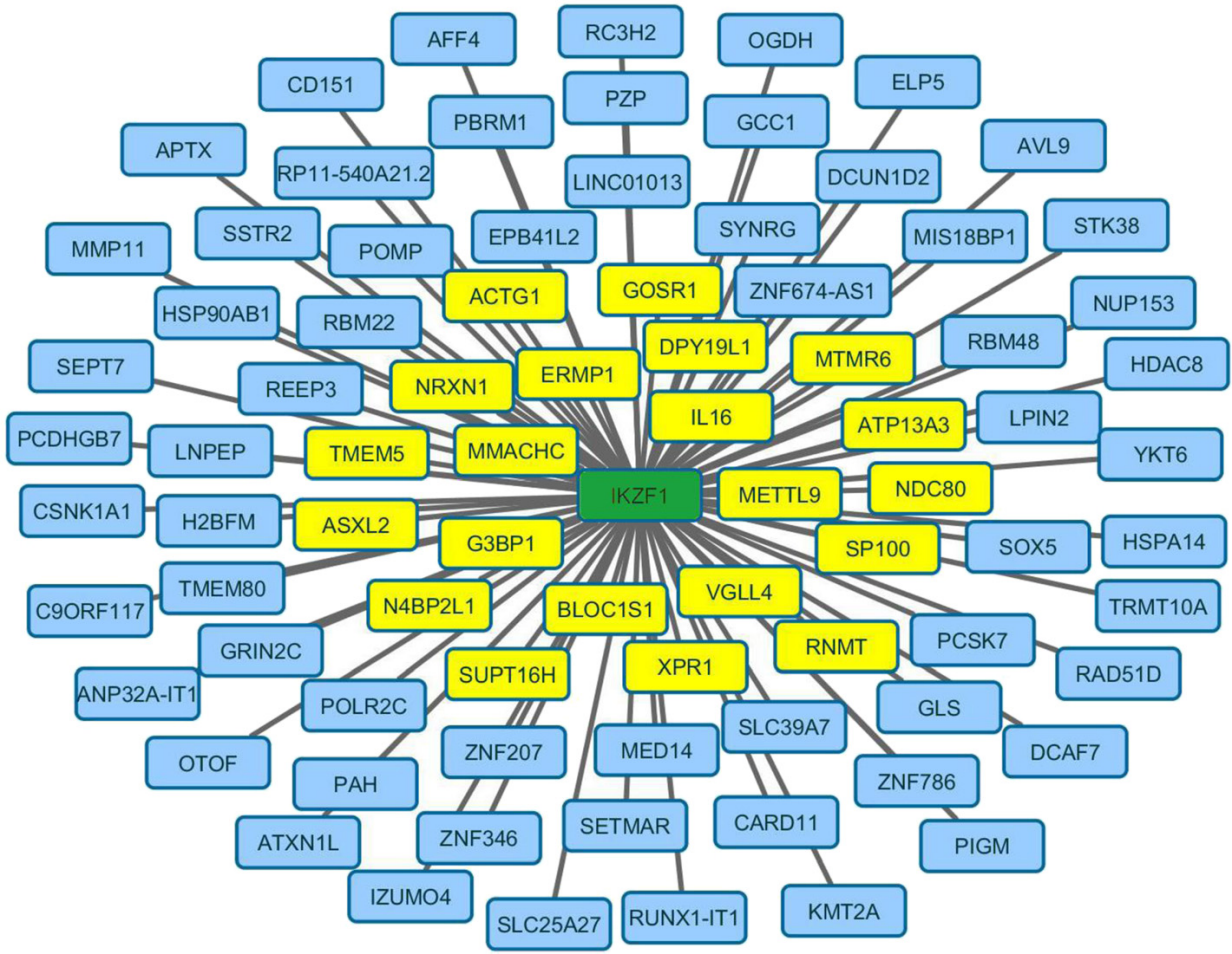
Ikaros regulatory program as reconstructed by applying the SES and Combat algorithms on PBMC data. Correctly retrieved interactions are marked in yellow

Figure 7 reports the Ikaros regulatory program reconstructed on the PBMC data using SES coupled with Combat. Yellow nodes indicated genes included in ***I***_*Ikaros*_.

## Discussion

In the present work we have compared two different approaches, Data-Merging and Meta-Analysis, on the reconstruction of relevance networks in collection of microarray, gene-expression data. The comparison has been performed on two compendia of studies retrieved from the literature, on Escherichia coli and Yeast, respectively. Further analyses on simulated data have been used for strengthening and deepening the conclusion of the comparison. Finally, a contrived case-study on human PBMC data have been presented for showing how the results of this study might transfer on more sophisticated network reconstruction approaches.

The results on both simulated and real data provide coherent conclusions, which can be summarized in the following points:

1. *Batch-effects must be carefully taken into consideration for retrieving gene-gene interactions from microarray data.* The naïve solution of ignoring systematic biases (No-Correction approach) was outperformed by the other methods in all experimentations. This result supports our claim that batch-effects can hide actual dependencies between the measured quantities or create spurious associations between elements that are not functionally related.
2. *DM and MA methods are equally effective in contrasting batch-effects.* According to the results it is not possible to state that one approach is universally better than the other one. However, within their respective approaches, and acknowledging that the results vary across the performed experimentations, the SVA/Combat/RMA-Combat and the Fixed-Effects methods have usually achieved the best performances. In contrast, the Single-Dataset method usually provides poorer results, supporting the hypothesis that integratively analyzing multiple datasets leads to improved and more robust findings.
3. *Correlation statistics should be preferred to p-values in ranking associations.* Performances have proven to drastically change depending on whether they are computed on correlations or p-values. We have observed that this effect is mainly due to ties generated by zero or close to zero *p*-values.

This study presents a number of limitations that should be carefully considered when implementing the recommendations above. First, within-study batch-effects were only partially addressed, by pre-processing each single dataset with RMA. While the Quantile Normalization step included in the RMA algorithm should have removed at least part of the within-study biases, it is known that this approach is not optimal [5]. This is also demonstrated by our results, where the RMA method never achieved the best performances. Secondly, the design of the comparison slightly advantages DM method, particularly because all datasets belong to the same data collection and thus measure the same probesets. When this is not the case (e.g., when data from different microarray platforms are co-analyzed), DM method are not easily applicable, while MA methods can be straightforwardly used. Finally, we also notice that in our experimentations we did not explore joint uses of correlations and *p*-values for ranking gene-gene interactions. A possible practice is to filter the candidate interactions by using the *p*-values and then raking the most significant gene-pairs according to their correlation values.

The SVA method merits a separate note. To the best of our knowledge, this is the first study employing this methodology in the context of retrieving gene-gene interactions. Adapting SVA for this task has required a dedicated sub-study, reported and commented in the Additional file 2. Despite the excellent performances obtained on the real data, we notice that this method performed quite poorly on the synthetic data. This drop in performances is particular evident for large samples sizes. A possible explanation might be the inclusion of several irrelevant surrogate variables when large datasets are analyzed: out of 60 surrogate variables produced when 2500 samples are available in the merged dataset, only 3 explain more than 1 % of variance. These noisy variables might in turn make the estimation of partial correlations and respective *p*-values quite inaccurate. Further studies are needed in order to better investigate this phenomenon.

Future work will also focus on the generalization of the present results towards more sophisticated network reconstruction algorithms, particularly Bayesian and Causal Networks [94]. We already presented a first, contrived case-study where we have reconstructed (part of) the regulatory network of the Ikaros transcription factor from human PBMC data. This case-study presented several characteristics that made it harder to solve than the reconstruction of the E. coli and Yeast regulatory networks: different cell-type proportions across subjects, a many-to-many correspondence between genes and probesets, the list of known interactions was partially derived from animal models instead than human data. Moreover, we used a constraint-based network reconstruction algorithm instead of relevance networks. Despite all these difference both Combat and Fixed-Effects method demonstrated to be able to retrieve subsets of genes significantly enriched for known Ikaros interactions and to outperform both the No-Correction and Single-Dataset approach, as expected from the results of the comparison presented in this study.

## Conclusions

Batch-effects should be carefully taken into account when retrieving gene-gene interactions, and researchers can adopt either a DM or MA approach depending on the specific application at hand. Correlation statistics should be preferred over *p*-values for assessing and comparing the strength of associations, especially for large sample sizes.

## Availability of supporting data

The data sets use in this article are available from their respective repositories. See Tables S1 to S3 in the Additional file 1 for the appropriate references.

Code for replicating the analysis is available at http://www.mensxmachina.org/.

## Additional files

Additional file 1: The Supplementary Material provides additional data and results supporting the conclusions of the study, including detailed descriptions of the E. coli and Yeast data compendia as well as all results produced on these compendia. (DOCX 4691 kb)

Additional file 2: Using Surrogate Variable Analysis for Network Reconstruction. This additional file presents a sub-study investigating modifications of the SVA model that allow to use the SVA method on network reconstruction tasks. (DOCX 137 kb)

Additional file 3: Simulations Results based on correlations. The Simulation Results table presents the results obtained on the synthetic data by using correlations as measure of association. (XLSX 1787 kb)

Additional file 4: Simulations Results based on p-values. The Simulation Results table presents the results obtained on the synthetic data by using the correlation p-values as measure of associations. (XLSX 1885 kb)
