# Erratum to: A comparative evaluation of data-merging and meta-analysis methods for reconstructing gene-gene interactions

**DOI:** 10.1186/s12859-016-1153-z

**Published:** 2016-07-27

**Authors:** Vincenzo Lagani, Argyro D. Karozou, David Gomez-Cabrero, Gilad Silberberg, Ioannis Tsamardinos

**Affiliations:** 1 Institute of Computer Science, Foundation for Research and Technology – Hellas, Heraklion, Greece; 2 Unit of Computational Medicine, Department of Medicine, Karolinska Institutet, 171 77 Stockholm, Sweden; 3 Center for Molecular Medicine, Karolinska Institutet, 171 77 Stockholm, Sweden; 4 Computer Science Department, University of Crete, Heraklion, Greece; 5 Unit of Clinical Epidemiology, Department of Medicine, Karolinska University Hospital, L8, 17176 Stockholm, Sweden; 6 Science for Life Laboratory, 17121 Solna, Sweden

## Erratum

In the publication of this article [1] two of the affiliations were reported incorrectly.

The error:

^4^ Computer Science Department, University of Crete, Heraklion, Sweden.

^5^ Unit of Clinical Epidemiology, Department of Medicine, Karolinska University Hospital, L8, 17176 Heraklion, Sweden.

Should instead read:

^4^ Computer Science Department, University of Crete, Heraklion, Greece.

^5^ Unit of Clinical Epidemiology, Department of Medicine, Karolinska University Hospital, L8, 17176 Stockholm, Sweden.

The correct affiliations are included in the author details of this erratum.
