# Supplementary material for: A comparative evaluation of data-merging and meta-analysis methods for reconstructing gene-gene interactions
Source: BMC Bioinformatics. 2016 Jun 6;17(Suppl 5):194. doi: 10.1186/s12859-016-1038-1 (PMC4905611; doi:10.1186/s12859-016-1038-1)
Supplement: Supplementary file 1 — The Supplementary Material provides additional data and results supporting the conclusions of the study, including detailed descriptions of the E. coli and Yeast data compendia as well as all results produced on these compendia. (DOCX 4691 kb) [file 12859_2016_1038_MOESM1_ESM.docx]

Supplementary Material for the manuscript “A comparative evaluation of data-merging and meta-analysis methods for reconstructing gene-gene interactions”

# Studies included in the E. Coli, Yeast and PBMC compendia

Table S 1: characteristics of the studies included in the Escherichia Coli dataset collection. Each study can be retrieved in the Gene Expression Omnibus (GEO) through its reference. For each dataset the total number of samples is reported, as well as the number of samples included in our comparison and a short description of the data.

| GEO reference | Number of Samples | Number of relevant sample | Notes |
| --- | --- | --- | --- |
| GSE4778 | 20 | 20 | Aerobically Grown Escherichia coli K-12 |
| GSE6425 | 44 | 44 | Time courses of E. coli MG1655 and UTI89 in vitro |
| GSE6836 | 266 | 266 | Compendium of Expression Profiles |
| GSE7326 | 24 | 24 | Cell death programmed by a restriction-modification system in E. coli |
| GSE7398 | 24 | 8 | four phases of growth in Fis KO and WT (Fis+) strains |
| GSE10158 | 43 | 43 | cefsulodin and mecillinam, alone and in combination |
| GSE11183 | 26 | 26 | Identification of HU regulon |
| GSE12411 | 28 | 28 | Time course data induced with differential amounts of IPTG |
| GSE17505 | 30 | 30 | High-Cell-Density Recombinant and Wild-Type E. coli |
| GSE21869 | 33 | 33 | Evolved clones isolated at various stages during adaptive experimental evolution |
| GSE33147 | 96 | 96 | Laboratory evolution on lactate or glycerol |

Table S 2: characteristics of the studies included in the Yeast dataset collection. Each study can be retrieved in the Gene Expression Omnibus (GEO) through its reference. For each dataset the total number of samples is reported, as well as the number of samples included in our comparison and a short description of the data.

| GEO reference | Number of Samples | Number of relevant sample | Notes |
| --- | --- | --- | --- |
| GSE1311 | 21 | 21 | Yeast desiccation / rehydration time course |
| GSE1312 | 21 | 21 | Yeast desiccation / rehydration time course |
| GSE1313 | 24 | 24 | Yeast desiccation / rehydration time course |
| GSE3076 | 96 | 96 | Budding Yeast |
| GSE15254 | 72 | 72 | yeast nitrogen assimilation |
| GSE18121 | 42 | 42 | Heat stress in different yeast strains |
| GSE25582 | 151 | 151 | Time course expression data |

Table S 3: characteristics of the studies included in the Peripheral Blood Mononuclear Cell (PBMC) dataset collection. Each study can be retrieved in the Gene Expression Omnibus (GEO) through its reference. For each dataset the total number of samples is reported, as well as the number of samples included in our comparison and a short description of the data.

| GEO reference | Number of Samples | Number of relevant sample | Notes |
| --- | --- | --- | --- |
| GSE46480 | 196 | 98 | Healthy individuals rapidly transported to high altitude |
| GSE27034 | 37 | 18 | Arterial Disease |
| GSE21942 | 29 | 15 | Multiple Sclerosis |
| GSE37567 | 102 | 7 | - |
| GSE17114 | 29 | 14 | Behçet’s disease. |
| GSE22224 | 46 | 9 | Kidney Recipients |
| GSE22255 | 40 | 20 | Ischemic Stroke |

# Supplementary results on the E. Coli and Yeast compendia


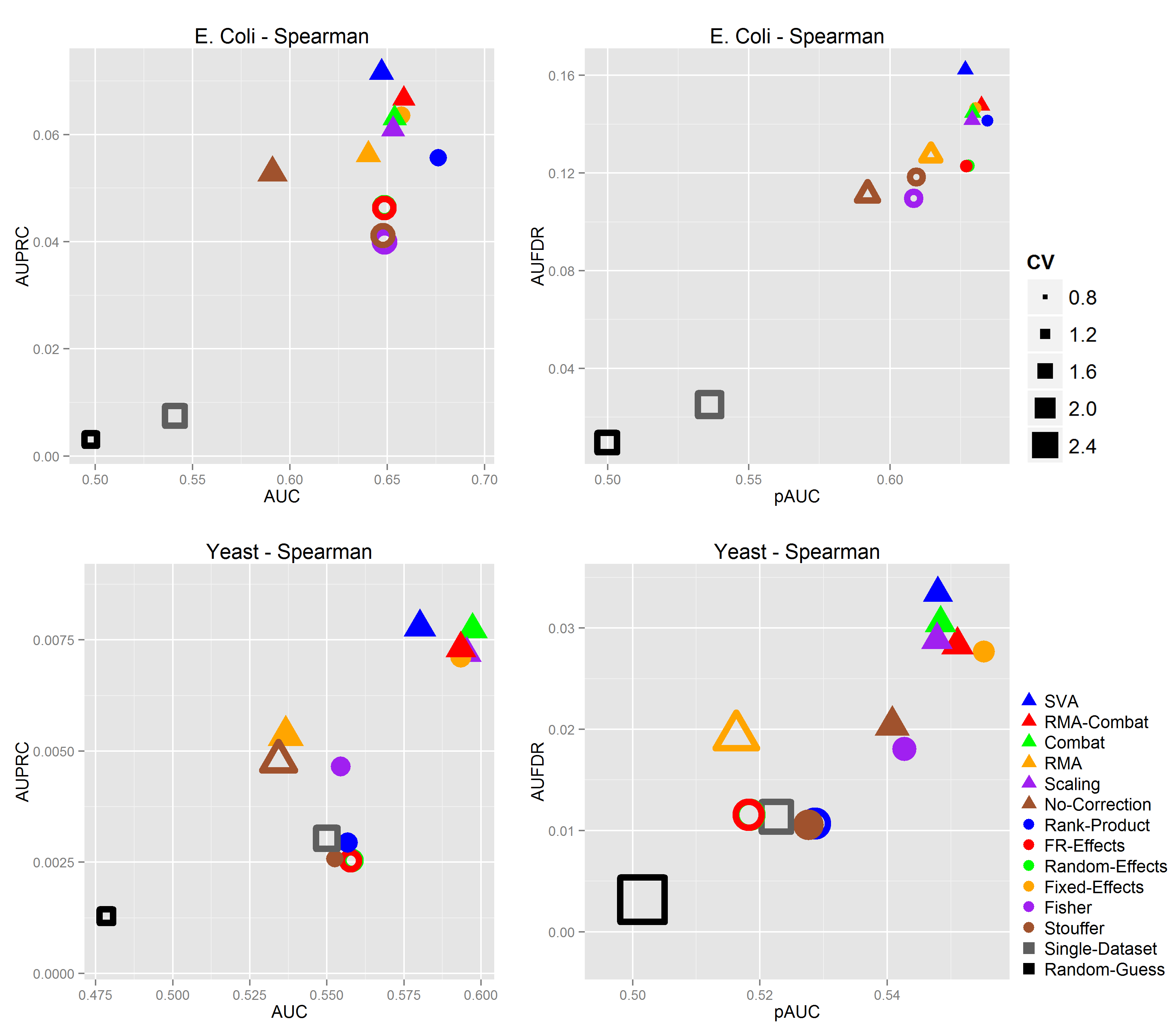


Figure S 1: results of the experimentations on E.Coli and Yeast compendia using the Spearman correlation. Panels on the left side report global performance metrics (x-axis: AUC, y-axis: AUPRC), while panels on the right report partial performance information (x-axis: pAUC, y-axis: AUFDR). Results in the top row are computed on the E. Coli dataset compendium, while results on the Yeast dataset collection are reported in the other two panels. MA, DM and baseline methods are indicated with square, triangular and circular markers, respectively. Non-filled markers indicate methods that are statistically significantly different with respect to the best performing ones in both metrics (p-value < 0.05, one-tail paired t-test).


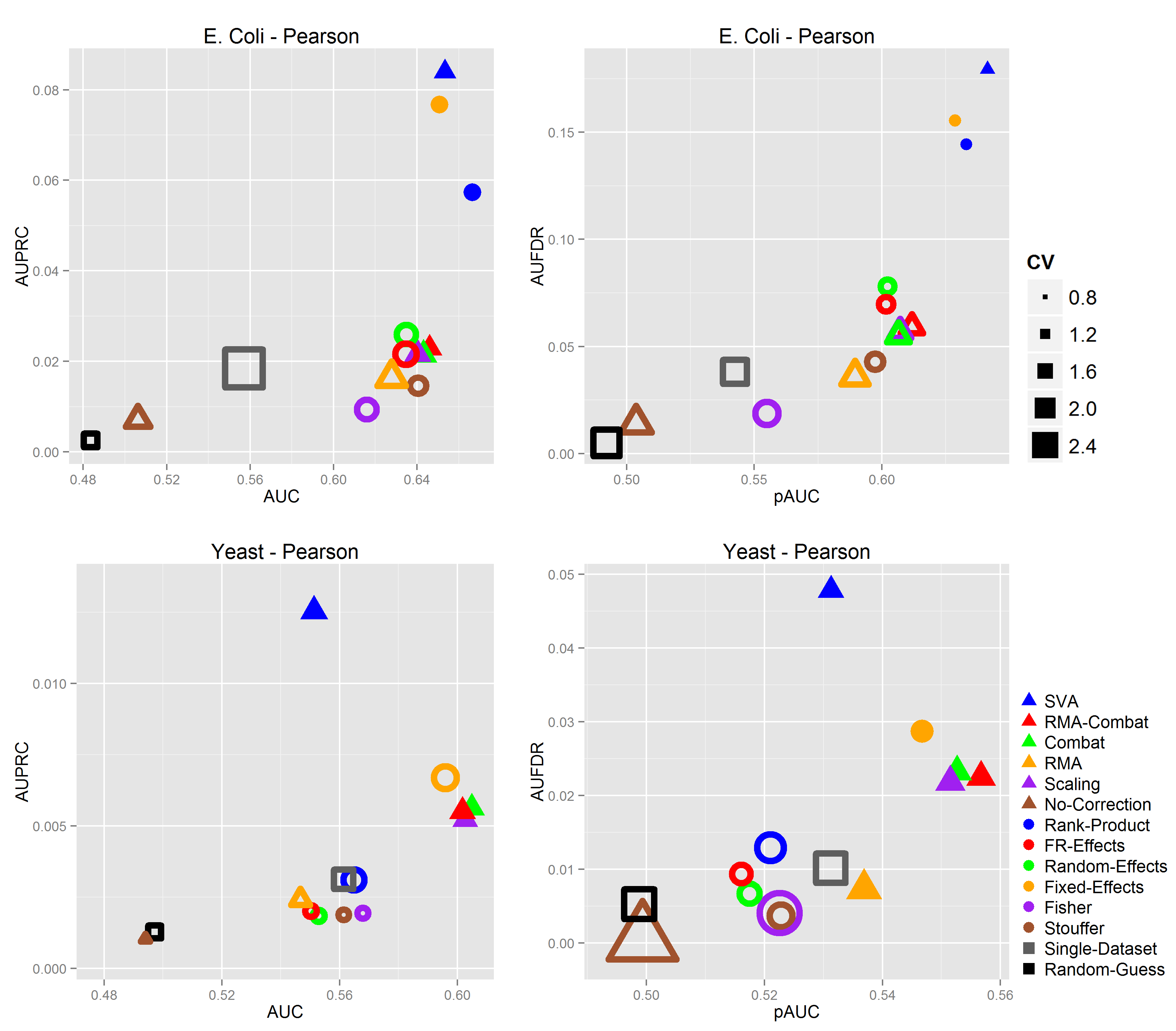


Figure S 2: results of the experimentations on E.Coli and Yeast compendia using the Pearson correlation p-values. Details as in Figure S 1. Methods generally achieve lower performances when p-values are used instead of correlations for ranking candidate gene-gene interactions. This is mainly due to the prevalence of close-to-zero p-values that create ties negatively affecting the performance metrics.


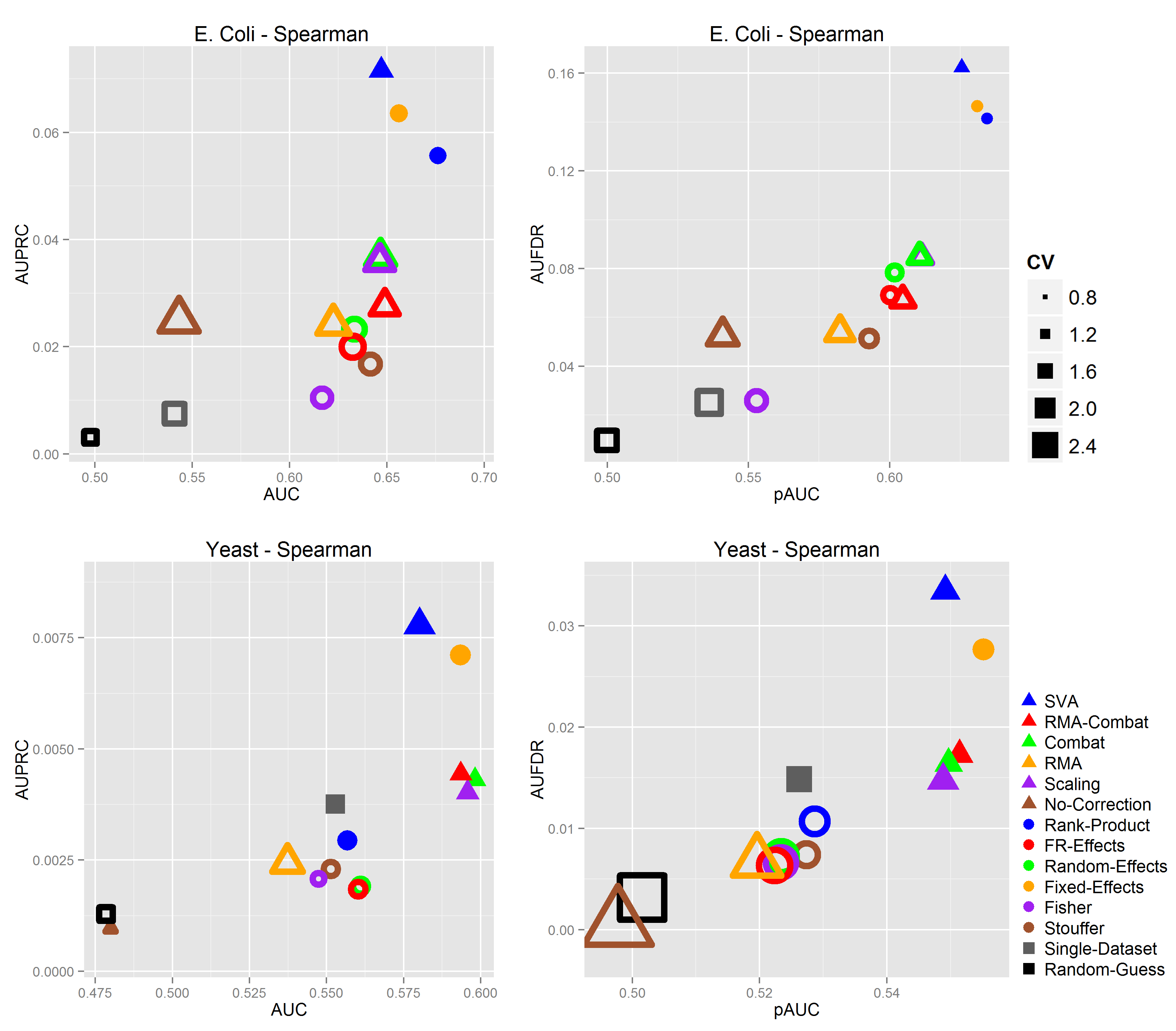


Figure S 3: results of the experimentations on E.Coli and Yeast compendia using the Spearman correlation p-values. Details as in Figure S 1. Methods generally achieve lower performances when p-values are used instead of correlations for ranking candidate gene-gene interactions. This is mainly due to the prevalence of close-to-zero p-values that create ties negatively affecting the performance metrics.


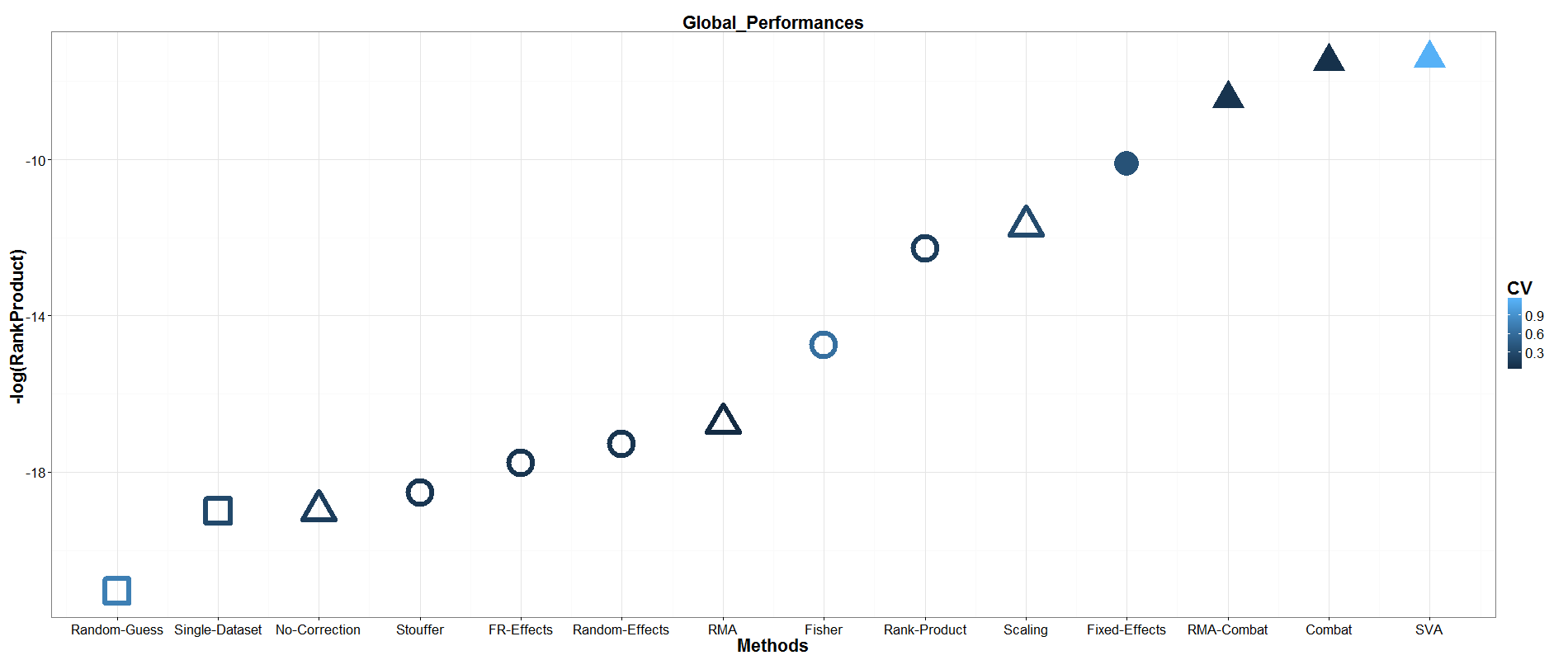


Figure S 4: rank-product analysis of MA and BER methods over AUC and AUPRC metrics (global metrics) Methods are ranked according to their performances, separately for each combination of data compendium (E. Coli and Yeast), correlation measure (Pearson and Spearman) and performance metric (AUC, AUPRC,), for a total of 8 different ranks. These ranks are then combined using the Rank-Product method, and the statistical significance of the ranks are evaluated with the method reported in [74]. The negative logarithm of the Rank-Product score is reported on the y-axis, while methods are listed on the x-axis. Triangular markers indicate BER methods, round markers MA methods, square markers baseline approaches. The color of each marker is directly proportional to the Coefficient of Variation (CV) of the respective log-transformed rank-product score (lighter color corresponds to higher variability). Methods that tend to be consistently ranked in the top positions are placed on the top-right of the plots, while poorly performing methods remain the in the bottom-left corner.


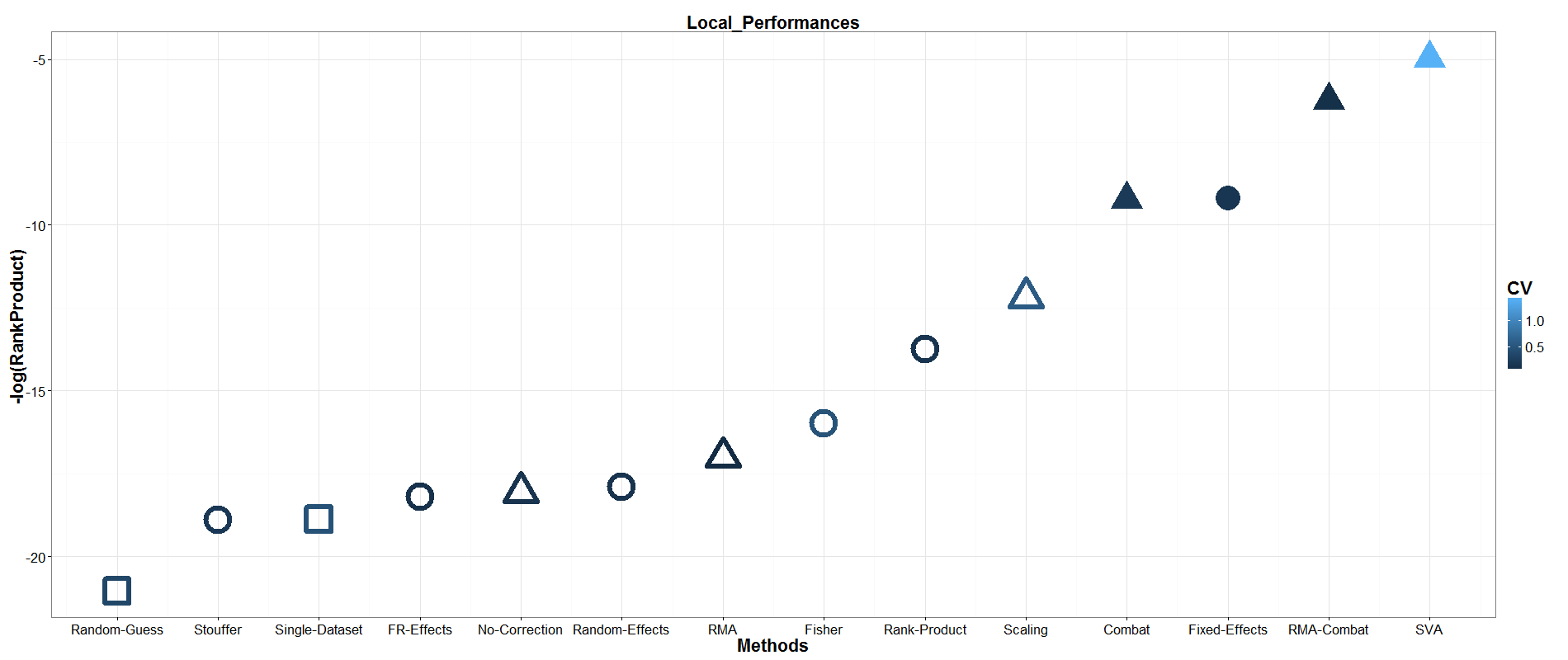


Figure S 5: rank-product analysis of MA and BER methods over pAUC and AUFDR metrics (local metrics). Details as reported in Figure S 3


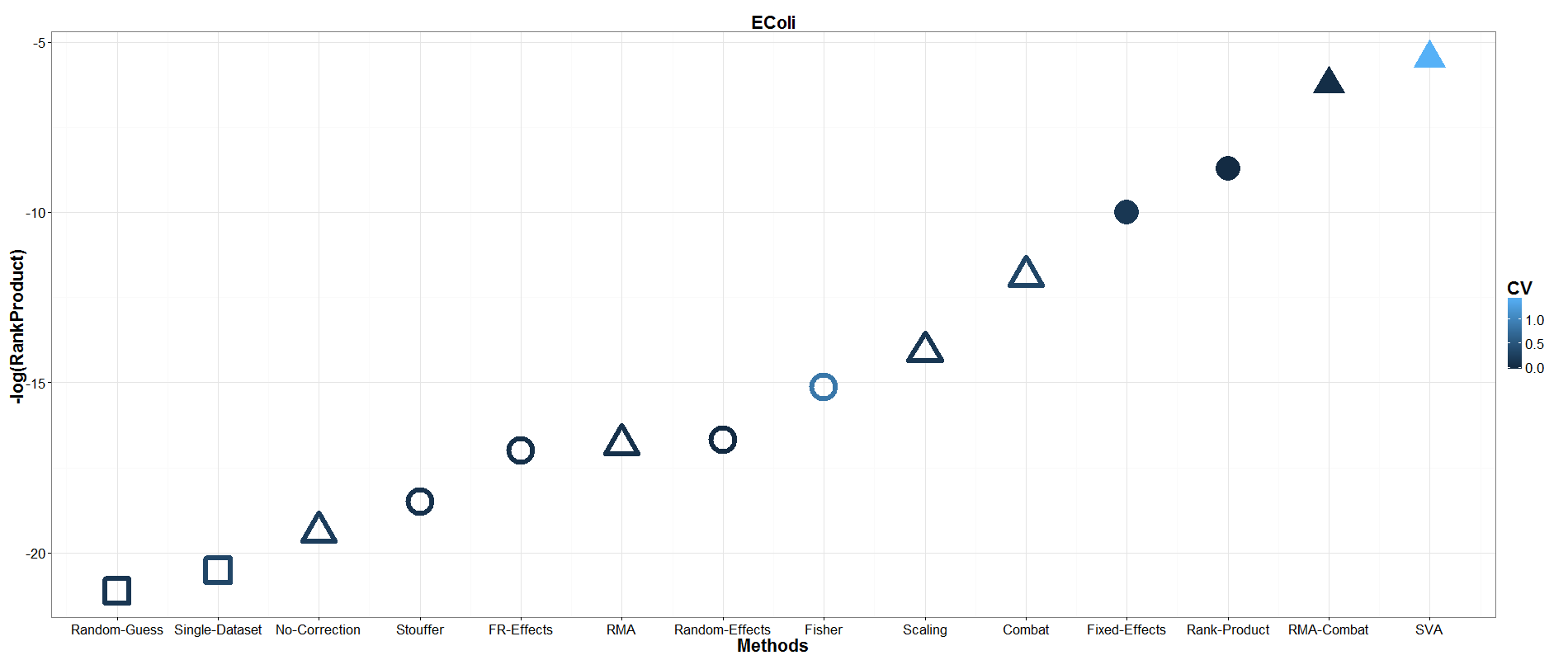


Figure S 6: rank-product analysis of MA and BER methods overall all metrics in the E. Coli compendium. Details as reported in Figure S 3


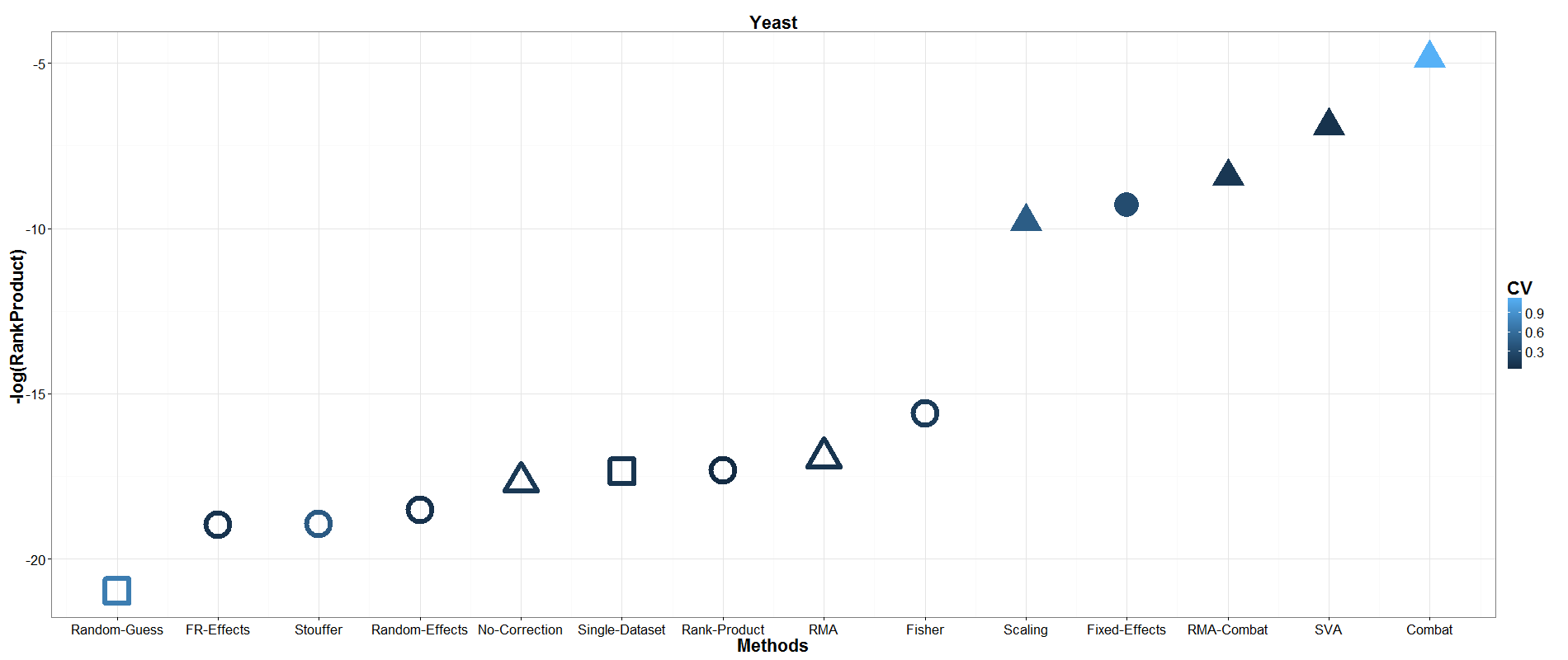


Figure S 7: rank-product analysis of MA and BER methods overall all metrics in the Yeast compendium. Details as reported in Figure S 3


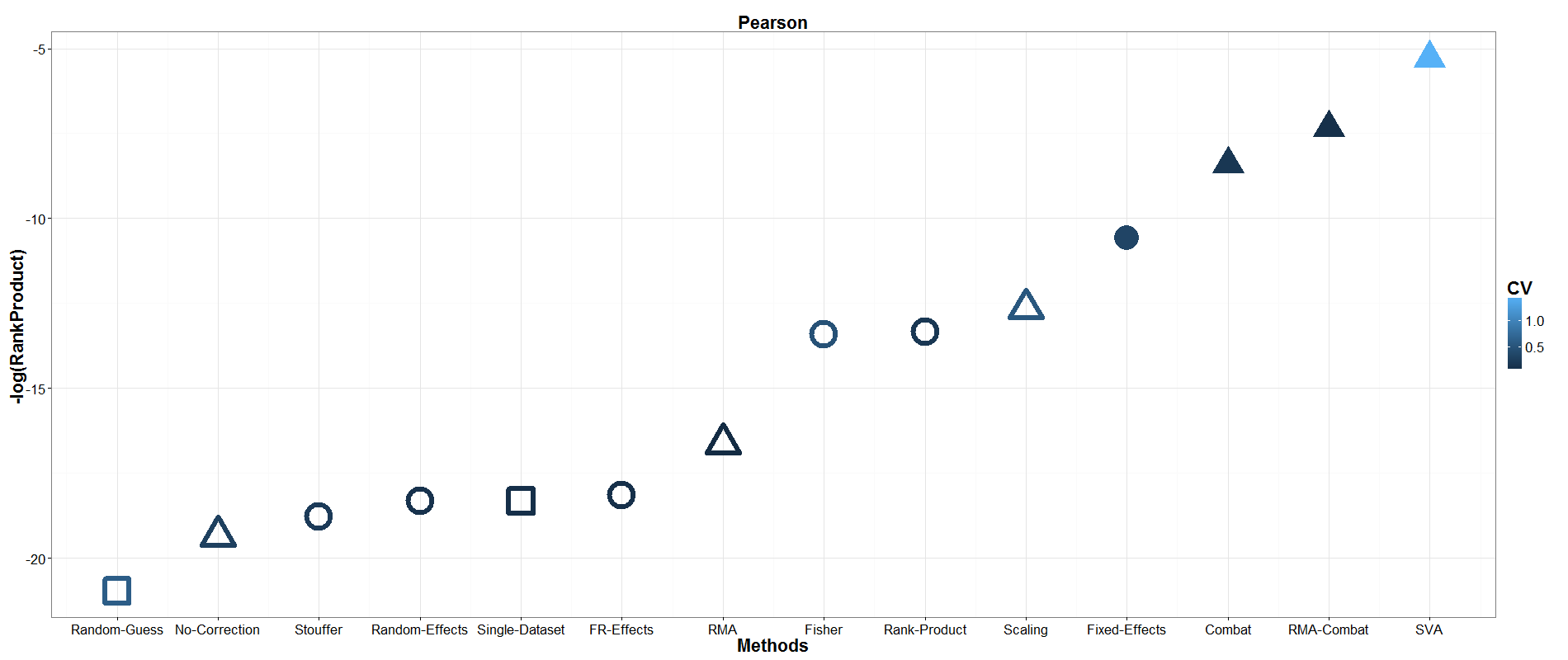


Figure S 8: rank-product analysis of MA and BER methods overall all metrics restricted only to the Pearson correlation measure. Details as reported in Figure S 3


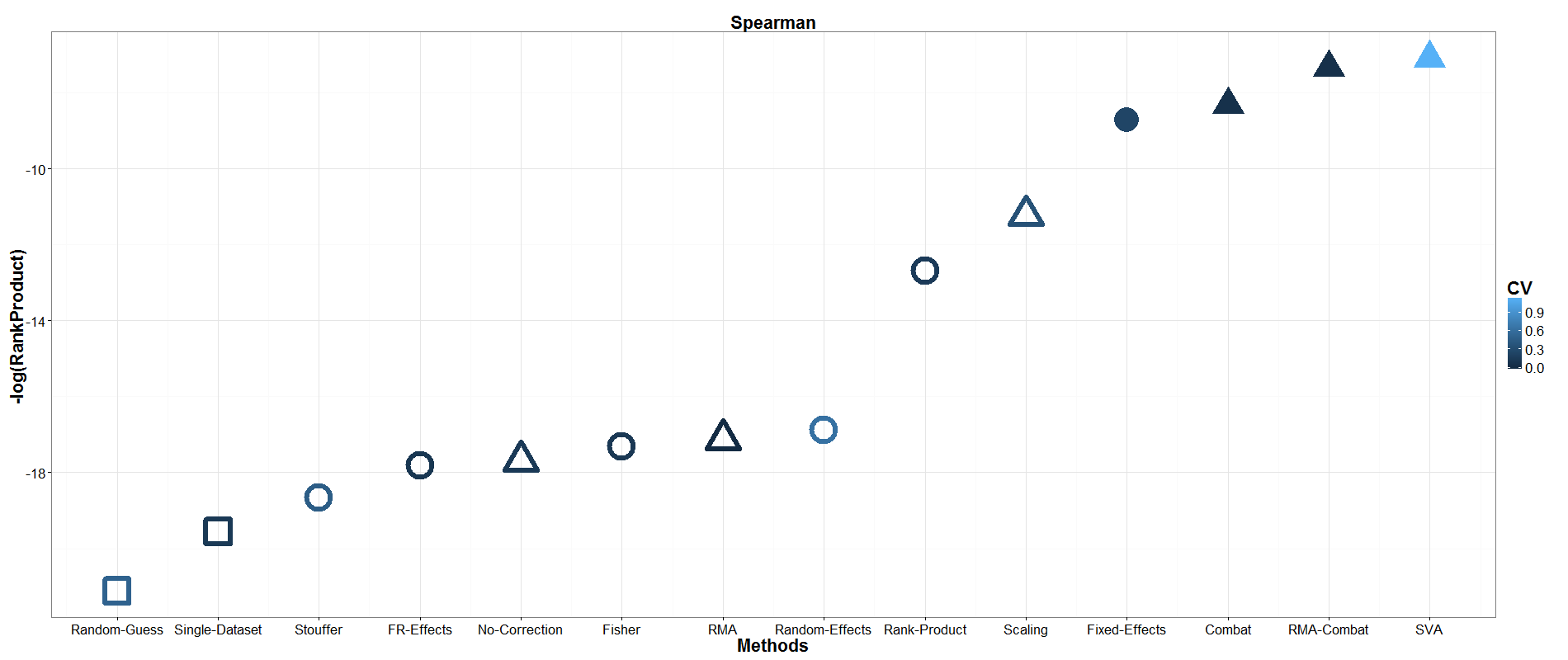


Figure S 9: rank-product analysis of MA and BER methods overall all metrics restricted only to the Spearman correlation measure. Details as reported in Figure S 3

Table S 4: details of the results on the E. Coli compendium. The table is subdivided in eight sub-tables delimited by black borders, one sub-table for each combination of the four metrics (AUC, pAUC, AUPRC and AUFDR) and correlation methods (Pearson and Spearman). Within each sub-table, methods are sorted according to their average performances. One-side, paired t-test p-values are reported as well, comparing each method with the first one and with the method immediately preceding. Performances are averaged over all transcript factors of the data compendium.

| Method | Average Performance | Performance St. Dev. | p-value vs. best method | p-value vs. previous method | Metric | Correlation method |
| --- | --- | --- | --- | --- | --- | --- |
| Rank-Product | 0.66633 | 0.19953 | 1 | 1 | AUC | Pearson |
| Fisher | 0.65519 | 0.20041 | 0.12755 | 0.12755 | AUC | Pearson |
| SVA | 0.6518 | 0.19173 | 0.21566 | 0.43364 | AUC | Pearson |
| RMA-Combat | 0.65105 | 0.21093 | 0.16634 | 0.48602 | AUC | Pearson |
| Fixed-Effects | 0.64922 | 0.21194 | 0.13611 | 0.21335 | AUC | Pearson |
| FR-Effects | 0.64865 | 0.19788 | 0.0885 | 0.48084 | AUC | Pearson |
| Combat | 0.64855 | 0.21543 | 0.1368 | 0.49684 | AUC | Pearson |
| Random-Effects | 0.64845 | 0.19779 | 0.08567 | 0.49681 | AUC | Pearson |
| Stouffer | 0.6478 | 0.20289 | 0.00169 | 0.48023 | AUC | Pearson |
| Scaling | 0.64746 | 0.21434 | 0.12057 | 0.49068 | AUC | Pearson |
| RMA | 0.63599 | 0.20616 | 0.04442 | 0.1789 | AUC | Pearson |
| No-Correction | 0.56086 | 0.2303 | 0 | 0.00003 | AUC | Pearson |
| Single-Dataset | 0.55706 | 0.17079 | 0 | 0.43783 | AUC | Pearson |
| Random-Guess | 0.48355 | 0.0955 | 0 | 0.00007 | AUC | Pearson |
| Rank-Product | 0.6742 | 0.19092 | 1 | 1 | AUC | Spearman |
| RMA-Combat | 0.65831 | 0.19725 | 0.125 | 0.125 | AUC | Spearman |
| Fixed-Effects | 0.65618 | 0.19864 | 0.09165 | 0.29956 | AUC | Spearman |
| Combat | 0.653 | 0.19956 | 0.06841 | 0.168 | AUC | Spearman |
| Scaling | 0.65218 | 0.19939 | 0.05867 | 0.20034 | AUC | Spearman |
| Fisher | 0.6485 | 0.18609 | 0.00565 | 0.37467 | AUC | Spearman |
| Random-Effects | 0.6485 | 0.20333 | 0.03199 | 0.49998 | AUC | Spearman |
| FR-Effects | 0.64845 | 0.20351 | 0.03191 | 0.34023 | AUC | Spearman |
| Stouffer | 0.64772 | 0.19211 | 0.00001 | 0.47843 | AUC | Spearman |
| SVA | 0.64631 | 0.20838 | 0.09036 | 0.47252 | AUC | Spearman |
| RMA | 0.63783 | 0.21341 | 0.02053 | 0.34127 | AUC | Spearman |
| No-Correction | 0.58894 | 0.24177 | 0.0001 | 0.00584 | AUC | Spearman |
| Single-Dataset | 0.54097 | 0.16164 | 0 | 0.0249 | AUC | Spearman |
| Random-Guess | 0.49781 | 0.11517 | 0 | 0.01215 | AUC | Spearman |
| SVA | 0.17939 | 0.23384 | 1 | 1 | AUFDR | Pearson |
| RMA-Combat | 0.15616 | 0.2272 | 0.07538 | 0.07538 | AUFDR | Pearson |
| Fixed-Effects | 0.15546 | 0.22691 | 0.06645 | 0.41983 | AUFDR | Pearson |
| Combat | 0.15372 | 0.22783 | 0.05023 | 0.22885 | AUFDR | Pearson |
| Scaling | 0.15168 | 0.22396 | 0.03866 | 0.09858 | AUFDR | Pearson |
| Rank-Product | 0.14443 | 0.20325 | 0.01032 | 0.17734 | AUFDR | Pearson |
| Fisher | 0.14137 | 0.21628 | 0.00915 | 0.36774 | AUFDR | Pearson |
| RMA | 0.13895 | 0.23046 | 0.00677 | 0.3868 | AUFDR | Pearson |
| Random-Effects | 0.13381 | 0.19554 | 0.00092 | 0.31036 | AUFDR | Pearson |
| FR-Effects | 0.13357 | 0.19555 | 0.00086 | 0.07599 | AUFDR | Pearson |
| Stouffer | 0.11968 | 0.18101 | 0.00009 | 0.00556 | AUFDR | Pearson |
| No-Correction | 0.08711 | 0.17757 | 0 | 0.00198 | AUFDR | Pearson |
| Single-Dataset | 0.03812 | 0.11187 | 0 | 0.00016 | AUFDR | Pearson |
| Random-Guess | 0.00498 | 0.01687 | 0 | 0.00137 | AUFDR | Pearson |
| SVA | 0.16228 | 0.22773 | 1 | 1 | AUFDR | Spearman |
| RMA-Combat | 0.14759 | 0.21766 | 0.14054 | 0.14054 | AUFDR | Spearman |
| Fixed-Effects | 0.14638 | 0.21436 | 0.13361 | 0.39069 | AUFDR | Spearman |
| Combat | 0.14464 | 0.21486 | 0.09264 | 0.32835 | AUFDR | Spearman |
| Scaling | 0.14175 | 0.21185 | 0.06261 | 0.00318 | AUFDR | Spearman |
| Rank-Product | 0.1414 | 0.20117 | 0.07196 | 0.48035 | AUFDR | Spearman |
| RMA | 0.12731 | 0.20228 | 0.00906 | 0.08382 | AUFDR | Spearman |
| Random-Effects | 0.12295 | 0.18499 | 0.00213 | 0.3145 | AUFDR | Spearman |
| FR-Effects | 0.12281 | 0.18477 | 0.00203 | 0.13004 | AUFDR | Spearman |
| Stouffer | 0.11841 | 0.17908 | 0.0009 | 0.20312 | AUFDR | Spearman |
| No-Correction | 0.11129 | 0.20158 | 0.0001 | 0.27693 | AUFDR | Spearman |
| Fisher | 0.10958 | 0.16952 | 0.00007 | 0.445 | AUFDR | Spearman |
| Single-Dataset | 0.0252 | 0.07253 | 0 | 0 | AUFDR | Spearman |
| Random-Guess | 0.00967 | 0.02363 | 0 | 0.01766 | AUFDR | Spearman |
| SVA | 0.08398 | 0.16291 | 1 | 1 | AUPRC | Pearson |
| RMA-Combat | 0.07719 | 0.16008 | 0.30753 | 0.30753 | AUPRC | Pearson |
| Fixed-Effects | 0.07675 | 0.16036 | 0.28617 | 0.43853 | AUPRC | Pearson |
| Combat | 0.07587 | 0.15932 | 0.25286 | 0.39525 | AUPRC | Pearson |
| RMA | 0.07458 | 0.16474 | 0.20814 | 0.43142 | AUPRC | Pearson |
| Scaling | 0.07341 | 0.15594 | 0.2011 | 0.44419 | AUPRC | Pearson |
| Fisher | 0.06576 | 0.14786 | 0.03637 | 0.20284 | AUPRC | Pearson |
| Rank-Product | 0.05738 | 0.12033 | 0.01272 | 0.17024 | AUPRC | Pearson |
| Random-Effects | 0.05247 | 0.11285 | 0.00254 | 0.03444 | AUPRC | Pearson |
| FR-Effects | 0.05242 | 0.11287 | 0.0025 | 0.14791 | AUPRC | Pearson |
| Stouffer | 0.04396 | 0.09992 | 0.0006 | 0.00729 | AUPRC | Pearson |
| No-Correction | 0.0429 | 0.12339 | 0.00133 | 0.43132 | AUPRC | Pearson |
| Single-Dataset | 0.01843 | 0.08565 | 0 | 0.00284 | AUPRC | Pearson |
| Random-Guess | 0.00252 | 0.00401 | 0 | 0.02666 | AUPRC | Pearson |
| SVA | 0.07157 | 0.15577 | 1 | 1 | AUPRC | Spearman |
| RMA-Combat | 0.0667 | 0.1348 | 0.34103 | 0.34103 | AUPRC | Spearman |
| Fixed-Effects | 0.06356 | 0.13561 | 0.24896 | 0.22119 | AUPRC | Spearman |
| Combat | 0.06307 | 0.13272 | 0.22114 | 0.43662 | AUPRC | Spearman |
| Scaling | 0.06097 | 0.12786 | 0.17288 | 0.00642 | AUPRC | Spearman |
| RMA | 0.05625 | 0.12289 | 0.10501 | 0.22445 | AUPRC | Spearman |
| Rank-Product | 0.05565 | 0.11512 | 0.06981 | 0.46568 | AUPRC | Spearman |
| No-Correction | 0.05287 | 0.1363 | 0.06222 | 0.36747 | AUPRC | Spearman |
| Random-Effects | 0.04641 | 0.09965 | 0.00908 | 0.20244 | AUPRC | Spearman |
| FR-Effects | 0.0463 | 0.09956 | 0.00879 | 0.09631 | AUPRC | Spearman |
| Stouffer | 0.04112 | 0.09157 | 0.00418 | 0.06518 | AUPRC | Spearman |
| Fisher | 0.04001 | 0.09179 | 0.00297 | 0.40483 | AUPRC | Spearman |
| Single-Dataset | 0.0075 | 0.01727 | 0.00001 | 0.00012 | AUPRC | Spearman |
| Random-Guess | 0.00307 | 0.00437 | 0 | 0.00347 | AUPRC | Spearman |
| SVA | 0.64061 | 0.15798 | 1 | 1 | pAUC | Pearson |
| RMA-Combat | 0.63109 | 0.1596 | 0.24704 | 0.24704 | pAUC | Pearson |
| Rank-Product | 0.63094 | 0.15537 | 0.22958 | 0.49273 | pAUC | Pearson |
| Fisher | 0.62835 | 0.14891 | 0.17403 | 0.36045 | pAUC | Pearson |
| Combat | 0.62818 | 0.1578 | 0.18132 | 0.48632 | pAUC | Pearson |
| Fixed-Effects | 0.62807 | 0.15736 | 0.17989 | 0.44854 | pAUC | Pearson |
| Scaling | 0.62745 | 0.15765 | 0.16896 | 0.26991 | pAUC | Pearson |
| FR-Effects | 0.62561 | 0.15639 | 0.12545 | 0.3672 | pAUC | Pearson |
| Random-Effects | 0.62561 | 0.15641 | 0.12536 | 0.47676 | pAUC | Pearson |
| Stouffer | 0.61732 | 0.1526 | 0.04525 | 0.10892 | pAUC | Pearson |
| RMA | 0.61382 | 0.15243 | 0.02537 | 0.33586 | pAUC | Pearson |
| No-Correction | 0.5687 | 0.14167 | 0 | 0.00001 | pAUC | Pearson |
| Single-Dataset | 0.54251 | 0.10661 | 0 | 0.02451 | pAUC | Pearson |
| Random-Guess | 0.49215 | 0.04045 | 0 | 0.00001 | pAUC | Pearson |
| Rank-Product | 0.63377 | 0.15181 | 1 | 1 | pAUC | Spearman |
| RMA-Combat | 0.63088 | 0.15766 | 0.36649 | 0.36649 | pAUC | Spearman |
| Fixed-Effects | 0.62953 | 0.15678 | 0.30441 | 0.30885 | pAUC | Spearman |
| Combat | 0.62804 | 0.15706 | 0.25092 | 0.22143 | pAUC | Spearman |
| Scaling | 0.62744 | 0.15683 | 0.22813 | 0.07955 | pAUC | Spearman |
| Random-Effects | 0.62655 | 0.15516 | 0.15728 | 0.43137 | pAUC | Spearman |
| FR-Effects | 0.6265 | 0.15513 | 0.15544 | 0.06389 | pAUC | Spearman |
| SVA | 0.62473 | 0.16439 | 0.2278 | 0.43969 | pAUC | Spearman |
| RMA | 0.61448 | 0.15431 | 0.02313 | 0.20204 | pAUC | Spearman |
| Stouffer | 0.60932 | 0.14963 | 0.00002 | 0.26884 | pAUC | Spearman |
| Fisher | 0.60844 | 0.1472 | 0.0005 | 0.44576 | pAUC | Spearman |
| No-Correction | 0.59206 | 0.1522 | 0.00044 | 0.07676 | pAUC | Spearman |
| Single-Dataset | 0.5361 | 0.09204 | 0 | 0.00013 | pAUC | Spearman |
| Random-Guess | 0.49982 | 0.05856 | 0 | 0.00053 | pAUC | Spearman |

Table S 5 details of the results on the Yeast compendium. Details as reported in Table S 4

| Method | Average Performance | Performance St. Dev. | p-value vs. best method | p-value vs. previous method | Metric | Correlation method |
| --- | --- | --- | --- | --- | --- | --- |
| Combat | 0.60413 | 0.1579 | 1 | 1 | AUC | Pearson |
| Scaling | 0.60056 | 0.15762 | 0.18012 | 0.18012 | AUC | Pearson |
| RMA-Combat | 0.60037 | 0.16394 | 0.22236 | 0.48744 | AUC | Pearson |
| Fixed-Effects | 0.59584 | 0.15889 | 0.03456 | 0.24406 | AUC | Pearson |
| Fisher | 0.57104 | 0.15141 | 0.04996 | 0.11103 | AUC | Pearson |
| Rank-Product | 0.56491 | 0.14564 | 0.03599 | 0.30938 | AUC | Pearson |
| Single-Dataset | 0.56149 | 0.16477 | 0.02825 | 0.45044 | AUC | Pearson |
| Stouffer | 0.56074 | 0.14677 | 0.02859 | 0.48867 | AUC | Pearson |
| Random-Effects | 0.55092 | 0.16774 | 0.00767 | 0.34439 | AUC | Pearson |
| FR-Effects | 0.55046 | 0.16711 | 0.00708 | 0.16234 | AUC | Pearson |
| SVA | 0.54991 | 0.15389 | 0.0077 | 0.49177 | AUC | Pearson |
| RMA | 0.54025 | 0.1973 | 0.02049 | 0.3802 | AUC | Pearson |
| Random-Guess | 0.49716 | 0.12891 | 0.0005 | 0.13352 | AUC | Pearson |
| No-Correction | 0.47817 | 0.19389 | 0.00002 | 0.30107 | AUC | Pearson |
| Combat | 0.59492 | 0.1586 | 1 | 1 | AUC | Spearman |
| Scaling | 0.5933 | 0.15555 | 0.26326 | 0.26326 | AUC | Spearman |
| Fixed-Effects | 0.59289 | 0.15556 | 0.38703 | 0.47437 | AUC | Spearman |
| RMA-Combat | 0.5918 | 0.16207 | 0.27122 | 0.44566 | AUC | Spearman |
| SVA | 0.57934 | 0.18016 | 0.24971 | 0.29862 | AUC | Spearman |
| Random-Effects | 0.55805 | 0.15803 | 0.02632 | 0.21584 | AUC | Spearman |
| FR-Effects | 0.55762 | 0.15654 | 0.02488 | 0.34285 | AUC | Spearman |
| Rank-Product | 0.55559 | 0.15487 | 0.03713 | 0.46084 | AUC | Spearman |
| Fisher | 0.55429 | 0.1707 | 0.03733 | 0.46346 | AUC | Spearman |
| Stouffer | 0.55061 | 0.16131 | 0.02028 | 0.351 | AUC | Spearman |
| Single-Dataset | 0.54994 | 0.17077 | 0.0352 | 0.48915 | AUC | Spearman |
| RMA | 0.53646 | 0.20119 | 0.01349 | 0.36114 | AUC | Spearman |
| No-Correction | 0.53425 | 0.18923 | 0.02057 | 0.47407 | AUC | Spearman |
| Random-Guess | 0.47846 | 0.15289 | 0.00161 | 0.08389 | AUC | Spearman |
| SVA | 0.01253 | 0.03127 | 1 | 1 | AUPRC | Pearson |
| Combat | 0.00723 | 0.02105 | 0.06384 | 0.06384 | AUPRC | Pearson |
| Fixed-Effects | 0.0067 | 0.01868 | 0.0464 | 0.12922 | AUPRC | Pearson |
| RMA-Combat | 0.00657 | 0.01879 | 0.04974 | 0.4222 | AUPRC | Pearson |
| Scaling | 0.00625 | 0.01511 | 0.04094 | 0.39191 | AUPRC | Pearson |
| RMA | 0.00611 | 0.02024 | 0.02935 | 0.47446 | AUPRC | Pearson |
| Fisher | 0.00586 | 0.01613 | 0.02652 | 0.42417 | AUPRC | Pearson |
| Rank-Product | 0.00311 | 0.00799 | 0.01277 | 0.03143 | AUPRC | Pearson |
| Single-Dataset | 0.00306 | 0.00739 | 0.01277 | 0.47895 | AUPRC | Pearson |
| No-Correction | 0.00288 | 0.00753 | 0.01167 | 0.29908 | AUPRC | Pearson |
| Random-Effects | 0.00204 | 0.0037 | 0.01279 | 0.25247 | AUPRC | Pearson |
| FR-Effects | 0.00204 | 0.0037 | 0.01277 | 0.34247 | AUPRC | Pearson |
| Stouffer | 0.00183 | 0.00226 | 0.01209 | 0.33853 | AUPRC | Pearson |
| Random-Guess | 0.00128 | 0.00183 | 0.01136 | 0.1041 | AUPRC | Pearson |
| SVA | 0.00778 | 0.02247 | 1 | 1 | AUPRC | Spearman |
| Combat | 0.00773 | 0.02119 | 0.48076 | 0.48076 | AUPRC | Spearman |
| RMA-Combat | 0.00731 | 0.02023 | 0.33164 | 0.04508 | AUPRC | Spearman |
| Scaling | 0.0072 | 0.01932 | 0.33978 | 0.40277 | AUPRC | Spearman |
| Fixed-Effects | 0.00711 | 0.0183 | 0.3669 | 0.44516 | AUPRC | Spearman |
| RMA | 0.00535 | 0.01695 | 0.06974 | 0.11344 | AUPRC | Spearman |
| No-Correction | 0.00478 | 0.01366 | 0.03267 | 0.34931 | AUPRC | Spearman |
| Fisher | 0.00465 | 0.01105 | 0.11349 | 0.4729 | AUPRC | Spearman |
| Single-Dataset | 0.00304 | 0.00752 | 0.03648 | 0.11899 | AUPRC | Spearman |
| Rank-Product | 0.00294 | 0.0072 | 0.05536 | 0.46652 | AUPRC | Spearman |
| Stouffer | 0.00257 | 0.0053 | 0.0529 | 0.25672 | AUPRC | Spearman |
| Random-Effects | 0.00254 | 0.00517 | 0.04655 | 0.47825 | AUPRC | Spearman |
| FR-Effects | 0.00254 | 0.00517 | 0.04647 | 0.19197 | AUPRC | Spearman |
| Random-Guess | 0.00129 | 0.0019 | 0.033 | 0.07209 | AUPRC | Spearman |
| RMA-Combat | 0.55477 | 0.11181 | 1 | 1 | pAUC | Pearson |
| Combat | 0.5527 | 0.1169 | 0.29276 | 0.29276 | pAUC | Pearson |
| Scaling | 0.54847 | 0.11387 | 0.08499 | 0.04117 | pAUC | Pearson |
| Fixed-Effects | 0.54579 | 0.11242 | 0.05188 | 0.22889 | pAUC | Pearson |
| Single-Dataset | 0.53164 | 0.10486 | 0.03118 | 0.11871 | pAUC | Pearson |
| SVA | 0.5299 | 0.12035 | 0.01356 | 0.4539 | pAUC | Pearson |
| Fisher | 0.52969 | 0.11101 | 0.03237 | 0.49426 | pAUC | Pearson |
| RMA | 0.52804 | 0.10552 | 0.05035 | 0.45928 | pAUC | Pearson |
| Stouffer | 0.52181 | 0.09621 | 0.01231 | 0.35131 | pAUC | Pearson |
| Rank-Product | 0.52102 | 0.08989 | 0.01002 | 0.43755 | pAUC | Pearson |
| No-Correction | 0.51309 | 0.10614 | 0.00084 | 0.31627 | pAUC | Pearson |
| FR-Effects | 0.50953 | 0.07963 | 0.00247 | 0.42054 | pAUC | Pearson |
| Random-Effects | 0.50914 | 0.07969 | 0.00232 | 0.06966 | pAUC | Pearson |
| Random-Guess | 0.4988 | 0.05739 | 0.00399 | 0.26914 | pAUC | Pearson |
| Fixed-Effects | 0.55508 | 0.11176 | 1 | 1 | pAUC | Spearman |
| RMA-Combat | 0.55014 | 0.11195 | 0.17036 | 0.17036 | pAUC | Spearman |
| SVA | 0.54713 | 0.12691 | 0.2432 | 0.37742 | pAUC | Spearman |
| Combat | 0.54633 | 0.11577 | 0.03765 | 0.46575 | pAUC | Spearman |
| Scaling | 0.54598 | 0.1132 | 0.01501 | 0.42969 | pAUC | Spearman |
| Fisher | 0.54179 | 0.11845 | 0.10458 | 0.36775 | pAUC | Spearman |
| No-Correction | 0.53982 | 0.12513 | 0.12225 | 0.45478 | pAUC | Spearman |
| Rank-Product | 0.52862 | 0.09925 | 0.00557 | 0.24123 | pAUC | Spearman |
| Stouffer | 0.52655 | 0.09891 | 0.00363 | 0.37425 | pAUC | Spearman |
| Single-Dataset | 0.52257 | 0.09308 | 0.0059 | 0.38937 | pAUC | Spearman |
| Random-Effects | 0.51833 | 0.09673 | 0.00466 | 0.39985 | pAUC | Spearman |
| FR-Effects | 0.51826 | 0.09684 | 0.00461 | 0.36913 | pAUC | Spearman |
| RMA | 0.51628 | 0.11519 | 0.00037 | 0.44382 | pAUC | Spearman |
| Random-Guess | 0.50155 | 0.06804 | 0.00804 | 0.23157 | pAUC | Spearman |
| SVA | 0.04784 | 0.1136 | 1 | 1 | AUFDR | Pearson |
| Combat | 0.02993 | 0.08888 | 0.04371 | 0.04371 | AUFDR | Pearson |
| Fixed-Effects | 0.02866 | 0.08298 | 0.03467 | 0.24648 | AUFDR | Pearson |
| RMA-Combat | 0.02822 | 0.08633 | 0.03505 | 0.41898 | AUFDR | Pearson |
| Scaling | 0.02484 | 0.07116 | 0.02658 | 0.15301 | AUFDR | Pearson |
| RMA | 0.02343 | 0.08641 | 0.00881 | 0.42755 | AUFDR | Pearson |
| Fisher | 0.02298 | 0.0694 | 0.0156 | 0.47321 | AUFDR | Pearson |
| No-Correction | 0.01865 | 0.0533 | 0.0112 | 0.24521 | AUFDR | Pearson |
| Rank-Product | 0.0129 | 0.04366 | 0.00732 | 0.16867 | AUFDR | Pearson |
| Single-Dataset | 0.01284 | 0.0433 | 0.00858 | 0.49488 | AUFDR | Pearson |
| FR-Effects | 0.00717 | 0.02818 | 0.00735 | 0.23836 | AUFDR | Pearson |
| Random-Effects | 0.00716 | 0.02818 | 0.00734 | 0.16145 | AUFDR | Pearson |
| Random-Guess | 0.00527 | 0.02017 | 0.00987 | 0.3641 | AUFDR | Pearson |
| Stouffer | 0.00364 | 0.01242 | 0.00586 | 0.32988 | AUFDR | Pearson |
| SVA | 0.03347 | 0.09183 | 1 | 1 | AUFDR | Spearman |
| Combat | 0.0305 | 0.08822 | 0.3029 | 0.3029 | AUFDR | Spearman |
| Scaling | 0.02884 | 0.08383 | 0.23295 | 0.19651 | AUFDR | Spearman |
| RMA-Combat | 0.02833 | 0.08597 | 0.18794 | 0.39033 | AUFDR | Spearman |
| Fixed-Effects | 0.02767 | 0.077 | 0.21901 | 0.42102 | AUFDR | Spearman |
| No-Correction | 0.02038 | 0.06614 | 0.02898 | 0.21072 | AUFDR | Spearman |
| RMA | 0.01929 | 0.07357 | 0.00914 | 0.43665 | AUFDR | Spearman |
| Fisher | 0.01804 | 0.0549 | 0.0587 | 0.42687 | AUFDR | Spearman |
| Random-Effects | 0.01156 | 0.03802 | 0.04315 | 0.08679 | AUFDR | Spearman |
| FR-Effects | 0.01154 | 0.03801 | 0.04302 | 0.16145 | AUFDR | Spearman |
| Single-Dataset | 0.01136 | 0.0419 | 0.01502 | 0.48875 | AUFDR | Spearman |
| Rank-Product | 0.01069 | 0.03463 | 0.02663 | 0.451 | AUFDR | Spearman |
| Stouffer | 0.01056 | 0.04113 | 0.05366 | 0.48542 | AUFDR | Spearman |
| Random-Guess | 0.00317 | 0.018 | 0.01984 | 0.14437 | AUFDR | Spearman |

Table S 6: results on the E. Coli compendium, performance calculated on the basis of the p-values. Details as in Table S 4.

| Method | Average Performance | Performance St. Dev. | p-value vs. best method | p-value vs. previous method | Metric | Correlation method |
| --- | --- | --- | --- | --- | --- | --- |
| Rank-Product | 0.66633 | 0.19953 | 1 | 1 | AUC | Pearson |
| SVA | 0.6518 | 0.19173 | 0.21566 | 0.21566 | AUC | Pearson |
| Fixed-Effects | 0.64922 | 0.21194 | 0.13611 | 0.45285 | AUC | Pearson |
| RMA-Combat | 0.64451 | 0.20583 | 0.08584 | 0.06155 | AUC | Pearson |
| Combat | 0.64088 | 0.20915 | 0.06013 | 0.03607 | AUC | Pearson |
| Stouffer | 0.64056 | 0.19617 | 0.00005 | 0.49128 | AUC | Pearson |
| Scaling | 0.64022 | 0.20823 | 0.05392 | 0.49063 | AUC | Pearson |
| Random-Effects | 0.63485 | 0.19666 | 0.02092 | 0.32931 | AUC | Pearson |
| FR-Effects | 0.63459 | 0.19693 | 0.02121 | 0.31444 | AUC | Pearson |
| RMA | 0.62784 | 0.1977 | 0.01472 | 0.3271 | AUC | Pearson |
| Fisher | 0.6159 | 0.17527 | 0 | 0.21446 | AUC | Pearson |
| Single-Dataset | 0.55706 | 0.17079 | 0 | 0.00104 | AUC | Pearson |
| No-Correction | 0.50622 | 0.09643 | 0 | 0.00261 | AUC | Pearson |
| Random-Guess | 0.48355 | 0.0955 | 0 | 0.04412 | AUC | Pearson |
| Rank-Product | 0.6742 | 0.19092 | 1 | 1 | AUC | Spearman |
| Fixed-Effects | 0.65618 | 0.19864 | 0.09165 | 0.09165 | AUC | Spearman |
| RMA-Combat | 0.64881 | 0.1917 | 0.03641 | 0.05323 | AUC | Spearman |
| Combat | 0.64665 | 0.19567 | 0.02892 | 0.21981 | AUC | Spearman |
| SVA | 0.64631 | 0.20838 | 0.09036 | 0.49306 | AUC | Spearman |
| Scaling | 0.64623 | 0.19562 | 0.02526 | 0.49824 | AUC | Spearman |
| Stouffer | 0.64158 | 0.18685 | 0 | 0.3603 | AUC | Spearman |
| Random-Effects | 0.63332 | 0.20354 | 0.00519 | 0.29659 | AUC | Spearman |
| FR-Effects | 0.63232 | 0.20363 | 0.00456 | 0.0063 | AUC | Spearman |
| RMA | 0.62249 | 0.20537 | 0.00209 | 0.29189 | AUC | Spearman |
| Fisher | 0.61682 | 0.15978 | 0 | 0.35479 | AUC | Spearman |
| No-Correction | 0.54328 | 0.18849 | 0 | 0.00028 | AUC | Spearman |
| Single-Dataset | 0.54097 | 0.16164 | 0 | 0.45563 | AUC | Spearman |
| Random-Guess | 0.49781 | 0.11517 | 0 | 0.01216 | AUC | Spearman |
| SVA | 0.08398 | 0.16291 | 1 | 1 | AUPRC | Pearson |
| Fixed-Effects | 0.07675 | 0.16036 | 0.28617 | 0.28617 | AUPRC | Pearson |
| Rank-Product | 0.05738 | 0.12033 | 0.01272 | 0.00861 | AUPRC | Pearson |
| Random-Effects | 0.02591 | 0.05888 | 0.00002 | 0.00006 | AUPRC | Pearson |
| RMA-Combat | 0.02287 | 0.0493 | 0.00006 | 0.32622 | AUPRC | Pearson |
| FR-Effects | 0.02162 | 0.0553 | 0.00001 | 0.42544 | AUPRC | Pearson |
| Scaling | 0.02162 | 0.05119 | 0.00004 | 0.49972 | AUPRC | Pearson |
| Combat | 0.02147 | 0.05121 | 0.00004 | 0.34393 | AUPRC | Pearson |
| Single-Dataset | 0.01843 | 0.08565 | 0 | 0.37332 | AUPRC | Pearson |
| RMA | 0.01638 | 0.0405 | 0.00001 | 0.4093 | AUPRC | Pearson |
| Stouffer | 0.01466 | 0.02776 | 0 | 0.24682 | AUPRC | Pearson |
| Fisher | 0.00936 | 0.02183 | 0 | 0.00029 | AUPRC | Pearson |
| No-Correction | 0.0071 | 0.01565 | 0 | 0.13685 | AUPRC | Pearson |
| Random-Guess | 0.00252 | 0.00401 | 0 | 0.00094 | AUPRC | Pearson |
| SVA | 0.07157 | 0.15577 | 1 | 1 | AUPRC | Spearman |
| Fixed-Effects | 0.06356 | 0.13561 | 0.24896 | 0.24896 | AUPRC | Spearman |
| Rank-Product | 0.05565 | 0.11512 | 0.06981 | 0.03145 | AUPRC | Spearman |
| Combat | 0.03683 | 0.09496 | 0.00671 | 0.0214 | AUPRC | Spearman |
| Scaling | 0.03586 | 0.09028 | 0.00562 | 0.0622 | AUPRC | Spearman |
| RMA-Combat | 0.02758 | 0.06811 | 0.001 | 0.16268 | AUPRC | Spearman |
| No-Correction | 0.02494 | 0.08723 | 0.00077 | 0.36477 | AUPRC | Spearman |
| RMA | 0.02421 | 0.06886 | 0.00018 | 0.46391 | AUPRC | Spearman |
| Random-Effects | 0.02331 | 0.0572 | 0.00016 | 0.45273 | AUPRC | Spearman |
| FR-Effects | 0.02002 | 0.05275 | 0.00007 | 0.0017 | AUPRC | Spearman |
| Stouffer | 0.01676 | 0.0378 | 0.0001 | 0.29311 | AUPRC | Spearman |
| Fisher | 0.01045 | 0.0228 | 0.00003 | 0.0005 | AUPRC | Spearman |
| Single-Dataset | 0.0075 | 0.01727 | 0.00001 | 0.12822 | AUPRC | Spearman |
| Random-Guess | 0.00307 | 0.00437 | 0 | 0.00347 | AUPRC | Spearman |
| SVA | 0.64061 | 0.15798 | 1 | 1 | pAUC | Pearson |
| Rank-Product | 0.63094 | 0.15537 | 0.22958 | 0.22958 | pAUC | Pearson |
| Fixed-Effects | 0.62807 | 0.15736 | 0.17989 | 0.3547 | pAUC | Pearson |
| RMA-Combat | 0.61172 | 0.13927 | 0.02161 | 0.00108 | pAUC | Pearson |
| Scaling | 0.60716 | 0.13478 | 0.00812 | 0.00641 | pAUC | Pearson |
| Combat | 0.60646 | 0.13452 | 0.00705 | 0.1327 | pAUC | Pearson |
| Random-Effects | 0.60223 | 0.14385 | 0.00221 | 0.29147 | pAUC | Pearson |
| FR-Effects | 0.60168 | 0.14349 | 0.00197 | 0.19528 | pAUC | Pearson |
| Stouffer | 0.59738 | 0.12949 | 0.00124 | 0.31631 | pAUC | Pearson |
| RMA | 0.58943 | 0.12544 | 0.00019 | 0.17441 | pAUC | Pearson |
| Fisher | 0.55506 | 0.09323 | 0 | 0.00007 | pAUC | Pearson |
| Single-Dataset | 0.54251 | 0.10661 | 0 | 0.11735 | pAUC | Pearson |
| No-Correction | 0.50376 | 0.02685 | 0 | 0.00007 | pAUC | Pearson |
| Random-Guess | 0.49215 | 0.04045 | 0 | 0.00711 | pAUC | Pearson |
| Rank-Product | 0.63377 | 0.15181 | 1 | 1 | pAUC | Spearman |
| Fixed-Effects | 0.62953 | 0.15678 | 0.30441 | 0.30441 | pAUC | Spearman |
| SVA | 0.62473 | 0.16439 | 0.2278 | 0.3352 | pAUC | Spearman |
| Scaling | 0.61108 | 0.14403 | 0.00972 | 0.13138 | pAUC | Spearman |
| Combat | 0.61062 | 0.14408 | 0.00966 | 0.21394 | pAUC | Spearman |
| RMA-Combat | 0.60468 | 0.1377 | 0.0021 | 0.09711 | pAUC | Spearman |
| Random-Effects | 0.60184 | 0.14158 | 0.00047 | 0.37586 | pAUC | Spearman |
| FR-Effects | 0.60017 | 0.14076 | 0.00029 | 0.01105 | pAUC | Spearman |
| Stouffer | 0.59266 | 0.13165 | 0 | 0.1834 | pAUC | Spearman |
| RMA | 0.58238 | 0.13263 | 0.00001 | 0.13428 | pAUC | Spearman |
| Fisher | 0.55287 | 0.09613 | 0 | 0.00079 | pAUC | Spearman |
| No-Correction | 0.54086 | 0.11075 | 0 | 0.17961 | pAUC | Spearman |
| Single-Dataset | 0.53609 | 0.09203 | 0 | 0.35838 | pAUC | Spearman |
| Random-Guess | 0.49982 | 0.05856 | 0 | 0.00053 | pAUC | Spearman |
| SVA | 0.17939 | 0.23384 | 1 | 1 | AUFDR | Pearson |
| Fixed-Effects | 0.15546 | 0.22691 | 0.06645 | 0.06645 | AUFDR | Pearson |
| Rank-Product | 0.14443 | 0.20325 | 0.01032 | 0.08685 | AUFDR | Pearson |
| Random-Effects | 0.07807 | 0.12981 | 0 | 0 | AUFDR | Pearson |
| FR-Effects | 0.06962 | 0.12084 | 0 | 0 | AUFDR | Pearson |
| RMA-Combat | 0.05909 | 0.11666 | 0 | 0.21661 | AUFDR | Pearson |
| Scaling | 0.05697 | 0.11211 | 0 | 0.19734 | AUFDR | Pearson |
| Combat | 0.05502 | 0.11102 | 0 | 0.01907 | AUFDR | Pearson |
| Stouffer | 0.0429 | 0.08546 | 0 | 0.02949 | AUFDR | Pearson |
| Single-Dataset | 0.03812 | 0.11187 | 0 | 0.35487 | AUFDR | Pearson |
| RMA | 0.03593 | 0.08995 | 0 | 0.43434 | AUFDR | Pearson |
| Fisher | 0.01871 | 0.05527 | 0 | 0.00056 | AUFDR | Pearson |
| No-Correction | 0.01401 | 0.04174 | 0 | 0.17281 | AUFDR | Pearson |
| Random-Guess | 0.00498 | 0.01687 | 0 | 0.01838 | AUFDR | Pearson |
| SVA | 0.16228 | 0.22773 | 1 | 1 | AUFDR | Spearman |
| Fixed-Effects | 0.14638 | 0.21436 | 0.13361 | 0.13361 | AUFDR | Spearman |
| Rank-Product | 0.1414 | 0.20117 | 0.07196 | 0.24512 | AUFDR | Spearman |
| Combat | 0.08502 | 0.16325 | 0.00008 | 0.00047 | AUFDR | Spearman |
| Scaling | 0.08481 | 0.16133 | 0.00007 | 0.40896 | AUFDR | Spearman |
| Random-Effects | 0.0784 | 0.12784 | 0 | 0.32192 | AUFDR | Spearman |
| FR-Effects | 0.06912 | 0.11546 | 0 | 0 | AUFDR | Spearman |
| RMA-Combat | 0.06716 | 0.13644 | 0 | 0.44409 | AUFDR | Spearman |
| RMA | 0.05399 | 0.13368 | 0 | 0.04274 | AUFDR | Spearman |
| No-Correction | 0.05253 | 0.14215 | 0 | 0.45854 | AUFDR | Spearman |
| Stouffer | 0.05135 | 0.09698 | 0 | 0.46699 | AUFDR | Spearman |
| Fisher | 0.02586 | 0.06069 | 0 | 0.00002 | AUFDR | Spearman |
| Single-Dataset | 0.0252 | 0.07253 | 0 | 0.47006 | AUFDR | Spearman |
| Random-Guess | 0.00967 | 0.02363 | 0 | 0.01768 | AUFDR | Spearman |

Table S 7: results on the Yeast compendium, performance calculated on the basis of the p-values. Details as in Table S 4.

| Method | Average Performance | Performance St. Dev. | p-value vs. best method | p-value vs. previous method | Metric | Correlation method |
| --- | --- | --- | --- | --- | --- | --- |
| Combat | 0.60405 | 0.15761 | 1 | 1 | AUC | Pearson |
| Scaling | 0.60074 | 0.15766 | 0.19981 | 0.19981 | AUC | Pearson |
| RMA-Combat | 0.60019 | 0.16347 | 0.21633 | 0.46225 | AUC | Pearson |
| Fixed-Effects | 0.59584 | 0.15889 | 0.03703 | 0.25353 | AUC | Pearson |
| Fisher | 0.56794 | 0.13822 | 0.0371 | 0.08646 | AUC | Pearson |
| Rank-Product | 0.56491 | 0.14564 | 0.03635 | 0.41029 | AUC | Pearson |
| Single-Dataset | 0.56135 | 0.16449 | 0.02833 | 0.44844 | AUC | Pearson |
| Stouffer | 0.56134 | 0.14592 | 0.03002 | 0.49974 | AUC | Pearson |
| Random-Effects | 0.55286 | 0.16437 | 0.01085 | 0.3849 | AUC | Pearson |
| FR-Effects | 0.5503 | 0.16376 | 0.00823 | 0.00013 | AUC | Pearson |
| SVA | 0.54991 | 0.15389 | 0.00771 | 0.49434 | AUC | Pearson |
| RMA | 0.5467 | 0.18909 | 0.03012 | 0.45967 | AUC | Pearson |
| Random-Guess | 0.49716 | 0.12891 | 0.0005 | 0.09377 | AUC | Pearson |
| No-Correction | 0.49401 | 0.04329 | 0.00001 | 0.43949 | AUC | Pearson |
| Combat | 0.59584 | 0.15892 | 1 | 1 | AUC | Spearman |
| Scaling | 0.59368 | 0.15533 | 0.20548 | 0.20548 | AUC | Spearman |
| Fixed-Effects | 0.59289 | 0.15556 | 0.34355 | 0.45205 | AUC | Spearman |
| RMA-Combat | 0.59196 | 0.16164 | 0.22554 | 0.45367 | AUC | Spearman |
| SVA | 0.57934 | 0.18016 | 0.23665 | 0.29614 | AUC | Spearman |
| Random-Effects | 0.56094 | 0.16594 | 0.04815 | 0.25952 | AUC | Spearman |
| FR-Effects | 0.56026 | 0.16387 | 0.04507 | 0.32391 | AUC | Spearman |
| Rank-Product | 0.55559 | 0.15487 | 0.03592 | 0.43115 | AUC | Spearman |
| Stouffer | 0.55131 | 0.16094 | 0.02023 | 0.32149 | AUC | Spearman |
| Single-Dataset | 0.55083 | 0.17088 | 0.03572 | 0.49224 | AUC | Spearman |
| Fisher | 0.54744 | 0.15553 | 0.01833 | 0.44391 | AUC | Spearman |
| RMA | 0.53728 | 0.1877 | 0.01041 | 0.38074 | AUC | Spearman |
| No-Correction | 0.47994 | 0.06658 | 0.00002 | 0.02568 | AUC | Spearman |
| Random-Guess | 0.47846 | 0.15289 | 0.00146 | 0.47839 | AUC | Spearman |
| SVA | 0.01253 | 0.03127 | 1 | 1 | AUPRC | Pearson |
| Fixed-Effects | 0.0067 | 0.01868 | 0.0464 | 0.0464 | AUPRC | Pearson |
| Combat | 0.00563 | 0.01308 | 0.04059 | 0.20906 | AUPRC | Pearson |
| RMA-Combat | 0.00552 | 0.013 | 0.04067 | 0.24796 | AUPRC | Pearson |
| Scaling | 0.00524 | 0.01207 | 0.02683 | 0.36581 | AUPRC | Pearson |
| Single-Dataset | 0.00313 | 0.00709 | 0.01523 | 0.02749 | AUPRC | Pearson |
| Rank-Product | 0.00311 | 0.00799 | 0.01277 | 0.49136 | AUPRC | Pearson |
| RMA | 0.00242 | 0.0037 | 0.01395 | 0.22624 | AUPRC | Pearson |
| FR-Effects | 0.00202 | 0.00258 | 0.0156 | 0.28357 | AUPRC | Pearson |
| Fisher | 0.00194 | 0.00243 | 0.01101 | 0.44307 | AUPRC | Pearson |
| Stouffer | 0.00188 | 0.00229 | 0.0129 | 0.44042 | AUPRC | Pearson |
| Random-Effects | 0.00185 | 0.00253 | 0.01413 | 0.45526 | AUPRC | Pearson |
| Random-Guess | 0.00128 | 0.00183 | 0.01136 | 0.11822 | AUPRC | Pearson |
| No-Correction | 0.00103 | 0.00085 | 0.00895 | 0.21229 | AUPRC | Pearson |
| SVA | 0.00778 | 0.02247 | 1 | 1 | AUPRC | Spearman |
| Fixed-Effects | 0.00711 | 0.0183 | 0.3669 | 0.3669 | AUPRC | Spearman |
| RMA-Combat | 0.00444 | 0.00887 | 0.08708 | 0.04706 | AUPRC | Spearman |
| Combat | 0.00431 | 0.00852 | 0.07563 | 0.25057 | AUPRC | Spearman |
| Scaling | 0.00402 | 0.00842 | 0.05421 | 0.20728 | AUPRC | Spearman |
| Single-Dataset | 0.00376 | 0.00839 | 0.10257 | 0.4251 | AUPRC | Spearman |
| Rank-Product | 0.00294 | 0.0072 | 0.05536 | 0.30603 | AUPRC | Spearman |
| RMA | 0.00244 | 0.0065 | 0.03922 | 0.01659 | AUPRC | Spearman |
| Stouffer | 0.0023 | 0.00381 | 0.03973 | 0.41292 | AUPRC | Spearman |
| Fisher | 0.00208 | 0.00269 | 0.04727 | 0.35085 | AUPRC | Spearman |
| Random-Effects | 0.00191 | 0.0033 | 0.03826 | 0.39603 | AUPRC | Spearman |
| FR-Effects | 0.00184 | 0.00312 | 0.03687 | 0.07627 | AUPRC | Spearman |
| Random-Guess | 0.00129 | 0.0019 | 0.033 | 0.16654 | AUPRC | Spearman |
| No-Correction | 0.00096 | 0.00083 | 0.02508 | 0.15486 | AUPRC | Spearman |
| RMA-Combat | 0.55427 | 0.10973 | 1 | 1 | pAUC | Pearson |
| Combat | 0.55247 | 0.11564 | 0.31579 | 0.31579 | pAUC | Pearson |
| Scaling | 0.54899 | 0.11357 | 0.12359 | 0.07906 | pAUC | Pearson |
| Fixed-Effects | 0.54579 | 0.11242 | 0.06456 | 0.2022 | pAUC | Pearson |
| RMA | 0.53673 | 0.09709 | 0.14555 | 0.29735 | pAUC | Pearson |
| Single-Dataset | 0.53126 | 0.10368 | 0.03248 | 0.37883 | pAUC | Pearson |
| SVA | 0.5299 | 0.12035 | 0.0161 | 0.46394 | pAUC | Pearson |
| Stouffer | 0.52281 | 0.0923 | 0.01173 | 0.31987 | pAUC | Pearson |
| Fisher | 0.52253 | 0.07755 | 0.00767 | 0.48149 | pAUC | Pearson |
| Rank-Product | 0.52102 | 0.08989 | 0.01073 | 0.42317 | pAUC | Pearson |
| Random-Effects | 0.51749 | 0.07518 | 0.00288 | 0.41258 | pAUC | Pearson |
| FR-Effects | 0.51609 | 0.07514 | 0.00241 | 0.16081 | pAUC | Pearson |
| No-Correction | 0.49934 | 0.00483 | 0.00087 | 0.07332 | pAUC | Pearson |
| Random-Guess | 0.4988 | 0.05739 | 0.00381 | 0.47555 | pAUC | Pearson |
| Fixed-Effects | 0.55508 | 0.11176 | 1 | 1 | pAUC | Spearman |
| RMA-Combat | 0.55052 | 0.10929 | 0.21241 | 0.21241 | pAUC | Spearman |
| Combat | 0.54886 | 0.1141 | 0.17007 | 0.32973 | pAUC | Spearman |
| SVA | 0.54713 | 0.12691 | 0.2432 | 0.41924 | pAUC | Spearman |
| Scaling | 0.547 | 0.11061 | 0.0566 | 0.49414 | pAUC | Spearman |
| Rank-Product | 0.52862 | 0.09925 | 0.00557 | 0.05191 | pAUC | Spearman |
| Stouffer | 0.52735 | 0.09722 | 0.00509 | 0.4257 | pAUC | Spearman |
| Single-Dataset | 0.5254 | 0.09252 | 0.01335 | 0.44482 | pAUC | Spearman |
| Random-Effects | 0.5234 | 0.08644 | 0.01851 | 0.45317 | pAUC | Spearman |
| Fisher | 0.52338 | 0.07851 | 0.01018 | 0.49945 | pAUC | Spearman |
| FR-Effects | 0.52236 | 0.08546 | 0.01831 | 0.47611 | pAUC | Spearman |
| RMA | 0.51961 | 0.09528 | 0.00077 | 0.40441 | pAUC | Spearman |
| Random-Guess | 0.50155 | 0.06804 | 0.00804 | 0.15369 | pAUC | Spearman |
| No-Correction | 0.49771 | 0.00765 | 0.00065 | 0.35734 | pAUC | Spearman |
| SVA | 0.04784 | 0.1136 | 1 | 1 | AUFDR | Pearson |
| Fixed-Effects | 0.02866 | 0.08298 | 0.03467 | 0.03467 | AUFDR | Pearson |
| Combat | 0.02321 | 0.06313 | 0.02869 | 0.12366 | AUFDR | Pearson |
| RMA-Combat | 0.0225 | 0.06132 | 0.02998 | 0.21606 | AUFDR | Pearson |
| Scaling | 0.02182 | 0.06052 | 0.01837 | 0.40423 | AUFDR | Pearson |
| Rank-Product | 0.0129 | 0.04366 | 0.00732 | 0.05606 | AUFDR | Pearson |
| Single-Dataset | 0.01014 | 0.03709 | 0.00624 | 0.2691 | AUFDR | Pearson |
| FR-Effects | 0.00939 | 0.02184 | 0.01706 | 0.45621 | AUFDR | Pearson |
| RMA | 0.00734 | 0.02473 | 0.00766 | 0.35009 | AUFDR | Pearson |
| Random-Effects | 0.00668 | 0.01697 | 0.01105 | 0.44559 | AUFDR | Pearson |
| Random-Guess | 0.00527 | 0.02017 | 0.00987 | 0.36636 | AUFDR | Pearson |
| Fisher | 0.00407 | 0.02104 | 0.00478 | 0.39369 | AUFDR | Pearson |
| Stouffer | 0.00369 | 0.01055 | 0.00644 | 0.4565 | AUFDR | Pearson |
| No-Correction | 0.00042 | 0.00276 | 0.00387 | 0.02795 | AUFDR | Pearson |
| SVA | 0.03347 | 0.09183 | 1 | 1 | AUFDR | Spearman |
| Fixed-Effects | 0.02767 | 0.077 | 0.21901 | 0.21901 | AUFDR | Spearman |
| RMA-Combat | 0.01729 | 0.04413 | 0.05066 | 0.05646 | AUFDR | Spearman |
| Combat | 0.01641 | 0.04296 | 0.03745 | 0.21741 | AUFDR | Spearman |
| Single-Dataset | 0.01485 | 0.04816 | 0.08092 | 0.42771 | AUFDR | Spearman |
| Scaling | 0.01474 | 0.04433 | 0.02124 | 0.49471 | AUFDR | Spearman |
| Rank-Product | 0.01069 | 0.03463 | 0.02663 | 0.23081 | AUFDR | Spearman |
| Stouffer | 0.0074 | 0.02137 | 0.02812 | 0.21793 | AUFDR | Spearman |
| Random-Effects | 0.00719 | 0.02871 | 0.03071 | 0.48186 | AUFDR | Spearman |
| RMA | 0.0067 | 0.0306 | 0.01699 | 0.4317 | AUFDR | Spearman |
| Fisher | 0.00665 | 0.02596 | 0.03266 | 0.49668 | AUFDR | Spearman |
| FR-Effects | 0.00637 | 0.02563 | 0.02647 | 0.48021 | AUFDR | Spearman |
| Random-Guess | 0.00317 | 0.018 | 0.01984 | 0.25466 | AUFDR | Spearman |
| No-Correction | 0.00046 | 0.00306 | 0.01051 | 0.16621 | AUFDR | Spearman |

# Supplementary results on simulated data

Additional Files 2 and 3 report the numerical results on all simulated scenarios.


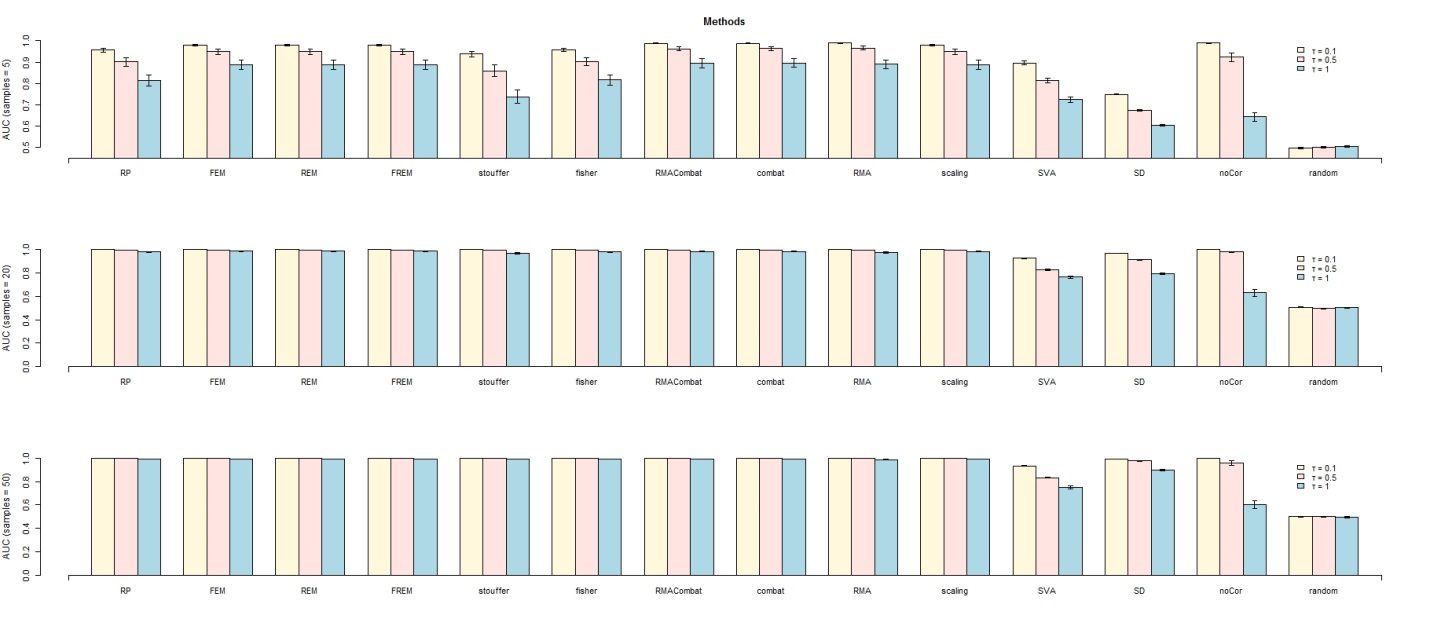


Figure S 10: AUC results on simulated data for different sample sizes using Pearson Correlations.


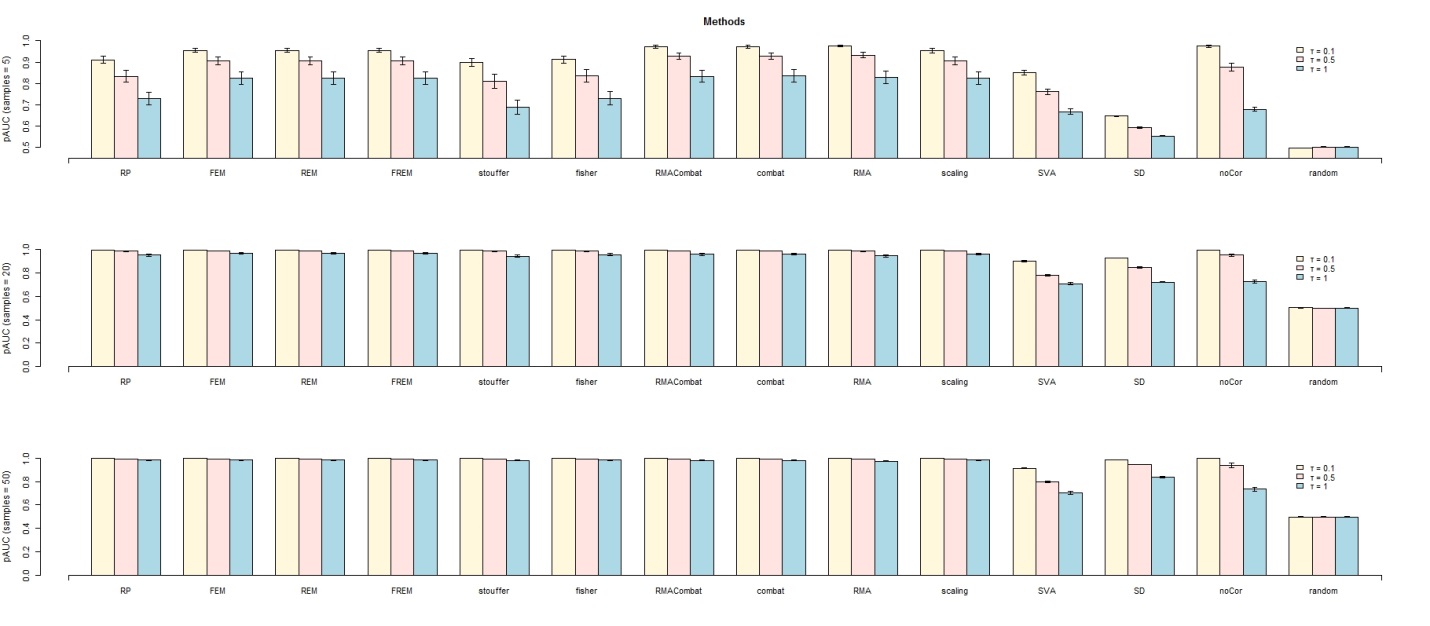


Figure S 11: pAUC results on simulated data for different sample sizes using Pearson Correlations.


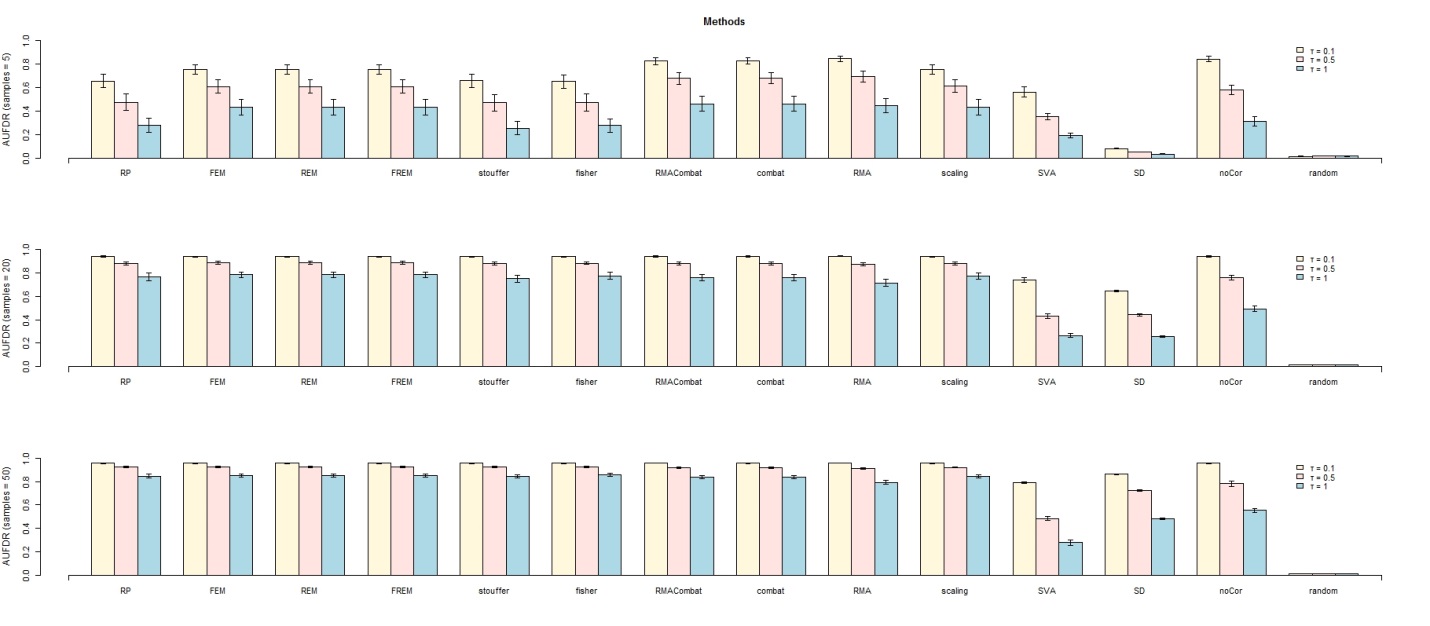


Figure S 12: AUFDR results on simulated data for different sample sizes using Pearson Correlations.


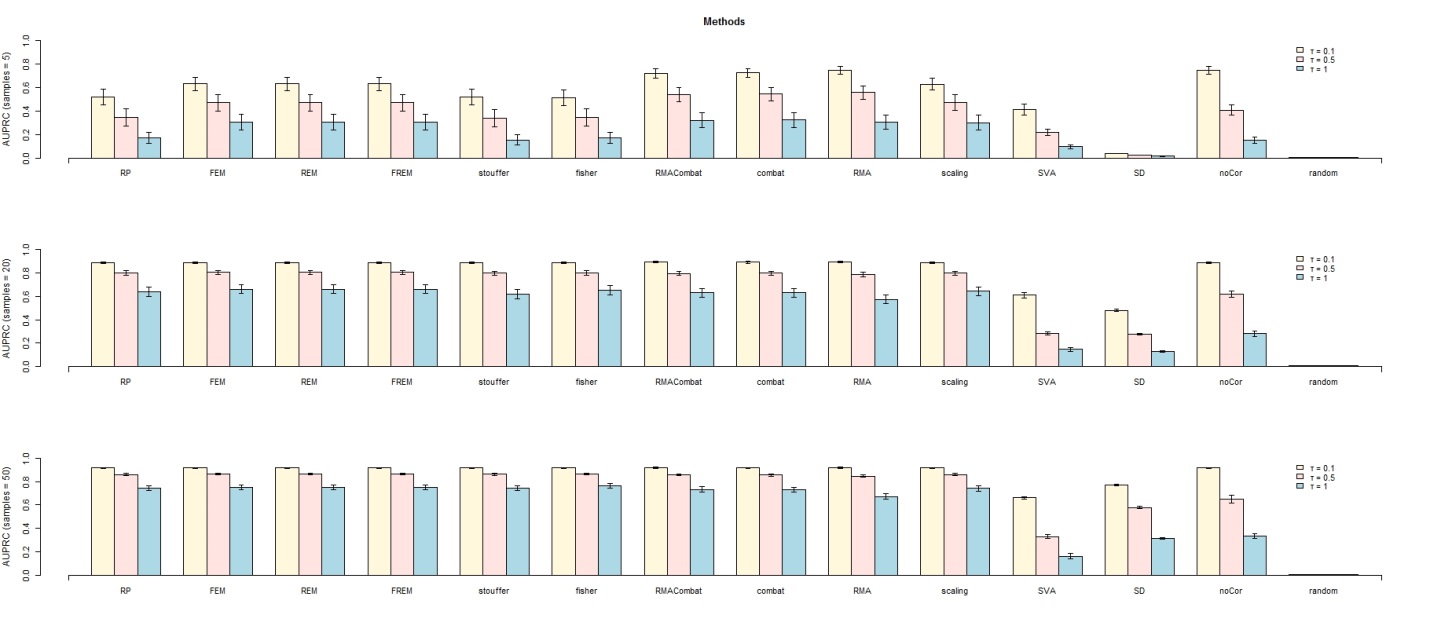


Figure S 13: AUPRC results on simulated data for different sample sizes using Pearson Correlations.


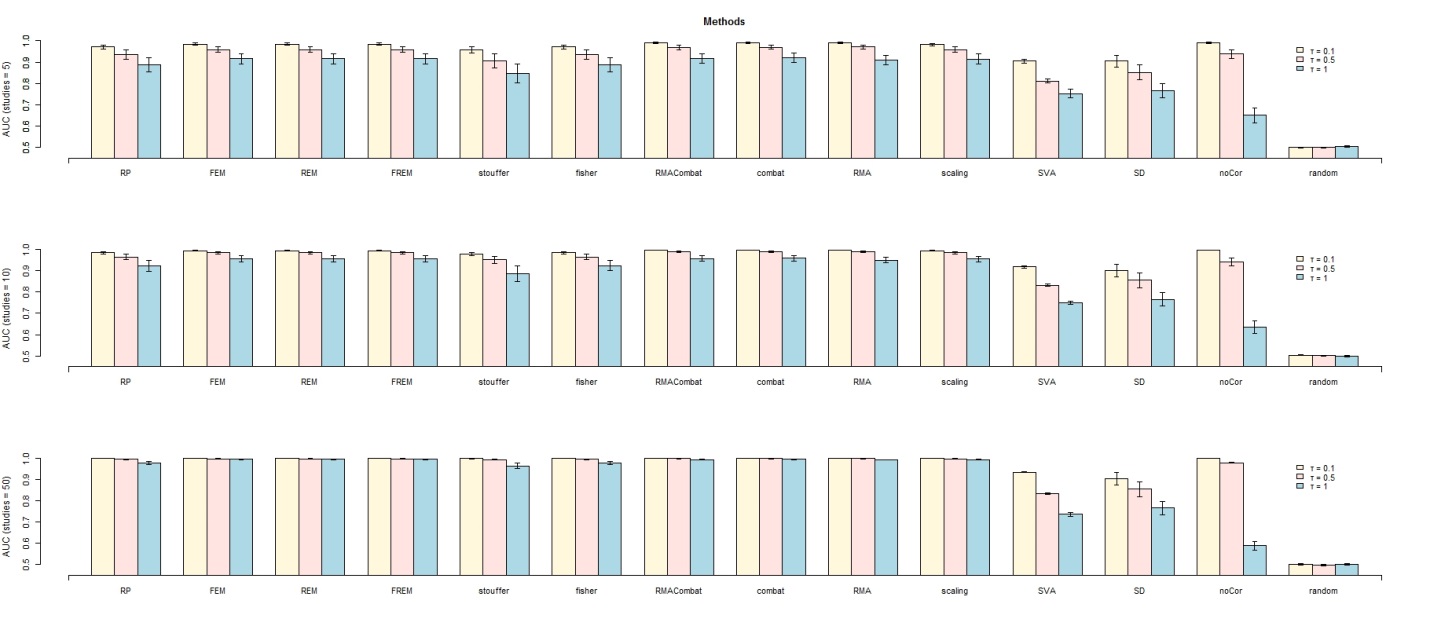


Figure S 14: AUC results on simulated data across different number of studies using Pearson Correlations


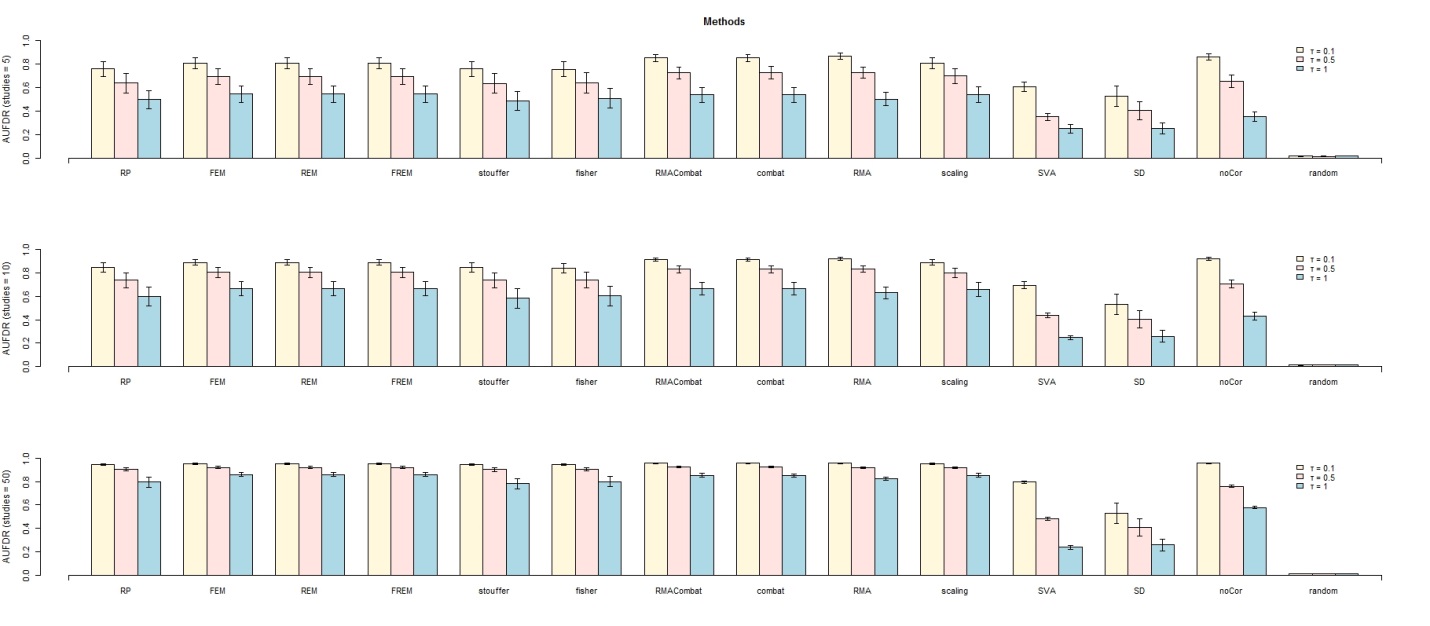


Figure S 15: AUFDR results on simulated data across different number of studies using Pearson Correlations


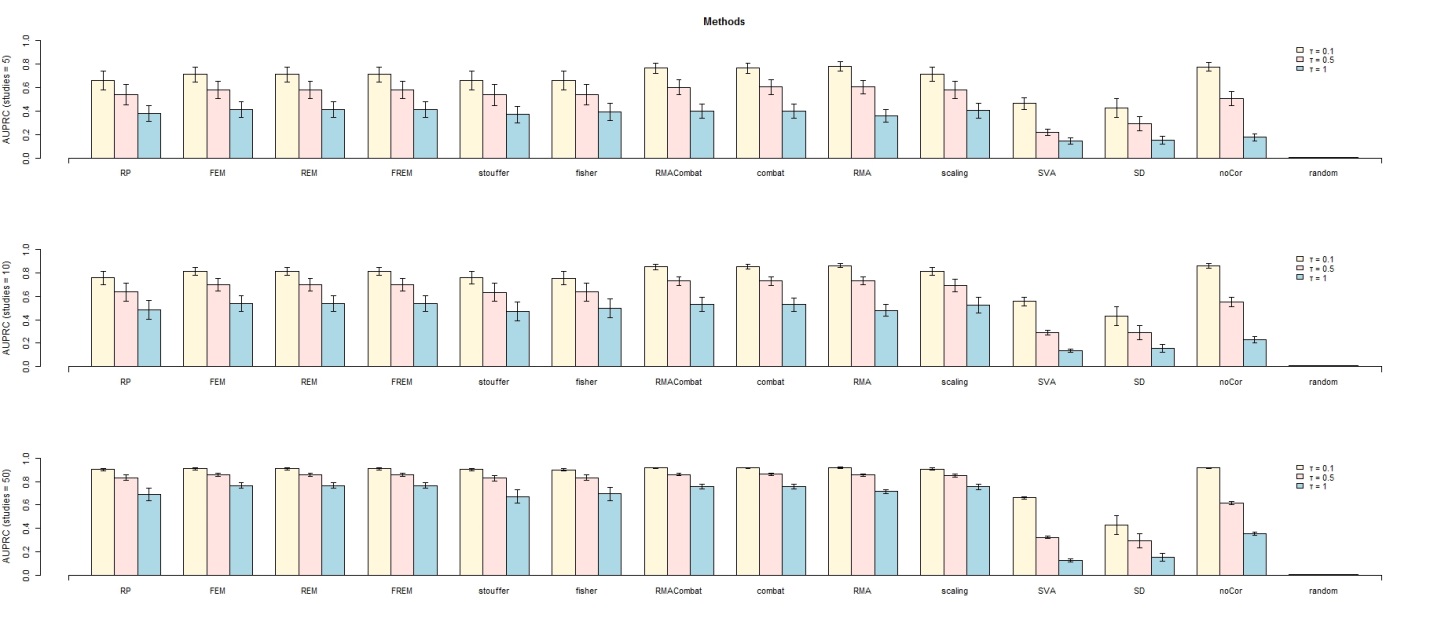


Figure S 16: AUPRC results on simulated data across different number of studies using Pearson Correlations


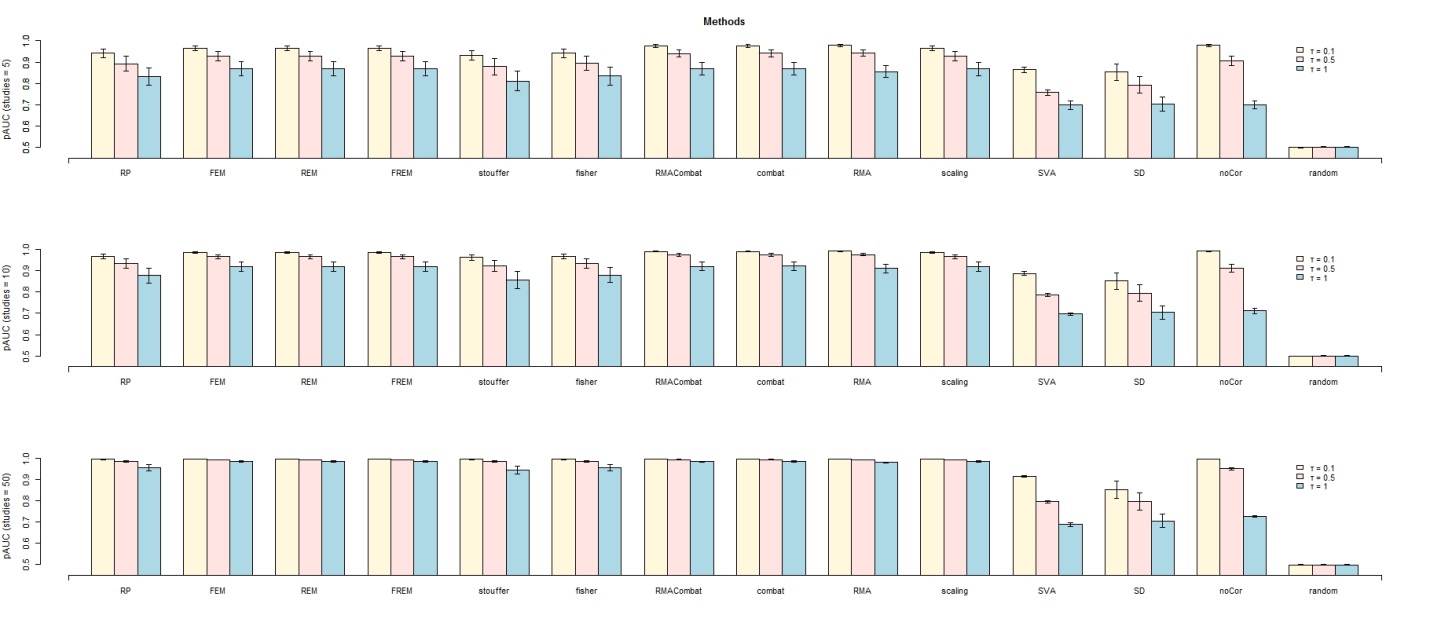


Figure S 17: pAUC results on simulated data across different number of studies using Pearson Correlations


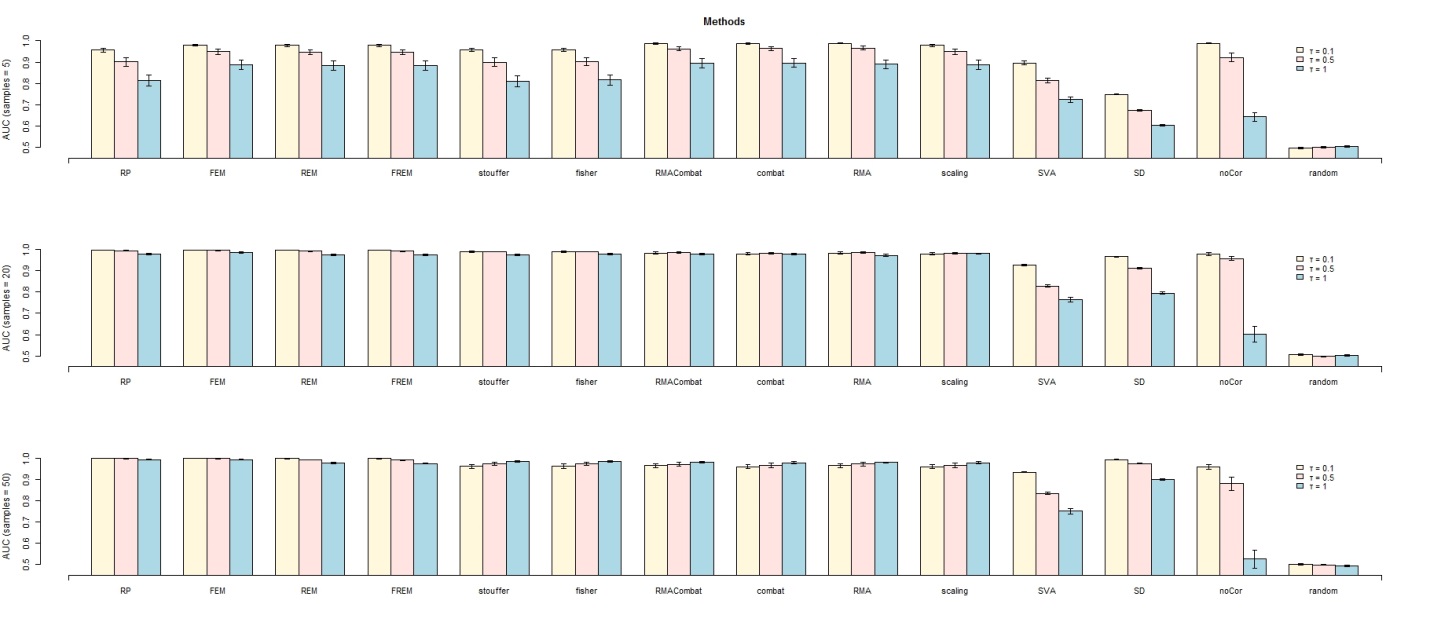


Figure S 18: AUC results on simulated data across different number of samples using Pearson p-values


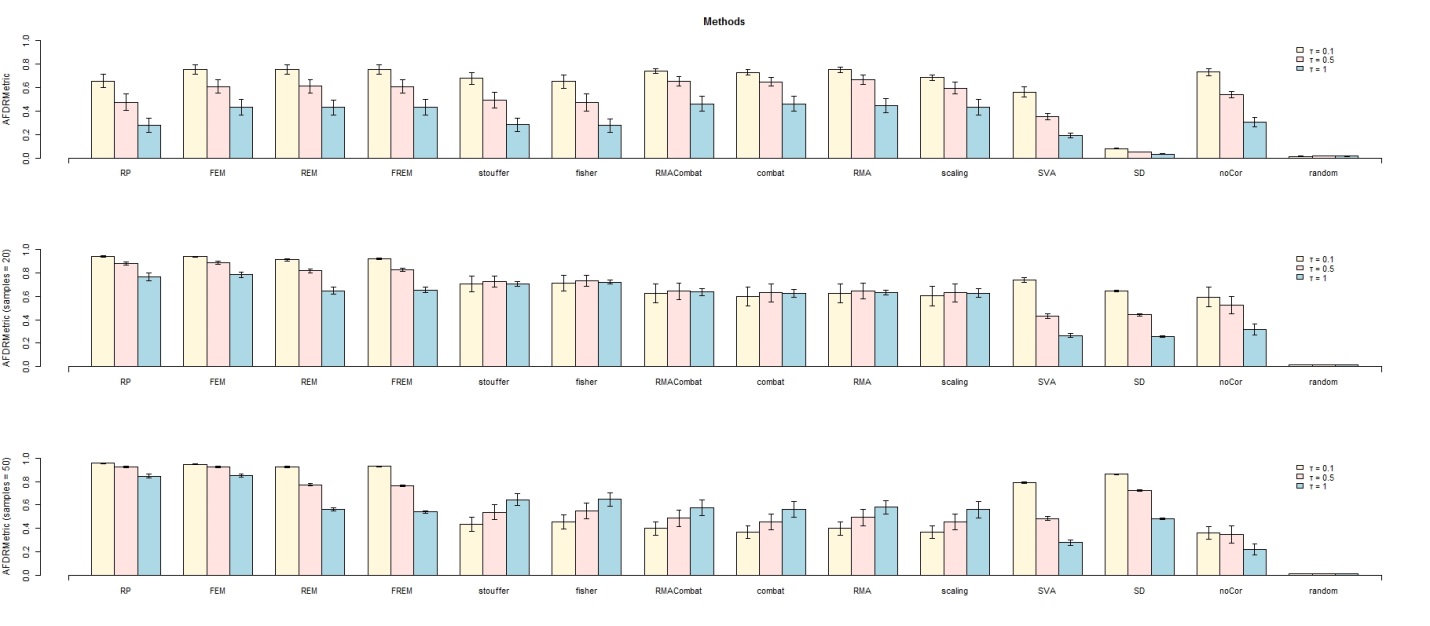


Figure S 19: AUFDR results on simulated data across different number of samples using Pearson p-values


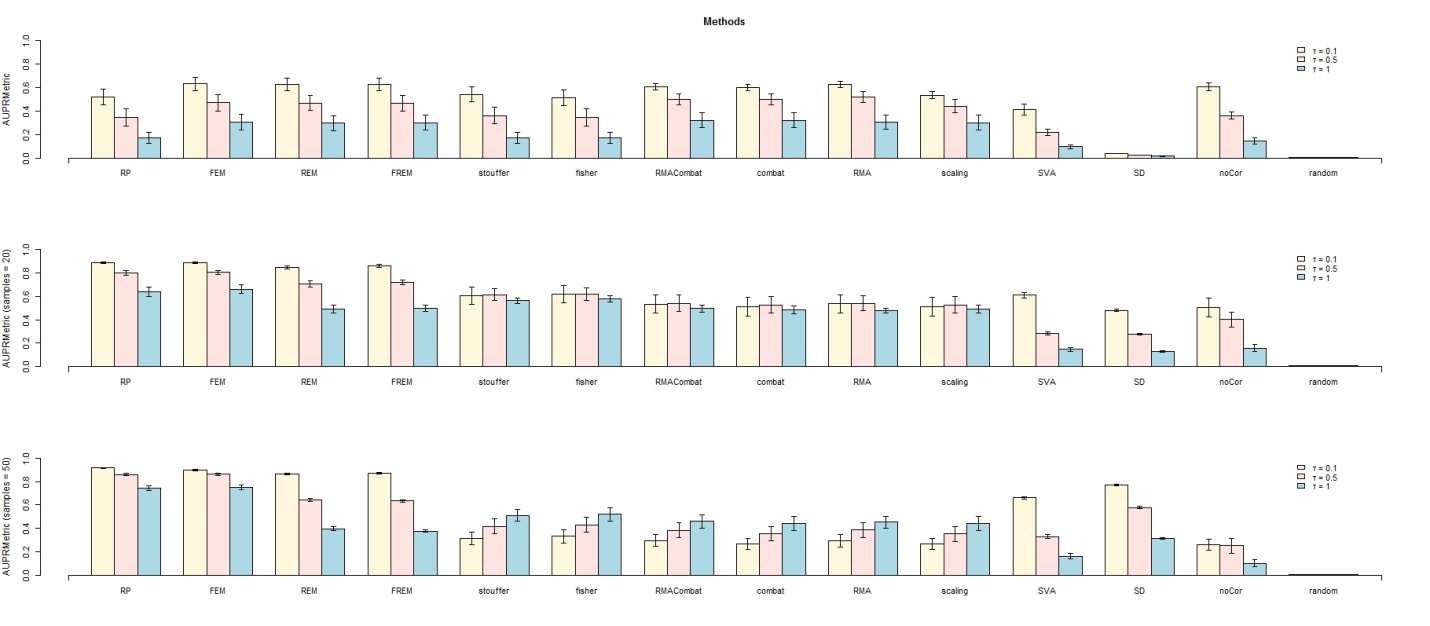


Figure S 20: AUPRC results on simulated data across different number of samples using Pearson p-values


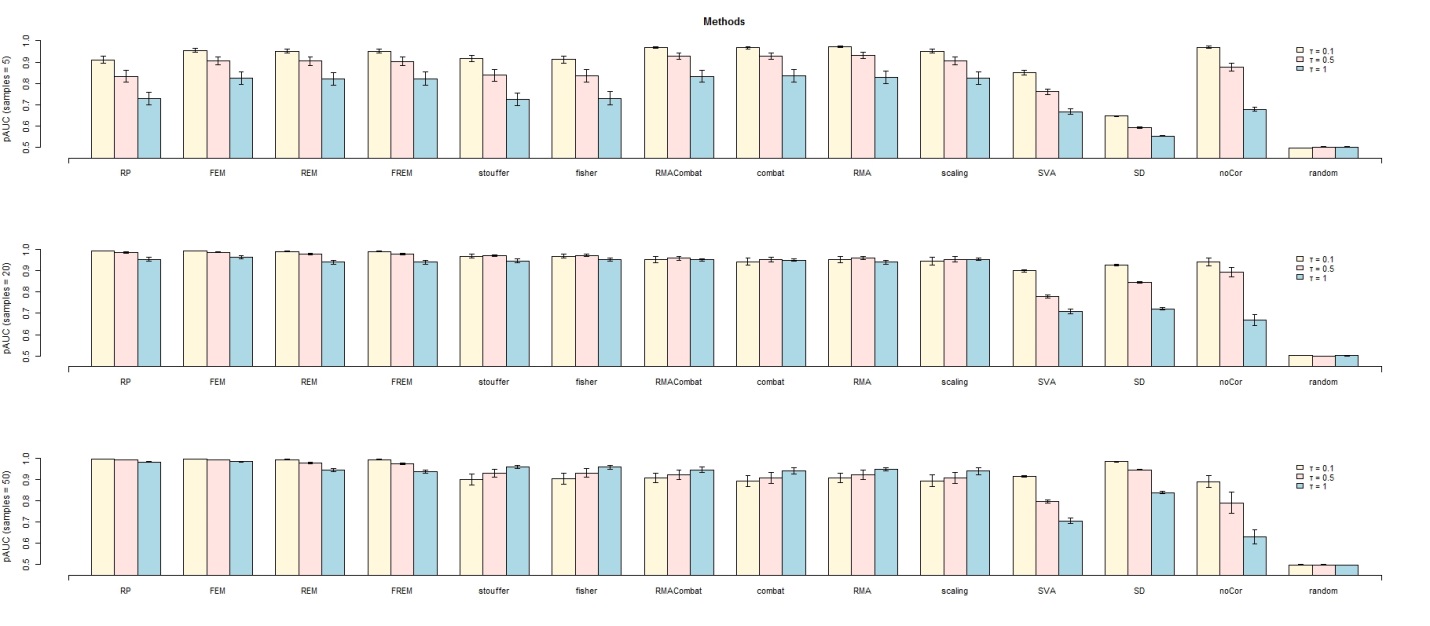


Figure S 21: pAUC results on simulated data across different number of samples using Pearson p-values


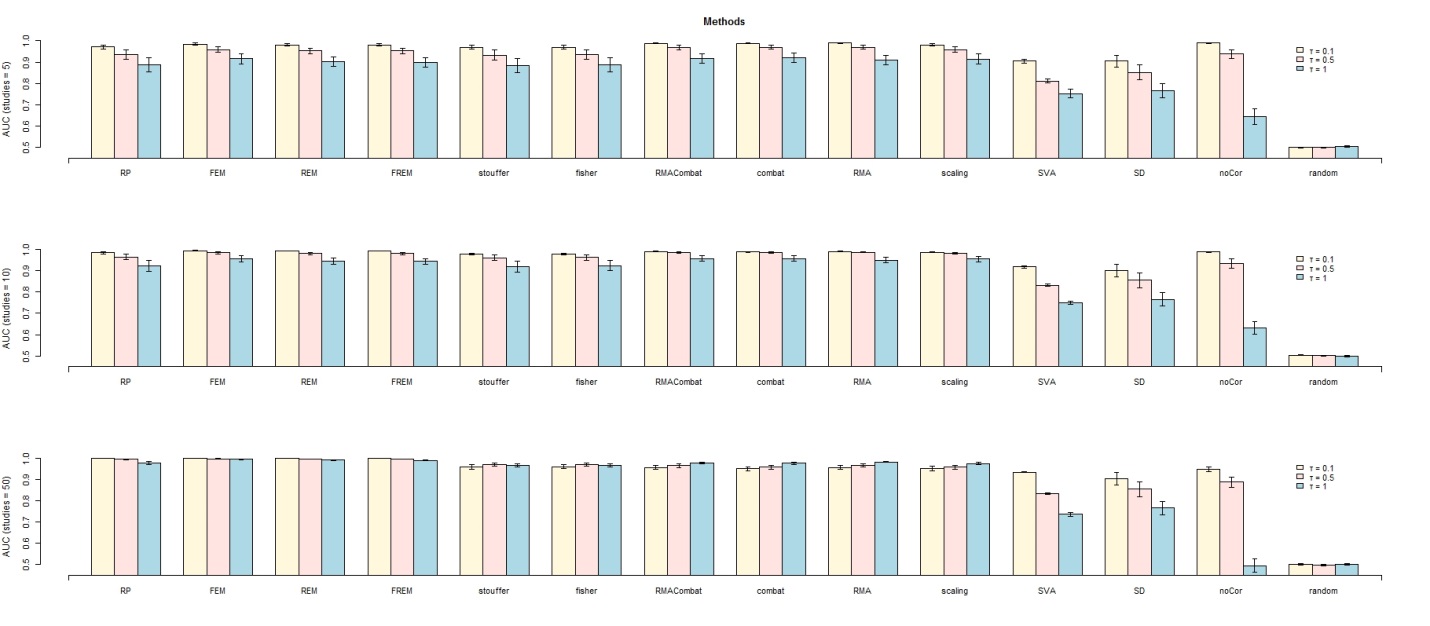


Figure S 22: AUC results on simulated data across different number of studies using Pearson p-values


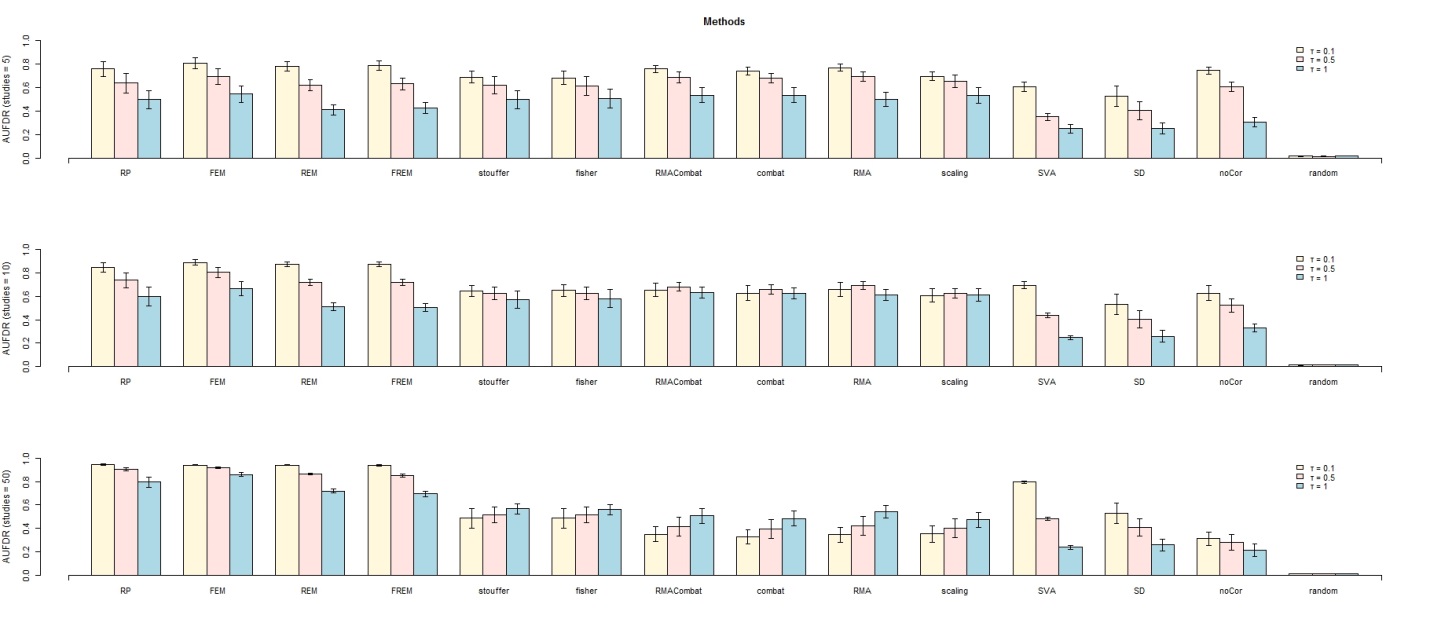


Figure S 23: AUFDR results on simulated data across different number of studies using Pearson p-values


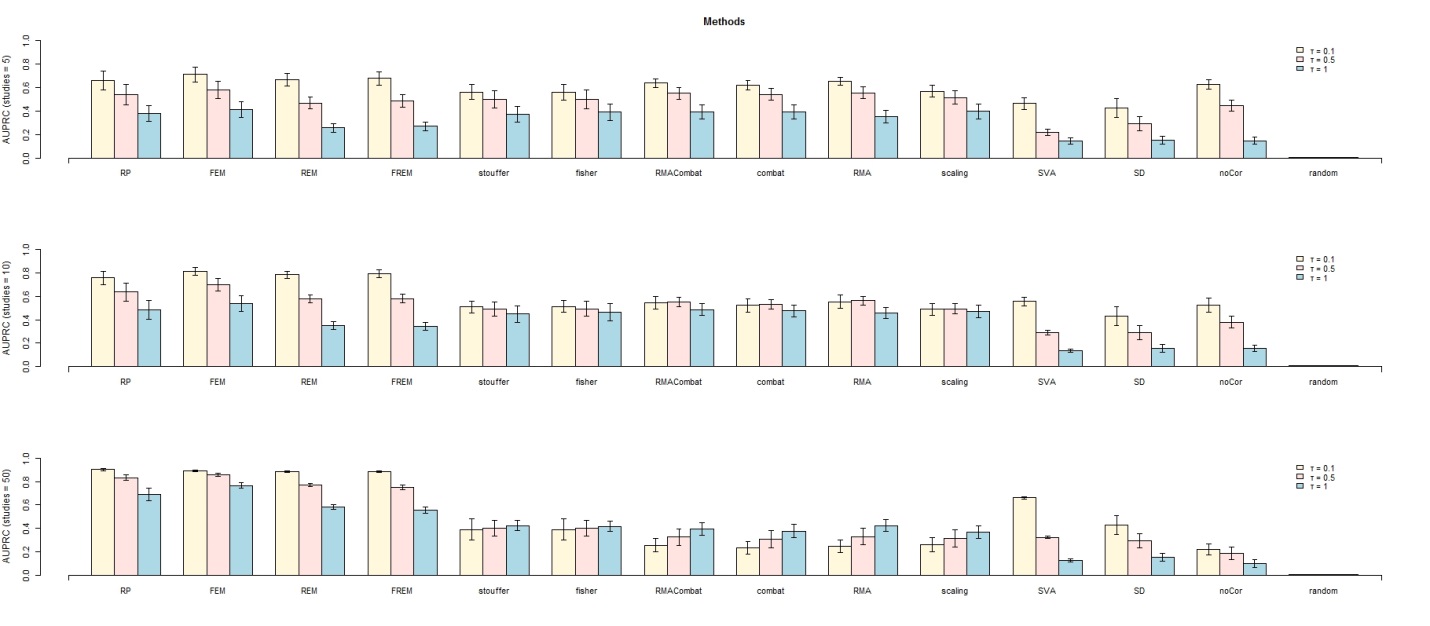


Figure S 24: AUPRC results on simulated data across different number of studies using Pearson p-values


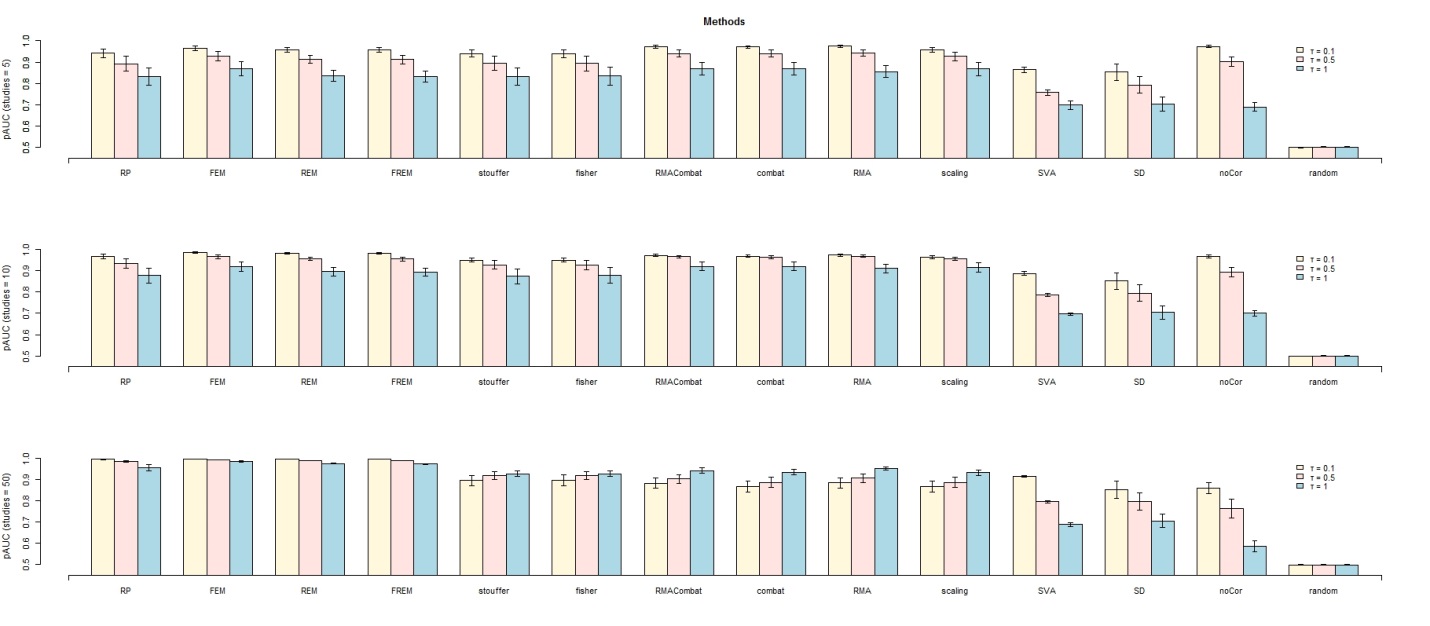


Figure S 25: pAUC results on simulated data across different number of studies using Pearson p-values


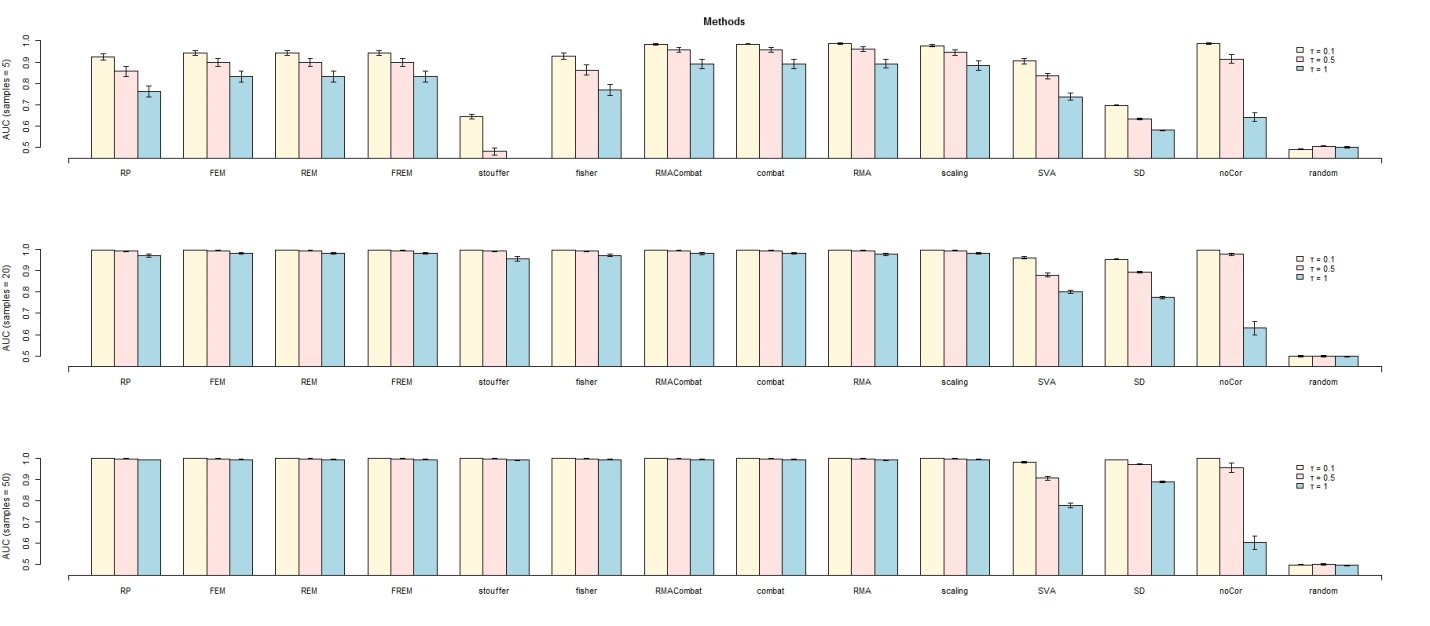


Figure S 26: AUC results on simulated data across different sample sizes using Spearman Correlation


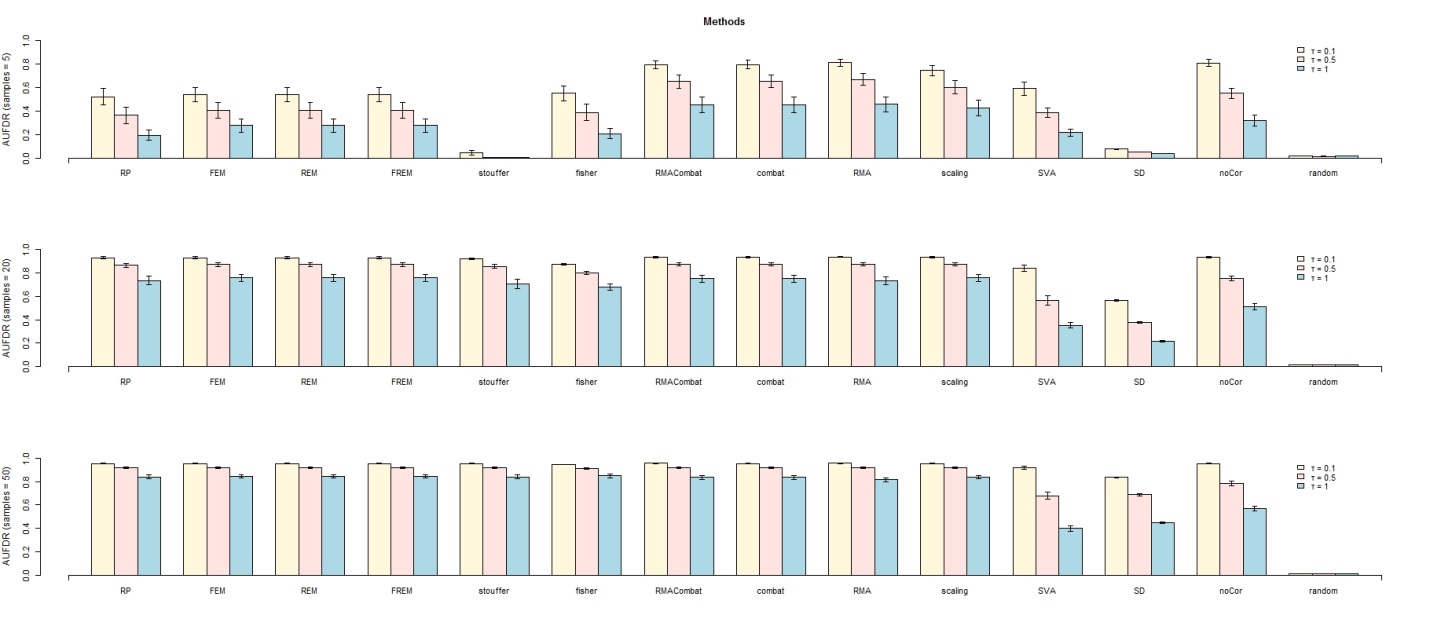


Figure S 27: AUFDR results on simulated data across different sample sizes using Spearman Correlation


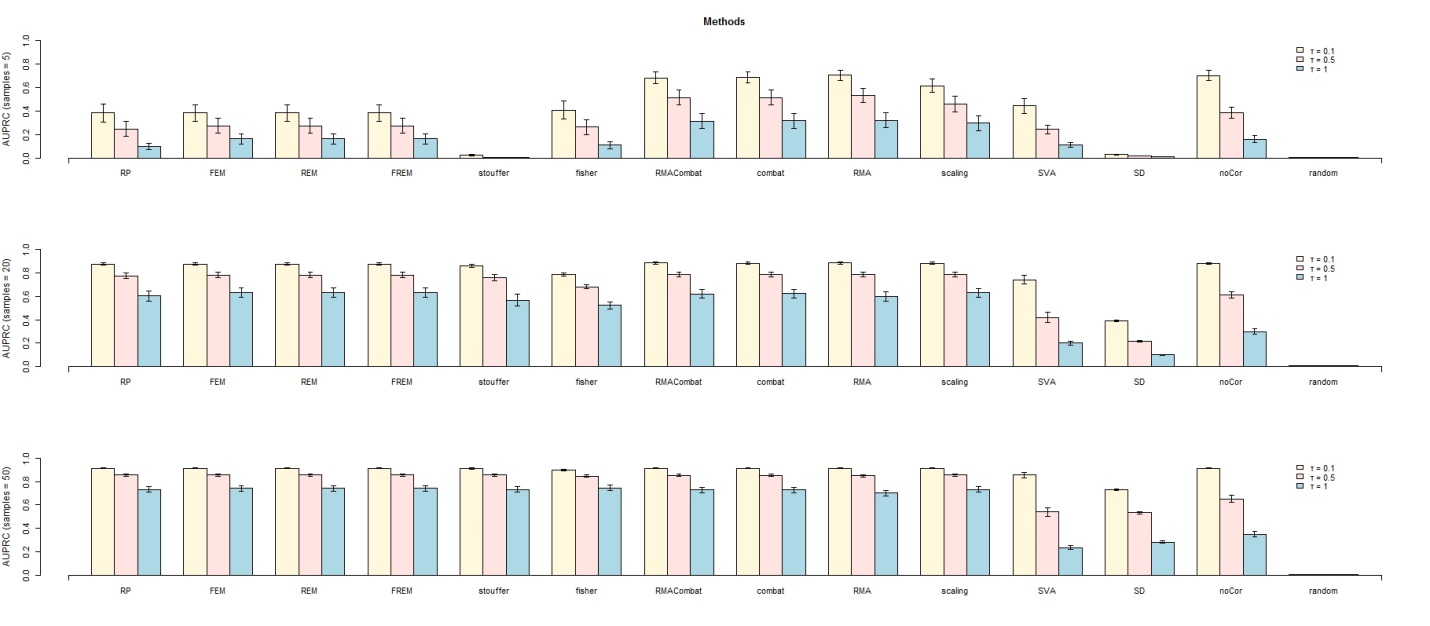


Figure S 28: AUPRC results on simulated data across different sample sizes using Spearman Correlation


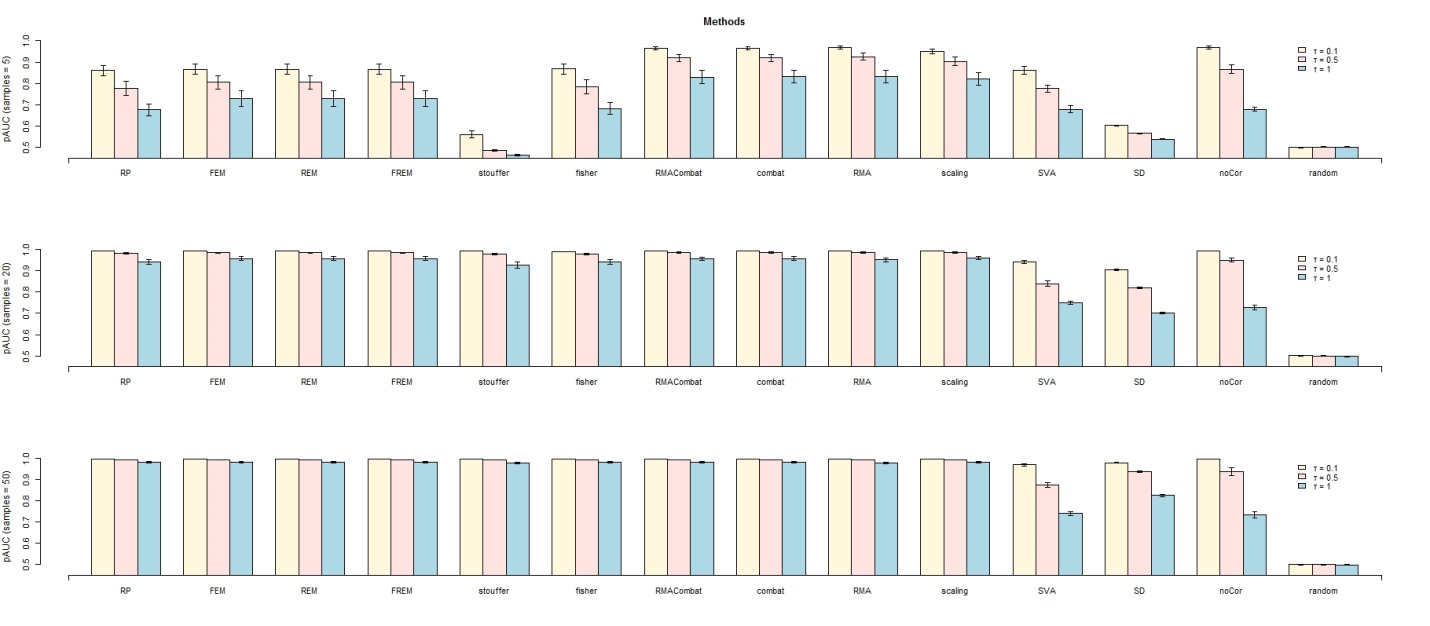


Figure S 29: pAUC results on simulated data across different sample sizes using Spearman Correlation


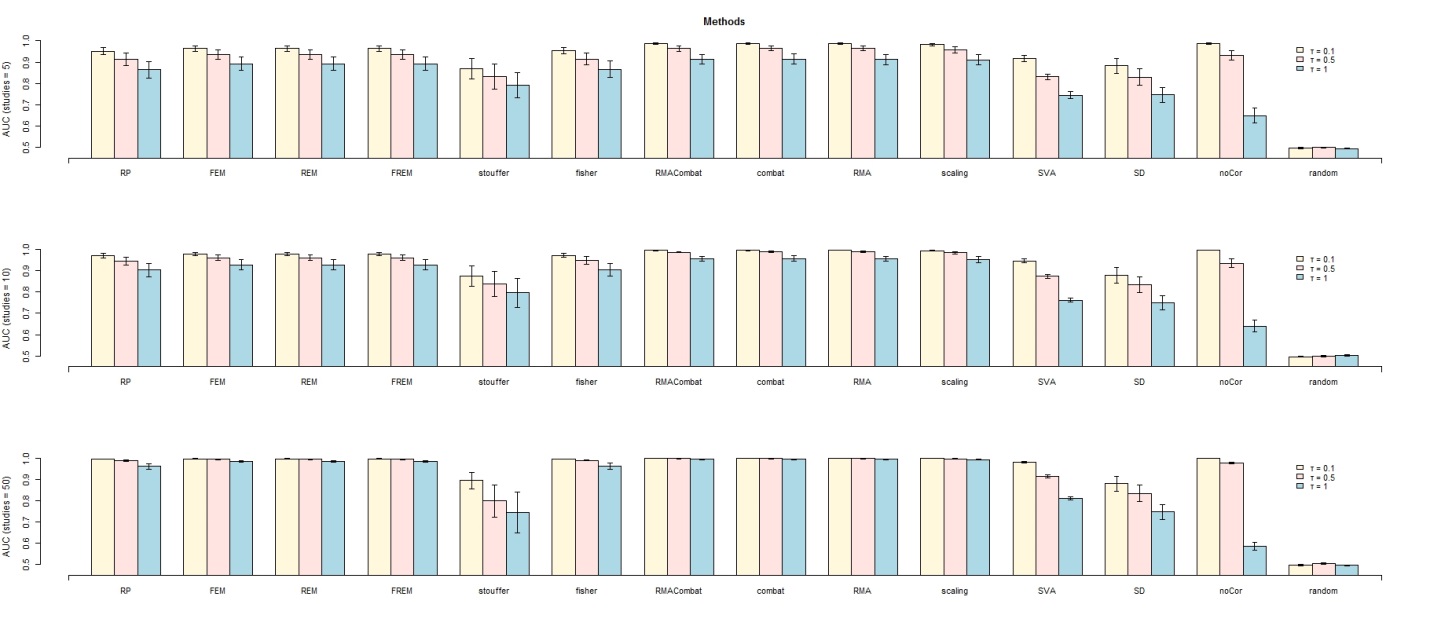


Figure S 30: AUC results on simulated data across different number of studies using Spearman Correlation


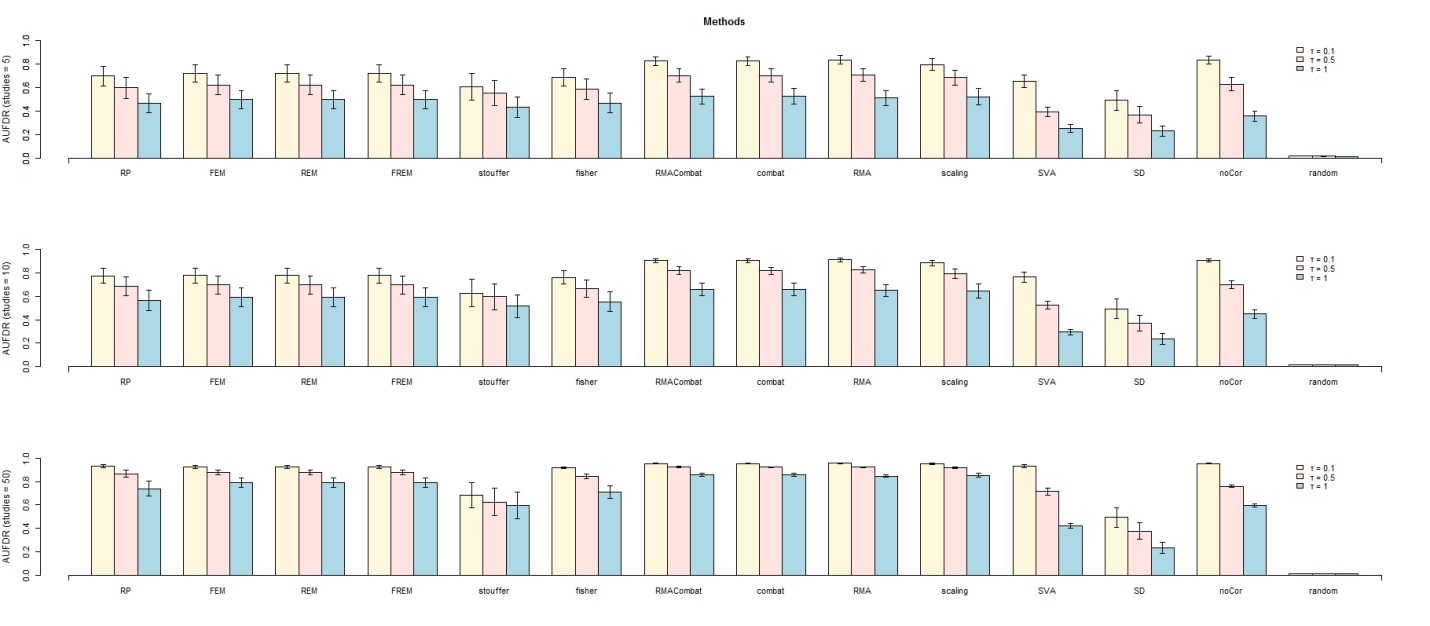


Figure S 31: AUFDR results on simulated data across different number of studies using Spearman Correlation


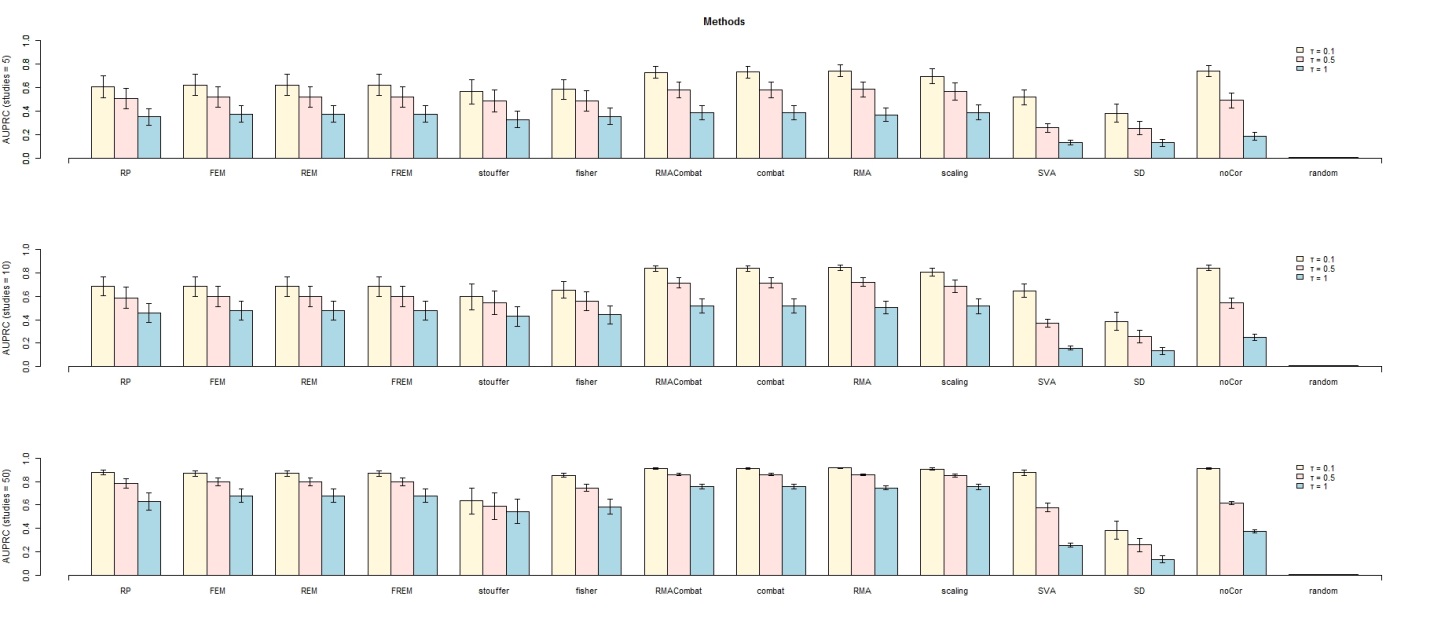


Figure S 32: AUPRC results on simulated data across different number of studies using Spearman Correlation


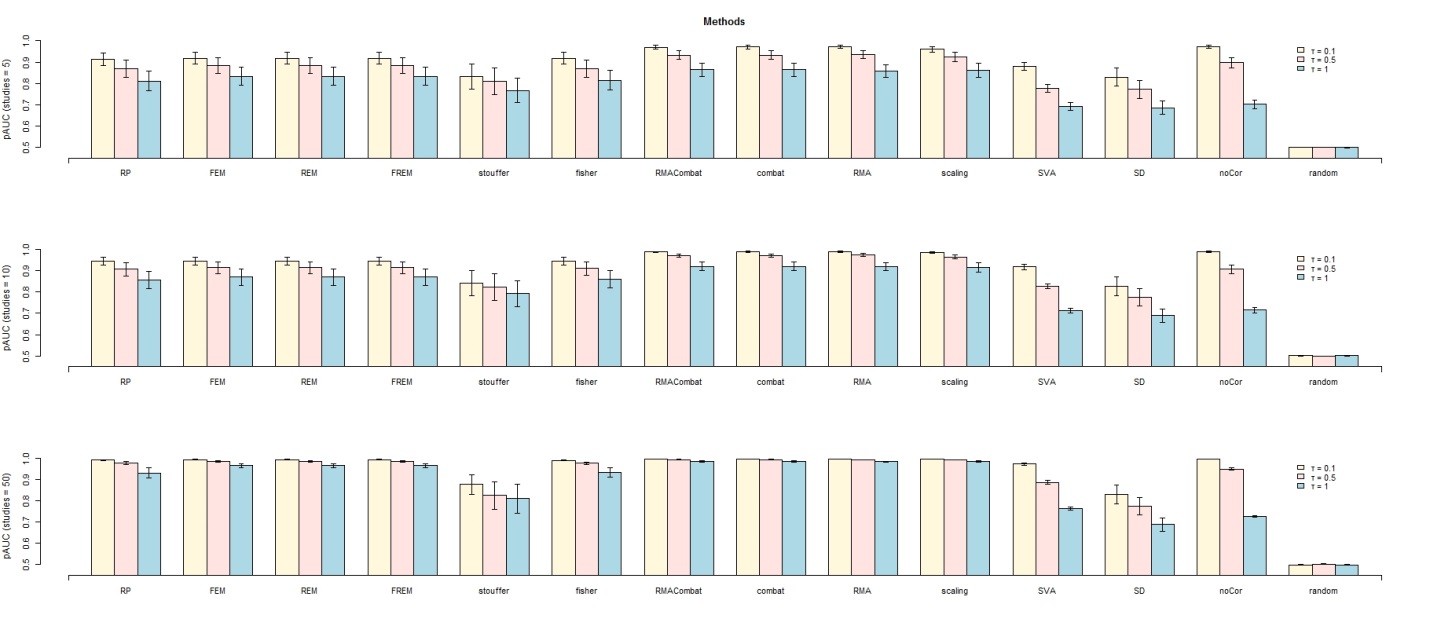


Figure S 33: pAUC results on simulated data across different number of studies using Spearman Correlation


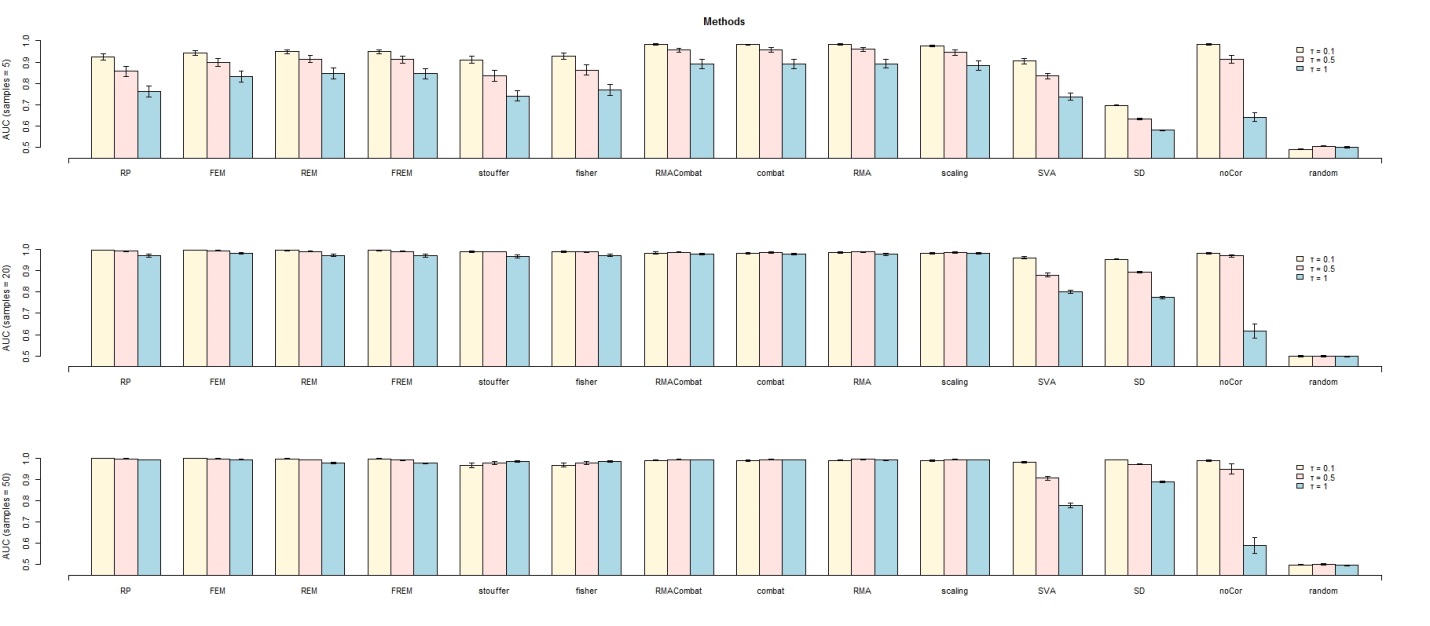


Figure S 34: AUC results on simulated data across different sample sizes using Spearman Correlation p-values.


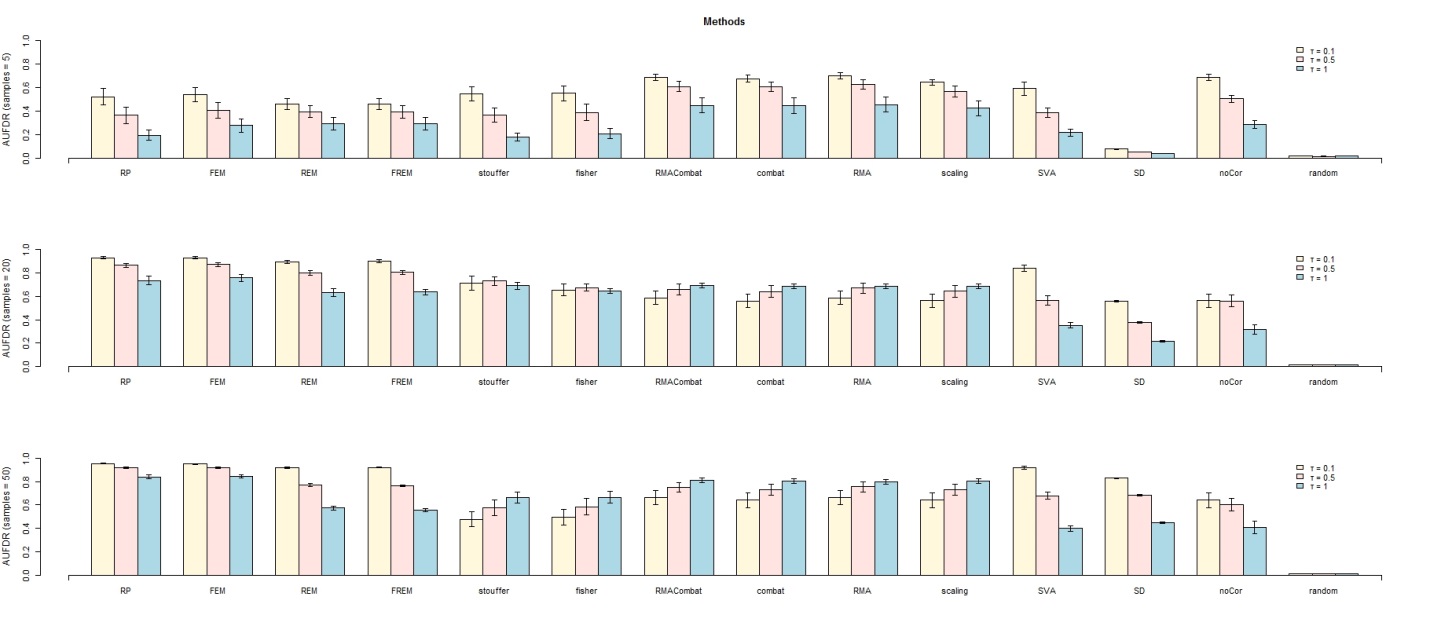


Figure S 35: AUFDR results on simulated data across different sample sizes using Spearman Correlation p-values.


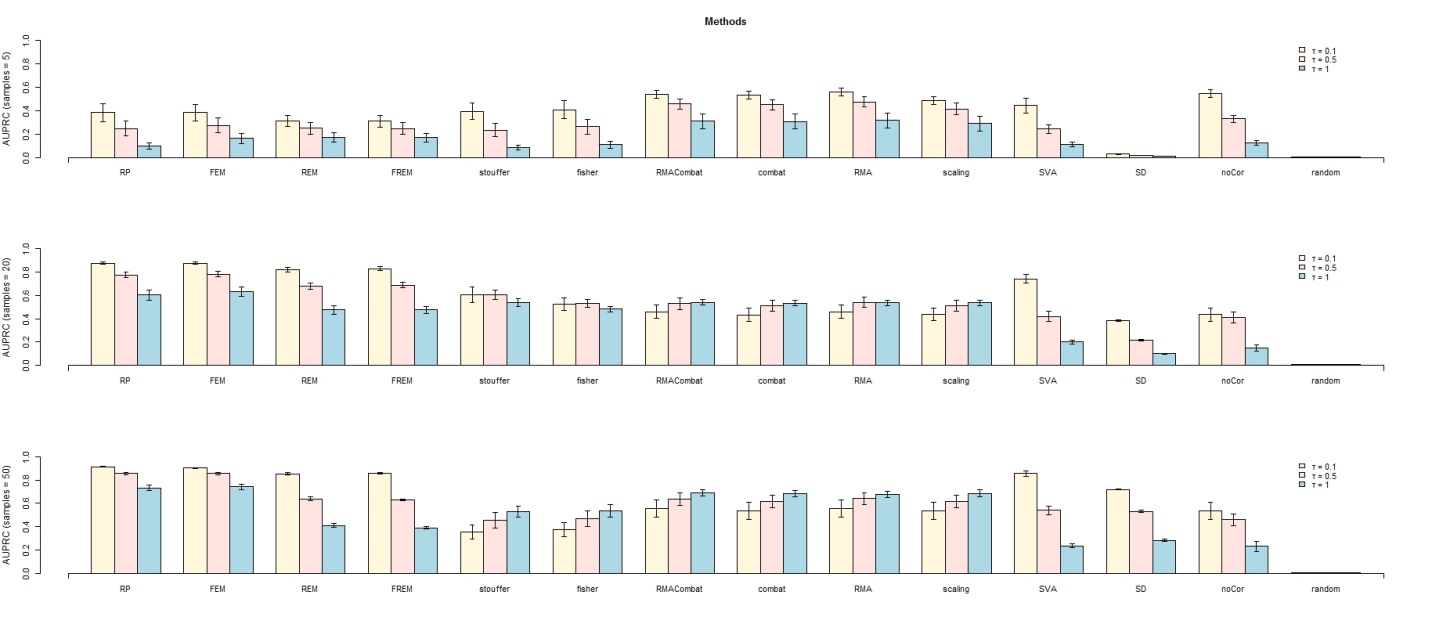


Figure S 36: AUPRC results on simulated data across different sample sizes using Spearman Correlation p-values.


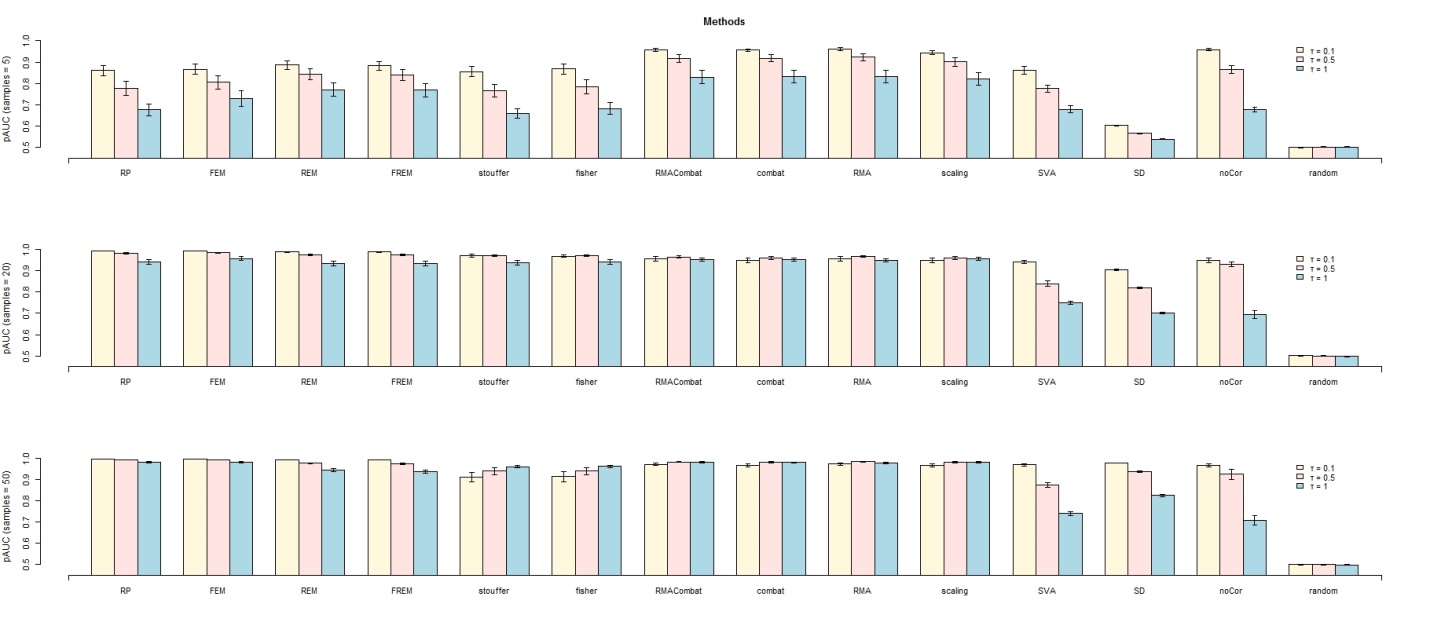


Figure S 37: pAUC results on simulated data across different sample sizes using Spearman Correlation p-values.


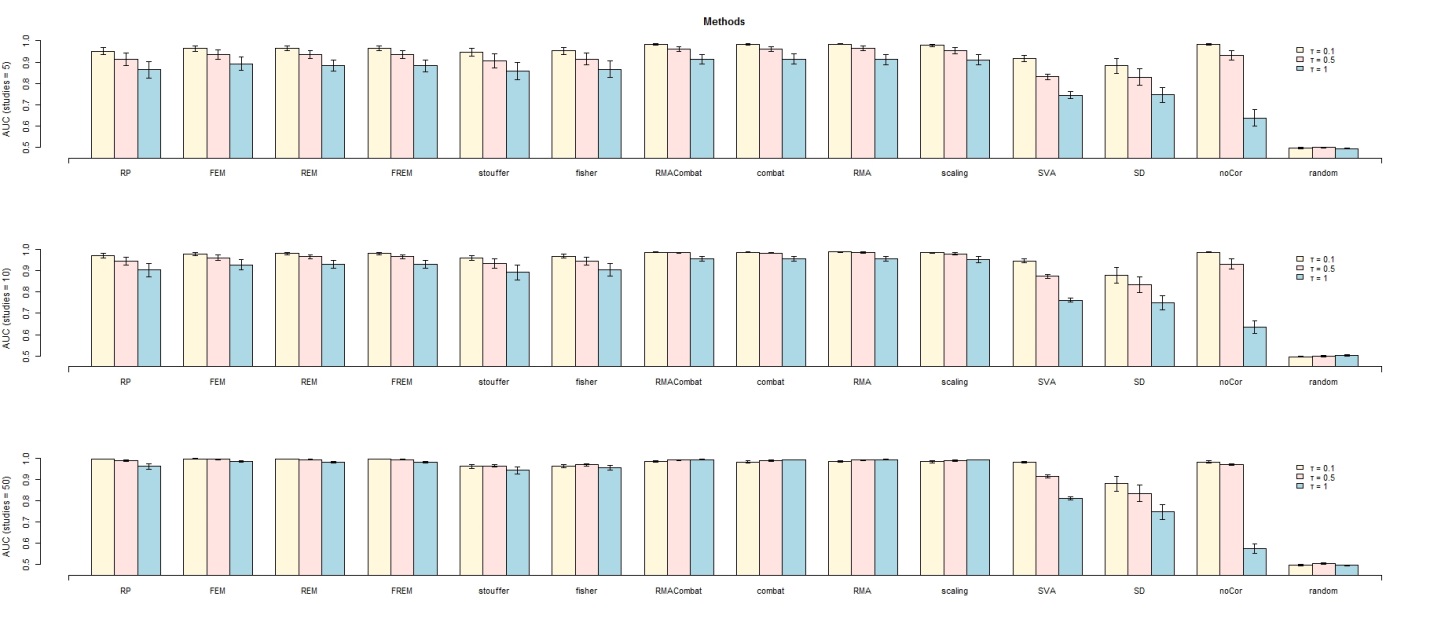


Figure S 38: AUC results on simulated data across different number of studies using Spearman Correlation p-values.


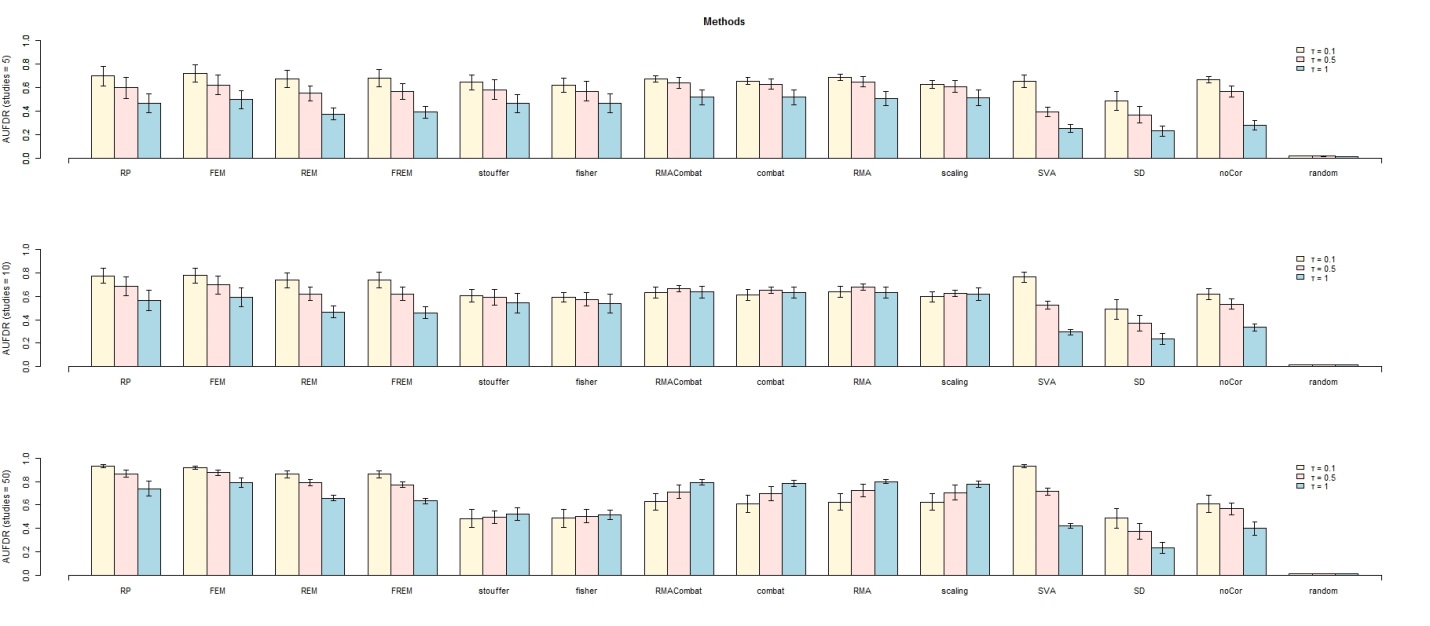


Figure S 39 AUFDR results on simulated data across different number of studies using Spearman Correlation p-value


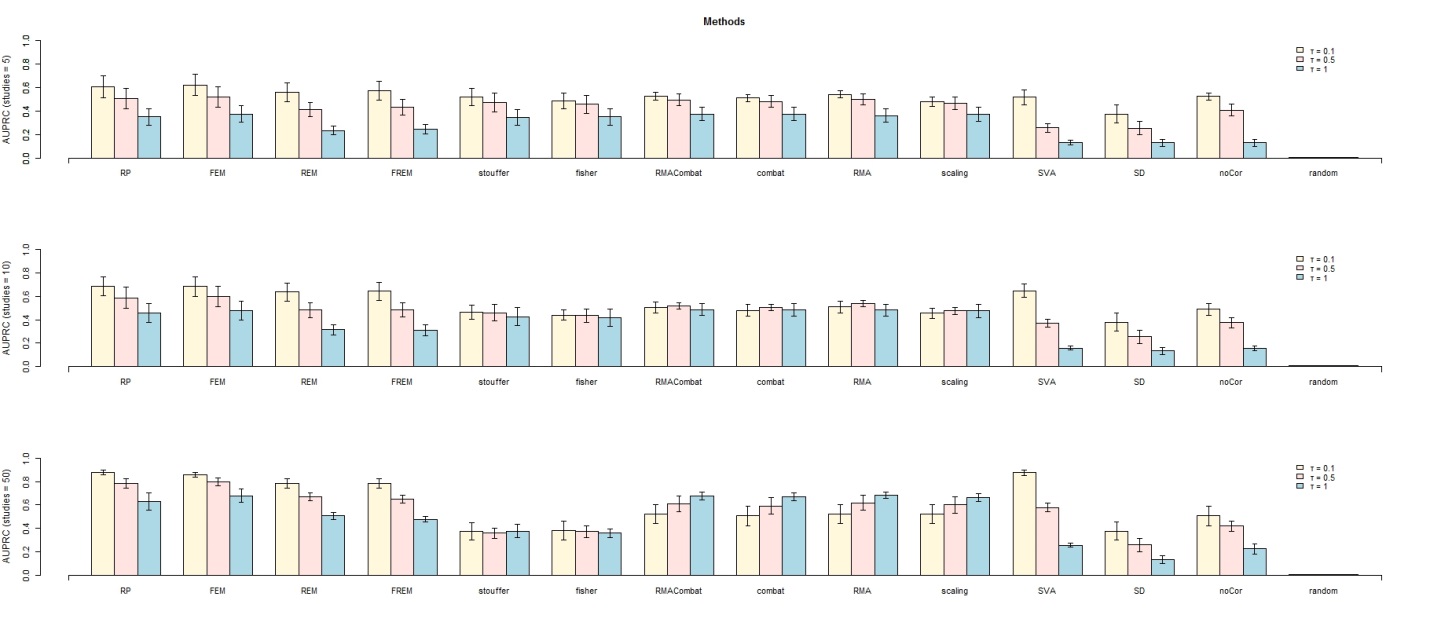


Figure S 40 AUPRC results on simulated data across different number of studies using Spearman Correlation p-value


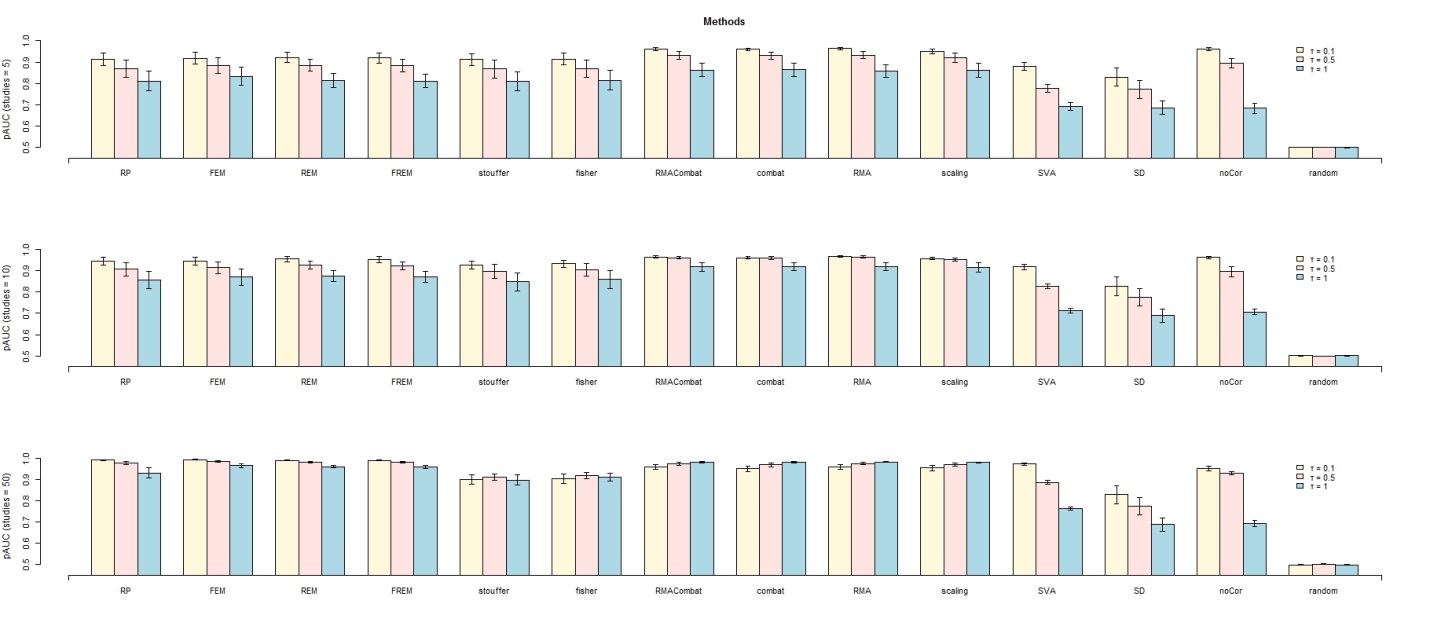


Figure S 41 pAUC results on simulated data across different number of studies using Spearman Correlation p-value
