# Supplementary material for: A comparative evaluation of data-merging and meta-analysis methods for reconstructing gene-gene interactions
Source: BMC Bioinformatics. 2016 Jun 6;17(Suppl 5):194. doi: 10.1186/s12859-016-1038-1 (PMC4905611; doi:10.1186/s12859-016-1038-1)
Supplement: Supplementary file 2 — Using Surrogate Variable Analysis for Network Reconstruction. This additional file presents a sub-study investigating modifications of the SVA model that allow to use the SVA method on network reconstruction tasks. (DOCX 137 kb) [file 12859_2016_1038_MOESM2_ESM.docx]

Using Surrogate Variable Analysis for network reconstruction.

Sub-study for the manuscript “Lagani et al. A comparative evaluation of data-merging and meta-analysis methods for reconstructing gene-gene interactions. BMC Bioinformatics.”

The Surrogate Variable Analysis (SVA) approach is a statistical technique that attempts to estimate and adjusts for the effect of undesired batch-effects and confounding factors. Here we propose two modifications for adapting this approach to network reconstruction tasks, particularly for identifying gene-gene interactions from expression data using relevancy networks. Furthermore, we evaluate the proposed modifications on the E. Coli and Yeast data collections presented in the main manuscript. The results seem to indicate that, if opportunely employed, the SVA method produces performances that matches the results of the other batch-effect removal methods.

# The Surrogate Variable Analysis (SVA) method.

The SVA approach introduced by Leek and Storey [1] attempts to identify and remove all confounding factors negatively affecting the analysis, including eventual batch-effects. In the common case-control scenario, the *SVA model* is the following:$y_{sj}=\alpha_{j}+\beta_{j}x_{s}+\sum_{k} \gamma_{jk}g_{ks}+\epsilon_{sj}$, where $y_{sj}$is the expression of gene $j$ in sample $s$, $a_{j}$ is the overall gene expression of $j$, $x_{s}$ is a binary variable indicating whether sample $s$ is a case or a control, $\beta_{j}$represents the average difference in expression between the two conditions in gene $j$, and $\epsilon_{sj}$ is a random error. The term $\sum_{k} \gamma_{jk}g_{ks}$ represents the cumulative effect on $y_{sj}$ of $K$ unknown confounding factors $g_{ks}$, multiplied by their gene-specific coefficients $\gamma_{jk}$. SVA attempts to estimate confounding factors’ global effect by deriving a set of surrogate variables $\boldsymbol{h}_{1}, \boldsymbol{h}_{2}, \ldots,\boldsymbol{h}_{K}$ whose span covers the same linear space spanned by the vectors $\boldsymbol{g}_{k}$. The estimation is based on the Singular Value Decomposition (SVD) of the residual matrix obtained after fitting the SVA model on the expression data. The estimated surrogate variables are meant to be used as covariates in all subsequent analysis in order to rule out the effect of the unknown confounding factors.

One of the main issues in using SVA is the estimation of $K$, the number of surrogate variables needed for accurately approximate the effect of the vectors $\boldsymbol{g}_{k}$. The original SVA publication [1] uses a permutation-based method, initially described by Buja and Eyuboglu [2] (‘BE’ method, for sake of conciseness). Successively, Leek proposed a different approach for estimating $K$ (‘Leek’ method), based on the asymptotic properties of a conditional factor model for genomic data [3].

In a recent publication Gagnon-Bartsch and Speed [4] suggested that batch-effects should be estimated solely from control genes, i.e., those genes whose expression is not expected to vary across the experimental conditions investigated in the study. Any systematic variation in these genes should be due to batch-effects or other unknown confounding factors. The Supervised SVA (SSVA) employs this principle and estimates the surrogate variables from control genes only [5].

# Adapting SVA for network reconstruction tasks

To the best of our knowledge, no previous study applied SVA on network reconstruction tasks. We propose two modifications for adapting the SVA model to the particular scenario in which the local regulatory network of the transcription factor $\mathrm{TF}_{i}$ must be reconstructed from gene expression data:

- **Modification 1:** gene-regulatory networks usually have a scale-free topology, meaning that the large majority of the genes interacts only with few others [6]. It is thus reasonable to assume that each $\mathrm{TF}_{i}$ has a significant effect only on a restricted subset of genes, and that all major systematic variations present in the data should be due to experimental factors, batch-effects or confounding factors. Consequently, for the data collections used in this study the SVA model becomes: $y_{sj}=\alpha_{j}+\sum_{k} \gamma_{jk}g_{ks}+\epsilon_{sj}$. From a computational perspective this formulation is equivalent to estimate the surrogate variables by applying SVD to the original data matrix after having centered each gene around its mean.
- **Modification 2:** if it is reasonable to assume that $\mathrm{TF}_{i}$ has a relevant effect on a large portion of genes, it may be preferable to estimate the surrogate variables on the residuals left after subtracting the effect of $TF_{i}$ from all other genes. The SVA model should then be modified as follows: $y_{sj}=\alpha_{j}+\mathrm{TF}_{si}+\sum_{k} \gamma_{jk}g_{ks}+\epsilon_{sj}$, where $\mathrm{TF}_{si}$ is the expression value of transcription factor $\mathrm{TF}_{i}$ in sample $s$. Two interesting notes about this formulation: (a) since we focus on the local regulatory network of $\mathrm{TF}_{i}$, any regulatory mechanism not directly involving $\mathrm{TF}_{i}$ can be thought as a confounding factor; (b) in this setting a separate set of surrogate variables must be estimated for each transcription factor.

Since it is not simple to decide a priori which modification should provide the best results, we contrast them over the E. Coli and Yeast data collections described in the main manuscript. Unfortunately, along with deciding which modification to use, there are a few more algorithmic choices that must be taken in order to apply SVA on gene-network reconstruction tasks:

1. **Method for computing** $\boldsymbol{K}$: both modifications above can be paired with either the BE or Leek method for computing the number $K$ of surrogate variables.
2. **Initial data matrix**: SVA can be applied alone or in conjunction with other batch-effect removal methods. In the first case, SVA is applied on the dataset obtained by pooling together the data from each single study (i.e., the data matrix resulting from the ‘No-correction’ approach). In the latter case, SVA can be applied to the dataset provided by any other batch-effect removal method. We chose the RMA-Combat method for our experimentations.
3. **Gene set**: SVA can be applied on either the whole set of genes or on the control genes only. In our experimentations we identify the latter with the Affymetrix “control probesets”. These probesets are designed in order to match RNA sequences that are not supposed to be produced by the organism targeted by the specific Affymetrix array. The expression levels detected by these probeset should be due exclusively to random noise or technological artifacts, for example batch-effects.

We devised several versions of “network-reconstruction SVA”, corresponding to all possible configurations given by the above factors. Table 1 summarizes all 16 versions (SVA-1 to SVA-16).

Table 1: “Network-Reconstruction SVA” versions that can be implemented according to the specific algorithmic choices. The versions are progressively numbered from SVA-1 to SVA-16, and differ according to their respective SVA model modification, method for computing the number K of surrogate variables, the initial data matrix provided to SVA as input, and the gene set used for estimating the surrogate variables. See the text for detailed descriptions.

| SVA version | SVA model modification | Method for computing K | Initial Data Matrix | Gene set |
| --- | --- | --- | --- | --- |
| SVA-1 | 1 | BE | No-correction | All probesets |
| SVA-2 | 1 | BE | No-correction | Control probesets |
| SVA-3 | 1 | BE | RMA-Combat | All probesets |
| SVA-4 | 1 | BE | RMA-Combat | Control probesets |
| SVA-5 | 1 | Leek | No-correction | All probesets |
| SVA-6 | 1 | Leek | No-correction | Control probesets |
| SVA-7 | 1 | Leek | RMA-Combat | All probesets |
| SVA-8 | 1 | Leek | RMA-Combat | Control probesets |
| SVA-9 | 2 | BE | No-correction | All probesets |
| SVA-10 | 2 | BE | No-correction | Control probesets |
| SVA-11 | 2 | BE | RMA-Combat | All probesets |
| SVA-12 | 2 | BE | RMA-Combat | Control probesets |
| SVA-13 | 2 | Leek | No-correction | All probesets |
| SVA-14 | 2 | Leek | No-correction | Control probesets |
| SVA-15 | 2 | Leek | RMA-Combat | All probesets |
| SVA-16 | 2 | Leek | RMA-Combat | Control probesets |

# Contrasting different SVA approaches for retrieving gene-gene interactions in expression data

The 16 SVA versions were contrasted against each other following the same experimentation protocol described in the main manuscript. We adopted partial correlations [7] instead of the standard correlation measures in order to quantify the linear association between the transcription factor and the other genes *given* the surrogate variables. Particularly, we used the function “zStat” from the R package “pcalg” [8] for calculating partial correlations. Given two variables X and Y and a conditions set CS, this function takes as an input the matrix containing all pairwise correlations between X, Y and CS. We provided in turn the Pearson and Spearman correlation matrix, in order to compute Pearson and Spearman *partial* correlations respectively. It should be note that only the first ones are formally well-defined as correlation measures.

# Results

Figure 1 reports the rank-product scores obtained by the 16 “network-reconstruction SVA” versions. The figure has been produced with the same procedure used for Figure 6 in the main text. Briefly, for each combination of data compendium (E. Coli and Yeast), correlation measure (Pearson and Spearman) and performance metric (AUC, pAUC, AUPRC, AUFDR) the SVA variations are ranked according to their performances, producing a total of 16 different ranks. These ranks are then combined using the Rank-Product method, and the statistical significance of the ranks are evaluated with the method reported in [9]. In Figure 1 the negative logarithm of the Rank-Product score is reported on the y-axis, while SVA variations are listed on the x-axis, along with the No-Correction and RMA-Combat methods. The color of each marker is directly proportional to the Coefficient of Variation (CV) of the respective log-transformed rank-product score, with lighter color corresponding to higher variability (the CV is calculated as the ratio between standard deviation and average value). Methods that tend to be consistently ranked in the top positions are placed on the top-right of the plots, while poorly performing methods remain the in the bottom-left corner.

Overall, variations SVA-1 and SVA-3, corresponding to Modification 1, BE method and estimating the surrogate variables on all genes, are significantly better than the other approaches. Particularly, they both have better performance than the RMA-Combat, even though SVA-1 uses the No-Correction matrix as initial input. This implies that SVA-1 is able to taken in account at least all the systematic biases removed by RMA-Combat.

It is interesting to observe the rank-product scores on the individual data compendia (Figure 2and Figure 3). In E. Coli, SVA-1 and SVA-3 still reach the top of the ranks, while the other approaches does not seem to provide a significant advantage with respect to their respective initial data matrices. In Yeast, the SVA-6 approach obtains outstanding performances, while this same approach is ranked last in E. Coli. We do not have a clear explanation for this result. We note though that SVA-1 and SVA-3 provide results that are better than the ones of RMA-Combat also for this data collection.

Table 2and Table 3 provide the detailed results of the whole experimentation.

# Conclusion

We have proposed and contrasted several approaches for adapting the SVA algorithm for network reconstruction tasks. In the limits of our experimentations, it seems that the SVA variations SVA-1 and SVA-3 consistently provide well-performing results. More studies are needed in order to confirm these findings.

# References

[1] J. T. Leek and J. D. Storey, “Capturing heterogeneity in gene expression studies by surrogate variable analysis.,” *PLoS Genet.*, vol. 3, no. 9, pp. 1724–35, Sep. 2007.

[2] A. Buja and N. Eyuboglu, “Remarks on Parallel Analysis,” *Multivariate Behavioral Research*, vol. 27, no. 4. pp. 509–540, 1992.

[3] J. T. Leek, “Asymptotic Conditional Singular Value Decomposition for High-Dimensional Genomic Data,” *Biometrics*, vol. 67, no. 2, pp. 344–352, 2011.

[4] J. A. Gagnon-Bartsch and T. P. Speed, “Using control genes to correct for unwanted variation in microarray data,” *Biostatistics*, vol. 13, no. 3, pp. 539–552, 2012.

[5] J. T. Leek, “svaseq: removing batch effects and other unwanted noise from sequencing data.,” *Nucleic Acids Res.*, vol. 42, no. 21, p. gku864–, Dec. 2014.

[6] B. Lehner, C. Crombie, J. Tischler, A. Fortunato, and A. G. Fraser, “Systematic mapping of genetic interactions in Caenorhabditis elegans identifies common modifiers of diverse signaling pathways.,” *Nat. Genet.*, vol. 38, no. 8, pp. 896–903, 2006.

[7] R. A. Fisher, “The Distribution of the Partial Correlation Coefficient.,” *Metron*, vol. 3, no. 3–4, pp. 329–332, 1923.

[8] M. Kalisch, M. Machler, D. Colombo, M. H. Maathuis, and P. Buhlmann, “Causal Inference Using Graphical Models with the R Package pcalg,” *J. Stat. Softw.*, vol. 47, no. 11, pp. 1–26, 2012.

[9] T. Heskes, R. Eisinga, and R. Breitling, “A fast algorithm for determining bounds and accurate approximate p-values of the rank product statistic for replicate experiments.,” *BMC Bioinformatics*, vol. 15, no. 1, p. 367, Jan. 2014.

# Figures


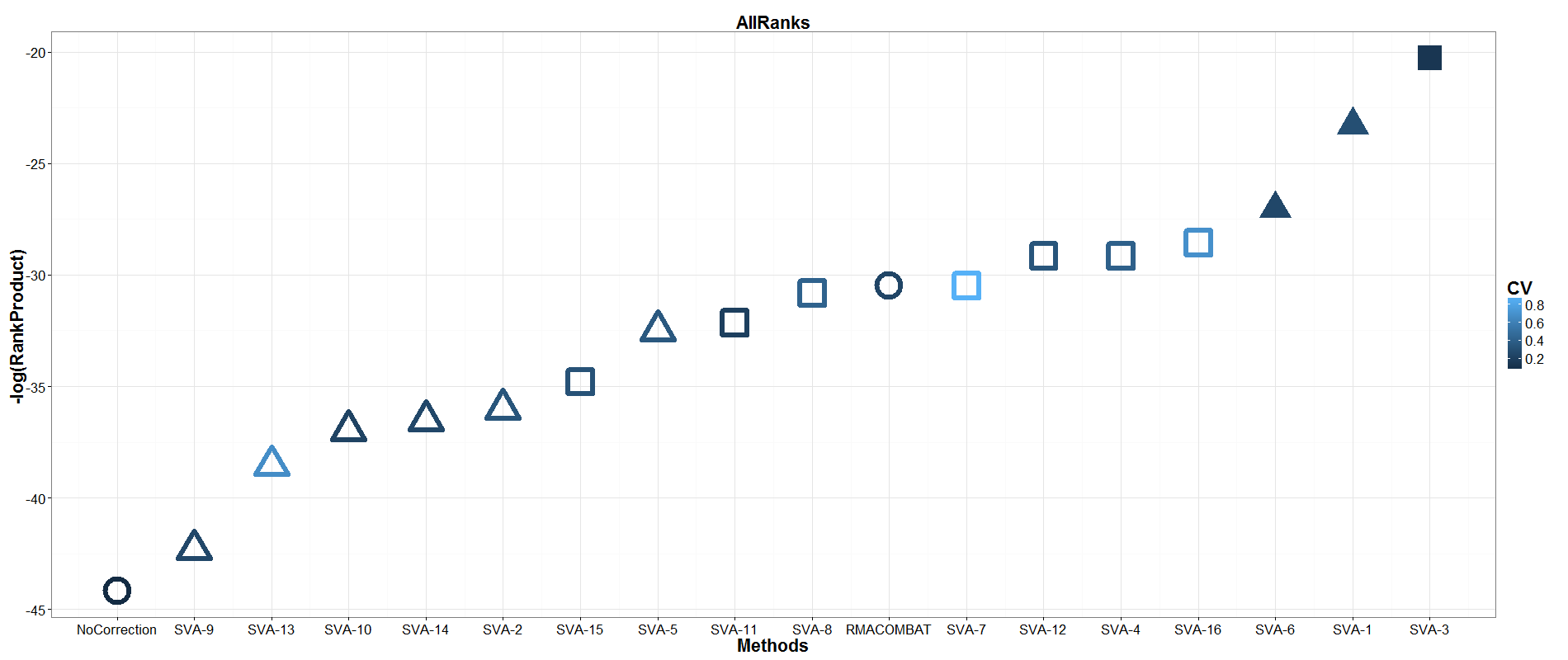


Figure 1: rank-product on both E. Coli and Yeast data collections. Details as in Figure 6 in the main text.


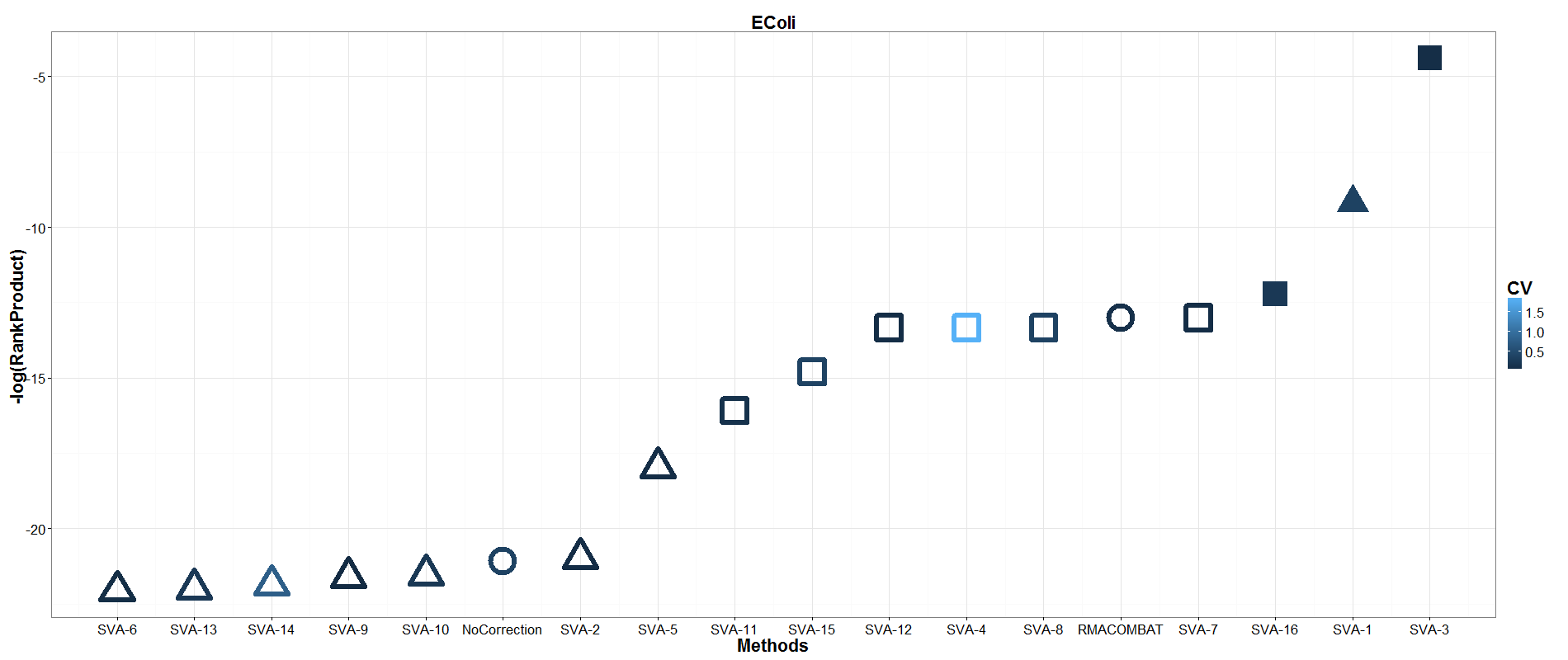


Figure 2: rank-product on E. Coli data collection. Details as in Figure 6 in the main text.


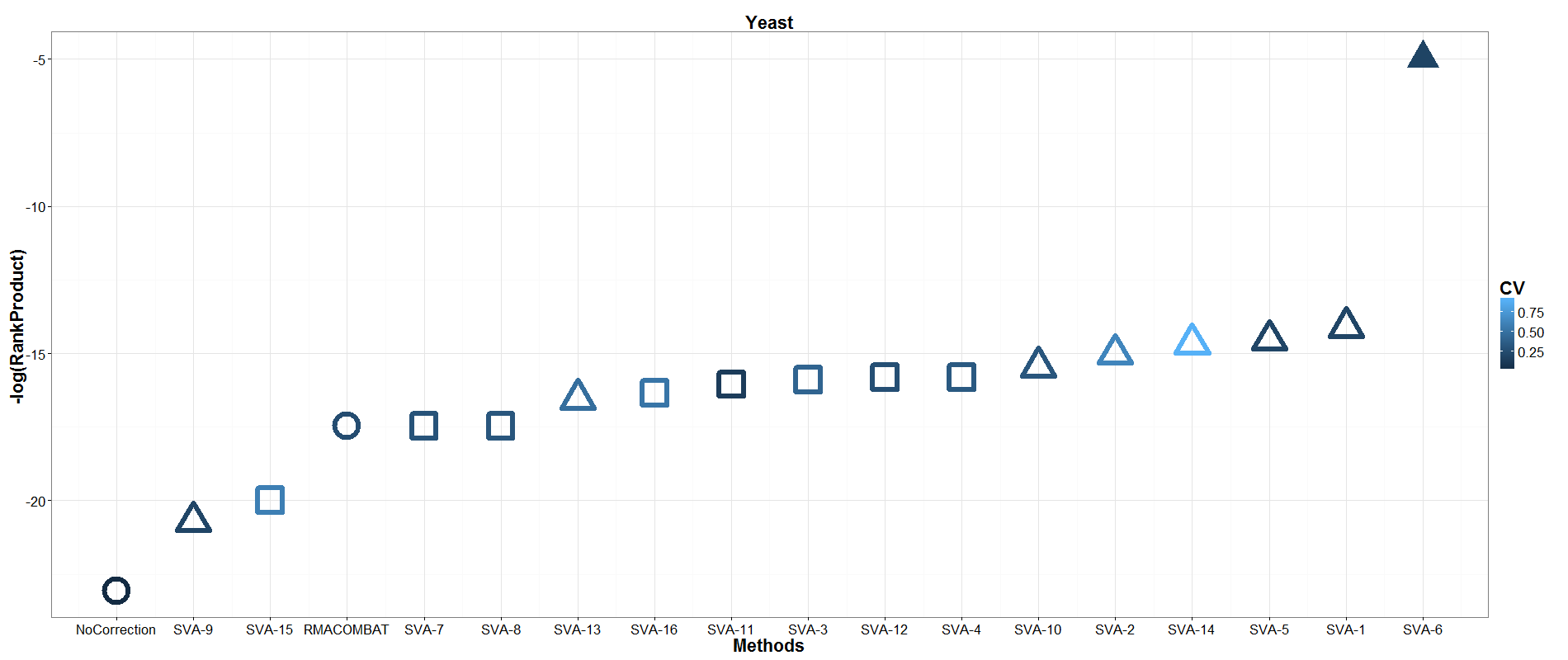


Figure 3: rank-product on Yeast data collection. Details as in Figure 6 in the main text.

# Tables

Table 2: details of the results on the E. Coli compendium. The table is subdivided in eight sub-tables delimited by black borders, one sub-table for each combination of the four metrics (AUC, pAUC, AUPRC and AUFDR) and correlation methods (Pearson and Spearman). Within each sub-table, SVA variations are sorted according to their average performances. One-side, paired t-test p-values are reported as well, comparing each method with the first one and with the method immediately preceding. Performances are averaged over all transcription factors of the data compendium.

| Method | Average Performance | Performance St. Dev. | p-value vs. best method | p-value vs. previous method | Metric | Correlation method |
| --- | --- | --- | --- | --- | --- | --- |
| SVA-4 | 0.6543 | 0.20619 | 1 | 1 | AUC | Pearson |
| SVA-8 | 0.6543 | 0.20619 | NA | NA | AUC | Pearson |
| SVA-12 | 0.6543 | 0.20619 | NA | NA | AUC | Pearson |
| SVA-16 | 0.65304 | 0.20812 | 0.42604 | 0.42604 | AUC | Pearson |
| SVA-1 | 0.6518 | 0.19173 | 0.45123 | 0.47664 | AUC | Pearson |
| SVA-7 | 0.65105 | 0.21093 | 0.35403 | 0.48602 | AUC | Pearson |
| RMACOMBAT | 0.65105 | 0.21093 | 0.35403 | NA | AUC | Pearson |
| SVA-15 | 0.65096 | 0.21097 | 0.35006 | 0.08739 | AUC | Pearson |
| SVA-3 | 0.65046 | 0.17059 | 0.41328 | 0.4892 | AUC | Pearson |
| SVA-5 | 0.64559 | 0.18376 | 0.29121 | 0.38872 | AUC | Pearson |
| SVA-11 | 0.64143 | 0.20902 | 0.06381 | 0.40743 | AUC | Pearson |
| SVA-2 | 0.56653 | 0.22508 | 0 | 0.00002 | AUC | Pearson |
| NoCorrection | 0.56086 | 0.2303 | 0 | 0.28149 | AUC | Pearson |
| SVA-6 | 0.56059 | 0.22709 | 0 | 0.46933 | AUC | Pearson |
| SVA-10 | 0.55832 | 0.2287 | 0 | 0.33561 | AUC | Pearson |
| SVA-14 | 0.55139 | 0.23147 | 0 | 0.09071 | AUC | Pearson |
| SVA-13 | 0.54587 | 0.23267 | 0 | 0.23236 | AUC | Pearson |
| SVA-9 | 0.53316 | 0.22717 | 0 | 0.03497 | AUC | Pearson |
| SVA-3 | 0.66826 | 0.17948 | 1 | 1 | AUC | Spearman |
| SVA-16 | 0.66508 | 0.19747 | 0.41989 | 0.41989 | AUC | Spearman |
| SVA-4 | 0.66376 | 0.2004 | 0.38454 | 0.40748 | AUC | Spearman |
| SVA-8 | 0.66376 | 0.2004 | 0.38454 | NA | AUC | Spearman |
| SVA-12 | 0.66376 | 0.2004 | 0.38454 | NA | AUC | Spearman |
| SVA-7 | 0.65831 | 0.19725 | 0.26416 | 0.26673 | AUC | Spearman |
| RMACOMBAT | 0.65831 | 0.19725 | 0.26416 | NA | AUC | Spearman |
| SVA-15 | 0.65814 | 0.19744 | 0.26068 | 0.22066 | AUC | Spearman |
| SVA-5 | 0.65623 | 0.22741 | 0.25613 | 0.45503 | AUC | Spearman |
| SVA-11 | 0.65099 | 0.19631 | 0.1211 | 0.38026 | AUC | Spearman |
| SVA-1 | 0.64631 | 0.20838 | 0.09314 | 0.4005 | AUC | Spearman |
| NoCorrection | 0.58894 | 0.24177 | 0.00005 | 0.00009 | AUC | Spearman |
| SVA-6 | 0.5873 | 0.2408 | 0.00004 | 0.36486 | AUC | Spearman |
| SVA-10 | 0.58025 | 0.24104 | 0.00001 | 0.10209 | AUC | Spearman |
| SVA-2 | 0.57909 | 0.2344 | 0.00001 | 0.43038 | AUC | Spearman |
| SVA-13 | 0.5783 | 0.23917 | 0 | 0.46909 | AUC | Spearman |
| SVA-14 | 0.57716 | 0.24715 | 0.00001 | 0.4431 | AUC | Spearman |
| SVA-9 | 0.56912 | 0.23723 | 0 | 0.17891 | AUC | Spearman |
| SVA-3 | 0.08637 | 0.1688 | 1 | 1 | AUPRC | Pearson |
| SVA-1 | 0.08398 | 0.16291 | 0.38511 | 0.38511 | AUPRC | Pearson |
| SVA-7 | 0.07719 | 0.16008 | 0.21484 | 0.30753 | AUPRC | Pearson |
| RMACOMBAT | 0.07719 | 0.16008 | 0.21484 | NA | AUPRC | Pearson |
| SVA-15 | 0.07713 | 0.1601 | 0.21352 | 0.07083 | AUPRC | Pearson |
| SVA-16 | 0.0767 | 0.154 | 0.20038 | 0.41369 | AUPRC | Pearson |
| SVA-11 | 0.07345 | 0.15391 | 0.12595 | 0.23223 | AUPRC | Pearson |
| SVA-4 | 0.07156 | 0.14635 | 0.10489 | 0.35522 | AUPRC | Pearson |
| SVA-8 | 0.07156 | 0.14635 | 0.10489 | NA | AUPRC | Pearson |
| SVA-12 | 0.07156 | 0.14635 | 0.10489 | NA | AUPRC | Pearson |
| SVA-5 | 0.05728 | 0.12751 | 0.00925 | 0.05172 | AUPRC | Pearson |
| SVA-9 | 0.04911 | 0.12677 | 0.00094 | 0.17356 | AUPRC | Pearson |
| SVA-2 | 0.04351 | 0.1247 | 0.00171 | 0.23356 | AUPRC | Pearson |
| NoCorrection | 0.0429 | 0.12339 | 0.00224 | 0.41135 | AUPRC | Pearson |
| SVA-10 | 0.04232 | 0.12403 | 0.00158 | 0.35029 | AUPRC | Pearson |
| SVA-14 | 0.0416 | 0.12121 | 0.00104 | 0.29734 | AUPRC | Pearson |
| SVA-6 | 0.04085 | 0.12048 | 0.00115 | 0.27056 | AUPRC | Pearson |
| SVA-13 | 0.0408 | 0.11555 | 0.001 | 0.49328 | AUPRC | Pearson |
| SVA-3 | 0.09131 | 0.1687 | 1 | 1 | AUPRC | Spearman |
| SVA-1 | 0.07157 | 0.15577 | 0.01057 | 0.01057 | AUPRC | Spearman |
| SVA-7 | 0.0667 | 0.1348 | 0.0165 | 0.34103 | AUPRC | Spearman |
| RMACOMBAT | 0.0667 | 0.1348 | 0.0165 | NA | AUPRC | Spearman |
| SVA-15 | 0.06666 | 0.13481 | 0.01637 | 0.18583 | AUPRC | Spearman |
| SVA-11 | 0.0657 | 0.12836 | 0.0106 | 0.43182 | AUPRC | Spearman |
| SVA-16 | 0.06553 | 0.13179 | 0.01413 | 0.4885 | AUPRC | Spearman |
| SVA-5 | 0.06277 | 0.14098 | 0.00089 | 0.39655 | AUPRC | Spearman |
| SVA-4 | 0.06248 | 0.12443 | 0.00685 | 0.48874 | AUPRC | Spearman |
| SVA-8 | 0.06248 | 0.12443 | 0.00685 | NA | AUPRC | Spearman |
| SVA-12 | 0.06248 | 0.12443 | 0.00685 | NA | AUPRC | Spearman |
| SVA-14 | 0.0575 | 0.14295 | 0.00046 | 0.29218 | AUPRC | Spearman |
| SVA-2 | 0.05702 | 0.14516 | 0.00042 | 0.38208 | AUPRC | Spearman |
| SVA-10 | 0.05458 | 0.14008 | 0.00035 | 0.23154 | AUPRC | Spearman |
| SVA-9 | 0.05387 | 0.1335 | 0.00059 | 0.45628 | AUPRC | Spearman |
| NoCorrection | 0.05287 | 0.1363 | 0.00089 | 0.44093 | AUPRC | Spearman |
| SVA-6 | 0.05187 | 0.13335 | 0.0004 | 0.24197 | AUPRC | Spearman |
| SVA-13 | 0.05088 | 0.12887 | 0.00048 | 0.36582 | AUPRC | Spearman |
| SVA-1 | 0.64061 | 0.15798 | 1 | 1 | pAUC | Pearson |
| SVA-4 | 0.63355 | 0.16376 | 0.30449 | 0.30449 | pAUC | Pearson |
| SVA-8 | 0.63355 | 0.16376 | 0.30449 | NA | pAUC | Pearson |
| SVA-12 | 0.63355 | 0.16376 | 0.30449 | NA | pAUC | Pearson |
| SVA-16 | 0.63292 | 0.15921 | 0.28889 | 0.42591 | pAUC | Pearson |
| SVA-7 | 0.63109 | 0.1596 | 0.24704 | 0.1291 | pAUC | Pearson |
| RMACOMBAT | 0.63109 | 0.1596 | 0.24704 | NA | pAUC | Pearson |
| SVA-15 | 0.63092 | 0.15974 | 0.24327 | 0.05739 | pAUC | Pearson |
| SVA-3 | 0.62979 | 0.15592 | 0.16873 | 0.46417 | pAUC | Pearson |
| SVA-11 | 0.62146 | 0.15637 | 0.07663 | 0.24124 | pAUC | Pearson |
| SVA-5 | 0.60729 | 0.14723 | 0.01323 | 0.12249 | pAUC | Pearson |
| NoCorrection | 0.5687 | 0.14167 | 0 | 0.0024 | pAUC | Pearson |
| SVA-13 | 0.56793 | 0.14136 | 0 | 0.37732 | pAUC | Pearson |
| SVA-2 | 0.56693 | 0.14132 | 0 | 0.44421 | pAUC | Pearson |
| SVA-10 | 0.56465 | 0.14003 | 0 | 0.34712 | pAUC | Pearson |
| SVA-6 | 0.56328 | 0.13987 | 0 | 0.33522 | pAUC | Pearson |
| SVA-9 | 0.56317 | 0.13835 | 0 | 0.49048 | pAUC | Pearson |
| SVA-14 | 0.56026 | 0.13739 | 0 | 0.27274 | pAUC | Pearson |
| SVA-3 | 0.64124 | 0.15855 | 1 | 1 | pAUC | Spearman |
| SVA-16 | 0.63384 | 0.15788 | 0.22992 | 0.22992 | pAUC | Spearman |
| SVA-4 | 0.63313 | 0.16189 | 0.19877 | 0.42315 | pAUC | Spearman |
| SVA-8 | 0.63313 | 0.16189 | 0.19877 | NA | pAUC | Spearman |
| SVA-12 | 0.63313 | 0.16189 | 0.19877 | NA | pAUC | Spearman |
| SVA-5 | 0.63129 | 0.15794 | 0.18674 | 0.42774 | pAUC | Spearman |
| SVA-15 | 0.63101 | 0.15763 | 0.14813 | 0.48857 | pAUC | Spearman |
| SVA-7 | 0.63088 | 0.15766 | 0.14479 | 0.36879 | pAUC | Spearman |
| RMACOMBAT | 0.63088 | 0.15766 | 0.14479 | NA | pAUC | Spearman |
| SVA-11 | 0.62907 | 0.1587 | 0.09638 | 0.26412 | pAUC | Spearman |
| SVA-1 | 0.62473 | 0.16439 | 0.03907 | 0.34935 | pAUC | Spearman |
| NoCorrection | 0.59206 | 0.1522 | 0.00004 | 0.00106 | pAUC | Spearman |
| SVA-13 | 0.59037 | 0.15387 | 0.00003 | 0.29028 | pAUC | Spearman |
| SVA-6 | 0.5902 | 0.15522 | 0.00004 | 0.48057 | pAUC | Spearman |
| SVA-10 | 0.58731 | 0.15453 | 0.00002 | 0.16114 | pAUC | Spearman |
| SVA-14 | 0.58542 | 0.15486 | 0.00001 | 0.27787 | pAUC | Spearman |
| SVA-9 | 0.58468 | 0.15212 | 0 | 0.43718 | pAUC | Spearman |
| SVA-2 | 0.58231 | 0.14697 | 0.00001 | 0.37511 | pAUC | Spearman |
| SVA-3 | 0.19047 | 0.237 | 1 | 1 | AUFDR | Pearson |
| SVA-1 | 0.17939 | 0.23384 | 0.16527 | 0.16527 | AUFDR | Pearson |
| SVA-7 | 0.15616 | 0.2272 | 0.01208 | 0.07538 | AUFDR | Pearson |
| RMACOMBAT | 0.15616 | 0.2272 | 0.01208 | NA | AUFDR | Pearson |
| SVA-15 | 0.15577 | 0.22737 | 0.01131 | 0.15366 | AUFDR | Pearson |
| SVA-16 | 0.15574 | 0.22563 | 0.01206 | 0.4933 | AUFDR | Pearson |
| SVA-11 | 0.1526 | 0.2252 | 0.00463 | 0.28928 | AUFDR | Pearson |
| SVA-4 | 0.15145 | 0.21858 | 0.00538 | 0.43787 | AUFDR | Pearson |
| SVA-8 | 0.15145 | 0.21858 | 0.00538 | NA | AUFDR | Pearson |
| SVA-12 | 0.15145 | 0.21858 | 0.00538 | NA | AUFDR | Pearson |
| SVA-5 | 0.13166 | 0.20584 | 0.00019 | 0.04722 | AUFDR | Pearson |
| SVA-9 | 0.10158 | 0.18397 | 0 | 0.0081 | AUFDR | Pearson |
| SVA-2 | 0.09371 | 0.18668 | 0 | 0.12459 | AUFDR | Pearson |
| SVA-10 | 0.08903 | 0.17881 | 0 | 0.11261 | AUFDR | Pearson |
| SVA-14 | 0.08818 | 0.17743 | 0 | 0.38917 | AUFDR | Pearson |
| SVA-13 | 0.08787 | 0.17489 | 0 | 0.47199 | AUFDR | Pearson |
| NoCorrection | 0.08711 | 0.17757 | 0 | 0.4025 | AUFDR | Pearson |
| SVA-6 | 0.08462 | 0.17254 | 0 | 0.18697 | AUFDR | Pearson |
| SVA-3 | 0.19499 | 0.245 | 1 | 1 | AUFDR | Spearman |
| SVA-1 | 0.16228 | 0.22773 | 0.00328 | 0.00328 | AUFDR | Spearman |
| SVA-11 | 0.15166 | 0.21815 | 0.0003 | 0.2071 | AUFDR | Spearman |
| SVA-7 | 0.14759 | 0.21766 | 0.00035 | 0.25332 | AUFDR | Spearman |
| RMACOMBAT | 0.14759 | 0.21766 | 0.00035 | NA | AUFDR | Spearman |
| SVA-15 | 0.14753 | 0.21768 | 0.00034 | 0.26824 | AUFDR | Spearman |
| SVA-4 | 0.14483 | 0.20811 | 0.00015 | 0.26664 | AUFDR | Spearman |
| SVA-8 | 0.14483 | 0.20811 | 0.00015 | NA | AUFDR | Spearman |
| SVA-12 | 0.14483 | 0.20811 | 0.00015 | NA | AUFDR | Spearman |
| SVA-16 | 0.14435 | 0.21217 | 0.00016 | 0.42099 | AUFDR | Spearman |
| SVA-5 | 0.13815 | 0.21382 | 0.00001 | 0.28718 | AUFDR | Spearman |
| SVA-9 | 0.11764 | 0.20233 | 0 | 0.00817 | AUFDR | Spearman |
| SVA-2 | 0.11561 | 0.20857 | 0 | 0.38393 | AUFDR | Spearman |
| SVA-14 | 0.11533 | 0.20501 | 0 | 0.47032 | AUFDR | Spearman |
| SVA-13 | 0.11526 | 0.19852 | 0 | 0.4945 | AUFDR | Spearman |
| SVA-10 | 0.11308 | 0.20858 | 0 | 0.34112 | AUFDR | Spearman |
| NoCorrection | 0.11129 | 0.20158 | 0 | 0.35243 | AUFDR | Spearman |
| SVA-6 | 0.11126 | 0.20057 | 0 | 0.49576 | AUFDR | Spearman |

Table 3: details of the results on the Yeast compendium. Details as in Table 2.

| Method | Average Performance | Performance St. Dev. | p-value vs. best method | p-value vs. previous method | Metric | Correlation method |
| --- | --- | --- | --- | --- | --- | --- |
| SVA-16 | 0.60453 | 0.16469 | 1 | 1 | AUC | Pearson |
| SVA-6 | 0.60338 | 0.19605 | 0.479 | 0.479 | AUC | Pearson |
| SVA-4 | 0.60231 | 0.15527 | 0.26294 | 0.47988 | AUC | Pearson |
| SVA-12 | 0.60231 | 0.15527 | 0.26294 | NA | AUC | Pearson |
| SVA-7 | 0.60037 | 0.16394 | 0.28266 | 0.40073 | AUC | Pearson |
| SVA-8 | 0.60037 | 0.16394 | 0.28266 | NA | AUC | Pearson |
| RMACOMBAT | 0.60037 | 0.16394 | 0.28266 | NA | AUC | Pearson |
| SVA-14 | 0.59971 | 0.18355 | 0.42252 | 0.48969 | AUC | Pearson |
| SVA-15 | 0.59439 | 0.16998 | 0.12481 | 0.41645 | AUC | Pearson |
| SVA-11 | 0.58719 | 0.16023 | 0.0483 | 0.11991 | AUC | Pearson |
| SVA-2 | 0.58533 | 0.19472 | 0.20352 | 0.46795 | AUC | Pearson |
| SVA-10 | 0.5818 | 0.1956 | 0.16075 | 0.38821 | AUC | Pearson |
| SVA-3 | 0.5619 | 0.16464 | 0.09378 | 0.27019 | AUC | Pearson |
| SVA-5 | 0.55989 | 0.15859 | 0.01365 | 0.47207 | AUC | Pearson |
| SVA-1 | 0.54991 | 0.15389 | 0.00548 | 0.13713 | AUC | Pearson |
| SVA-9 | 0.50157 | 0.2114 | 0.00011 | 0.04446 | AUC | Pearson |
| SVA-13 | 0.49326 | 0.20787 | 0.00015 | 0.36461 | AUC | Pearson |
| NoCorrection | 0.47817 | 0.19389 | 0.00001 | 0.21214 | AUC | Pearson |
| SVA-6 | 0.60751 | 0.18133 | 1 | 1 | AUC | Spearman |
| SVA-4 | 0.59255 | 0.1578 | 0.25362 | 0.25362 | AUC | Spearman |
| SVA-12 | 0.59255 | 0.1578 | 0.25362 | NA | AUC | Spearman |
| SVA-7 | 0.5918 | 0.16207 | 0.25388 | 0.45608 | AUC | Spearman |
| SVA-8 | 0.5918 | 0.16207 | 0.25388 | NA | AUC | Spearman |
| RMACOMBAT | 0.5918 | 0.16207 | 0.25388 | NA | AUC | Spearman |
| SVA-16 | 0.59005 | 0.16474 | 0.22686 | 0.39972 | AUC | Spearman |
| SVA-15 | 0.58716 | 0.16828 | 0.20058 | 0.33741 | AUC | Spearman |
| SVA-14 | 0.58193 | 0.18755 | 0.08439 | 0.42598 | AUC | Spearman |
| SVA-11 | 0.58108 | 0.15602 | 0.14125 | 0.48683 | AUC | Spearman |
| SVA-2 | 0.58018 | 0.17548 | 0.05604 | 0.48471 | AUC | Spearman |
| SVA-1 | 0.57934 | 0.18016 | 0.05998 | 0.48581 | AUC | Spearman |
| SVA-10 | 0.57618 | 0.17351 | 0.03738 | 0.44649 | AUC | Spearman |
| SVA-9 | 0.55582 | 0.19341 | 0.01007 | 0.1556 | AUC | Spearman |
| SVA-3 | 0.55296 | 0.1833 | 0.06289 | 0.46548 | AUC | Spearman |
| SVA-5 | 0.54561 | 0.18444 | 0.00017 | 0.41771 | AUC | Spearman |
| SVA-13 | 0.54257 | 0.18729 | 0.00024 | 0.43936 | AUC | Spearman |
| NoCorrection | 0.53425 | 0.18923 | 0.00046 | 0.21381 | AUC | Spearman |
| SVA-6 | 0.01374 | 0.03993 | 1 | 1 | AUPRC | Pearson |
| SVA-5 | 0.01367 | 0.03481 | 0.49292 | 0.49292 | AUPRC | Pearson |
| SVA-1 | 0.01253 | 0.03127 | 0.37707 | 0.13402 | AUPRC | Pearson |
| SVA-13 | 0.0118 | 0.05753 | 0.401 | 0.46074 | AUPRC | Pearson |
| SVA-3 | 0.01119 | 0.03976 | 0.29781 | 0.45014 | AUPRC | Pearson |
| SVA-11 | 0.01062 | 0.03734 | 0.26377 | 0.37674 | AUPRC | Pearson |
| SVA-10 | 0.00995 | 0.02877 | 0.05102 | 0.42343 | AUPRC | Pearson |
| SVA-2 | 0.00965 | 0.0288 | 0.0377 | 0.06769 | AUPRC | Pearson |
| SVA-14 | 0.00782 | 0.02 | 0.0628 | 0.18617 | AUPRC | Pearson |
| SVA-4 | 0.00759 | 0.02369 | 0.09112 | 0.42709 | AUPRC | Pearson |
| SVA-12 | 0.00759 | 0.02369 | 0.09112 | NA | AUPRC | Pearson |
| SVA-16 | 0.0066 | 0.01877 | 0.06507 | 0.12689 | AUPRC | Pearson |
| SVA-7 | 0.00657 | 0.01879 | 0.06113 | 0.45838 | AUPRC | Pearson |
| SVA-8 | 0.00657 | 0.01879 | 0.06113 | NA | AUPRC | Pearson |
| RMACOMBAT | 0.00657 | 0.01879 | 0.06113 | NA | AUPRC | Pearson |
| SVA-15 | 0.00614 | 0.01748 | 0.05624 | 0.10079 | AUPRC | Pearson |
| SVA-9 | 0.00447 | 0.01094 | 0.04271 | 0.10942 | AUPRC | Pearson |
| NoCorrection | 0.00288 | 0.00753 | 0.02405 | 0.09275 | AUPRC | Pearson |
| SVA-13 | 0.01137 | 0.05597 | 1 | 1 | AUPRC | Spearman |
| SVA-3 | 0.01026 | 0.04046 | 0.37599 | 0.37599 | AUPRC | Spearman |
| SVA-11 | 0.00937 | 0.03114 | 0.34165 | 0.32375 | AUPRC | Spearman |
| SVA-6 | 0.00846 | 0.02444 | 0.30637 | 0.2715 | AUPRC | Spearman |
| SVA-4 | 0.00782 | 0.02256 | 0.29297 | 0.27096 | AUPRC | Spearman |
| SVA-12 | 0.00782 | 0.02256 | 0.29297 | NA | AUPRC | Spearman |
| SVA-1 | 0.00778 | 0.02247 | 0.26717 | 0.4881 | AUPRC | Spearman |
| SVA-2 | 0.00772 | 0.02148 | 0.28238 | 0.47819 | AUPRC | Spearman |
| SVA-10 | 0.00767 | 0.0215 | 0.2797 | 0.19787 | AUPRC | Spearman |
| SVA-16 | 0.00751 | 0.02112 | 0.27496 | 0.45186 | AUPRC | Spearman |
| SVA-5 | 0.00749 | 0.02332 | 0.23492 | 0.49457 | AUPRC | Spearman |
| SVA-7 | 0.00731 | 0.02023 | 0.26748 | 0.45107 | AUPRC | Spearman |
| SVA-8 | 0.00731 | 0.02023 | 0.26748 | NA | AUPRC | Spearman |
| RMACOMBAT | 0.00731 | 0.02023 | 0.26748 | NA | AUPRC | Spearman |
| SVA-14 | 0.00695 | 0.02057 | 0.23347 | 0.32973 | AUPRC | Spearman |
| SVA-15 | 0.00686 | 0.0193 | 0.24402 | 0.45019 | AUPRC | Spearman |
| SVA-9 | 0.00631 | 0.01669 | 0.22331 | 0.25364 | AUPRC | Spearman |
| NoCorrection | 0.00478 | 0.01366 | 0.16778 | 0.10644 | AUPRC | Spearman |
| SVA-6 | 0.5651 | 0.13221 | 1 | 1 | pAUC | Pearson |
| SVA-14 | 0.5617 | 0.12589 | 0.37651 | 0.37651 | pAUC | Pearson |
| SVA-10 | 0.56075 | 0.12261 | 0.35105 | 0.4572 | pAUC | Pearson |
| SVA-2 | 0.55679 | 0.12067 | 0.17537 | 0.3074 | pAUC | Pearson |
| SVA-7 | 0.55477 | 0.11181 | 0.22328 | 0.43374 | pAUC | Pearson |
| SVA-8 | 0.55477 | 0.11181 | 0.22328 | NA | pAUC | Pearson |
| RMACOMBAT | 0.55477 | 0.11181 | 0.22328 | NA | pAUC | Pearson |
| SVA-15 | 0.55298 | 0.11097 | 0.18749 | 0.23477 | pAUC | Pearson |
| SVA-16 | 0.54949 | 0.11426 | 0.10945 | 0.26621 | pAUC | Pearson |
| SVA-4 | 0.54572 | 0.11511 | 0.07508 | 0.07517 | pAUC | Pearson |
| SVA-12 | 0.54572 | 0.11511 | 0.07508 | NA | pAUC | Pearson |
| SVA-11 | 0.54555 | 0.11518 | 0.09582 | 0.48957 | pAUC | Pearson |
| SVA-3 | 0.54398 | 0.1061 | 0.11979 | 0.45667 | pAUC | Pearson |
| SVA-9 | 0.53808 | 0.12724 | 0.01432 | 0.35471 | pAUC | Pearson |
| SVA-5 | 0.53641 | 0.12305 | 0.00541 | 0.45053 | pAUC | Pearson |
| SVA-1 | 0.5299 | 0.12035 | 0.00203 | 0.07802 | pAUC | Pearson |
| SVA-13 | 0.52469 | 0.11777 | 0.00148 | 0.36487 | pAUC | Pearson |
| NoCorrection | 0.51309 | 0.10614 | 0.00026 | 0.14557 | pAUC | Pearson |
| SVA-14 | 0.55798 | 0.13418 | 1 | 1 | pAUC | Spearman |
| SVA-6 | 0.55709 | 0.12619 | 0.44404 | 0.44404 | pAUC | Spearman |
| SVA-2 | 0.55443 | 0.12749 | 0.16982 | 0.32231 | pAUC | Spearman |
| SVA-9 | 0.55251 | 0.12983 | 0.23475 | 0.38824 | pAUC | Spearman |
| SVA-10 | 0.55239 | 0.12902 | 0.05214 | 0.49305 | pAUC | Spearman |
| SVA-7 | 0.55014 | 0.11195 | 0.26312 | 0.42483 | pAUC | Spearman |
| SVA-8 | 0.55014 | 0.11195 | 0.26312 | NA | pAUC | Spearman |
| RMACOMBAT | 0.55014 | 0.11195 | 0.26312 | NA | pAUC | Spearman |
| SVA-3 | 0.54801 | 0.10973 | 0.28639 | 0.4451 | pAUC | Spearman |
| SVA-16 | 0.54751 | 0.11301 | 0.20507 | 0.48692 | pAUC | Spearman |
| SVA-1 | 0.54713 | 0.12691 | 0.17402 | 0.48578 | pAUC | Spearman |
| SVA-15 | 0.54596 | 0.11119 | 0.16534 | 0.44965 | pAUC | Spearman |
| SVA-13 | 0.54499 | 0.11996 | 0.07188 | 0.46658 | pAUC | Spearman |
| SVA-5 | 0.54419 | 0.12396 | 0.09689 | 0.47159 | pAUC | Spearman |
| SVA-4 | 0.54251 | 0.11345 | 0.12989 | 0.43779 | pAUC | Spearman |
| SVA-12 | 0.54251 | 0.11345 | 0.12989 | NA | pAUC | Spearman |
| NoCorrection | 0.53982 | 0.12513 | 0.01519 | 0.42726 | pAUC | Spearman |
| SVA-11 | 0.53803 | 0.11596 | 0.09951 | 0.45457 | pAUC | Spearman |
| SVA-5 | 0.04932 | 0.11676 | 1 | 1 | AUFDR | Pearson |
| SVA-1 | 0.04784 | 0.1136 | 0.16635 | 0.16635 | AUFDR | Pearson |
| SVA-6 | 0.03943 | 0.11396 | 0.12577 | 0.16122 | AUFDR | Pearson |
| SVA-11 | 0.03369 | 0.11118 | 0.1255 | 0.31235 | AUFDR | Pearson |
| SVA-10 | 0.03357 | 0.09933 | 0.05915 | 0.49531 | AUFDR | Pearson |
| SVA-13 | 0.03245 | 0.1233 | 0.11147 | 0.4544 | AUFDR | Pearson |
| SVA-3 | 0.03239 | 0.11337 | 0.09095 | 0.49722 | AUFDR | Pearson |
| SVA-14 | 0.03102 | 0.0878 | 0.03746 | 0.45237 | AUFDR | Pearson |
| SVA-2 | 0.03068 | 0.09874 | 0.02678 | 0.46622 | AUFDR | Pearson |
| SVA-4 | 0.02987 | 0.09373 | 0.03496 | 0.44994 | AUFDR | Pearson |
| SVA-12 | 0.02987 | 0.09373 | 0.03496 | NA | AUFDR | Pearson |
| SVA-7 | 0.02822 | 0.08633 | 0.0266 | 0.13674 | AUFDR | Pearson |
| SVA-8 | 0.02822 | 0.08633 | 0.0266 | NA | AUFDR | Pearson |
| RMACOMBAT | 0.02822 | 0.08633 | 0.0266 | NA | AUFDR | Pearson |
| SVA-16 | 0.02791 | 0.08598 | 0.02635 | 0.26968 | AUFDR | Pearson |
| SVA-15 | 0.02708 | 0.08308 | 0.02198 | 0.13094 | AUFDR | Pearson |
| SVA-9 | 0.02612 | 0.06035 | 0.03685 | 0.4344 | AUFDR | Pearson |
| NoCorrection | 0.01865 | 0.0533 | 0.0094 | 0.0832 | AUFDR | Pearson |
| SVA-1 | 0.03347 | 0.09183 | 1 | 1 | AUFDR | Spearman |
| SVA-5 | 0.0319 | 0.0916 | 0.30422 | 0.30422 | AUFDR | Spearman |
| SVA-6 | 0.03172 | 0.09709 | 0.35008 | 0.48506 | AUFDR | Spearman |
| SVA-2 | 0.03017 | 0.09332 | 0.32558 | 0.38486 | AUFDR | Spearman |
| SVA-3 | 0.0301 | 0.11091 | 0.31218 | 0.49706 | AUFDR | Spearman |
| SVA-11 | 0.0298 | 0.10458 | 0.26472 | 0.47967 | AUFDR | Spearman |
| SVA-10 | 0.02968 | 0.09346 | 0.30409 | 0.49457 | AUFDR | Spearman |
| SVA-4 | 0.02886 | 0.08999 | 0.21227 | 0.45862 | AUFDR | Spearman |
| SVA-12 | 0.02886 | 0.08999 | 0.21227 | NA | AUFDR | Spearman |
| SVA-13 | 0.0288 | 0.1144 | 0.26683 | 0.49743 | AUFDR | Spearman |
| SVA-16 | 0.02844 | 0.08754 | 0.19257 | 0.485 | AUFDR | Spearman |
| SVA-7 | 0.02833 | 0.08597 | 0.18794 | 0.40526 | AUFDR | Spearman |
| SVA-8 | 0.02833 | 0.08597 | 0.18794 | NA | AUFDR | Spearman |
| RMACOMBAT | 0.02833 | 0.08597 | 0.18794 | NA | AUFDR | Spearman |
| SVA-14 | 0.02797 | 0.08633 | 0.15363 | 0.47505 | AUFDR | Spearman |
| SVA-9 | 0.02767 | 0.07697 | 0.1194 | 0.476 | AUFDR | Spearman |
| SVA-15 | 0.02605 | 0.08353 | 0.09521 | 0.37915 | AUFDR | Spearman |
| NoCorrection | 0.02038 | 0.06614 | 0.02898 | 0.23328 | AUFDR | Spearman |
